# RNA structure promotes liquid-to-solid phase transition of short RNAs in neuronal dysfunction

**DOI:** 10.1038/s42003-024-05828-z

**Published:** 2024-01-29

**Authors:** Shiyu Wang, Yan Xu

**Affiliations:** Division of Chemistry, Department of Medical Sciences, Faculty of Medicine, University of Miyazaki, 5200 Kihara, Kiyotake, Miyazaki, 889-1692 Japan

**Keywords:** RNA, RNA

## Abstract

In nucleotide expansion disorders, RNA foci are reportedly associated with neurodegenerative disease pathogeneses. Characteristically, these RNAs exhibit long poly-RNA repeats, such as 47 × CAG, 47 × CUG, or 29 × GGGGCC, usually becoming abnormal pathological aggregations above a critical number of nucleotide repeats. However, it remains unclear whether short, predominantly cellular RNA molecules can cause phase transitions to induce RNA foci. Herein, we demonstrated that short RNAs even with only two repeats can aggregate into a solid-like state via special RNA G-quadruplex structures. In human cells, these solid RNA foci could not dissolve even when using agents that disrupt RNA gelation. The aggregation of shorter RNAs can be clearly observed in vivo. Furthermore, we found that RNA foci induce colocalization of the RNA-binding protein Sam68, a protein commonly found in patients with fragile X-associated tremor/ataxia syndrome, suppressing cell clonogenicity and eventually causing cell death. Our results suggest that short RNA gelation promoted by specific RNA structures contribute to the neurological diseases, which disturb functional cellular processes.

## Abstract (web summary)

Specially folded G-quadruplex of short RNAs have the capacity to form solid-like aggregates. The aggregation of these short RNAs is clearly observable both in cells and in vivo, resulting in the suppression of cell clonogenicity and eventual cell death.

## Introduction

RNA foci constitute a common characteristic of numerous neurodegenerative diseases^1–4^. RNA foci formation is supported by RNA phase separation and transition, which further sequester or disrupt functional proteins that regulate the nervous system^5–7^. The prevalence of RNA foci in nucleotide repeat expansion disorders clearly indicates RNA assemble-toxicity^8–14^. Fragile X syndrome (FXS) is caused by CGG trinucleotide repeat expansion in the fragile X mental retardation 1 (*FMR1*) gene^15,16^. Hexanucleotide GGGGCC repeat expansion in *C9ORF72* has been linked to amyotrophic lateral sclerosis (ALS) and frontotemporal dementia (FTD)^17^. These RNA sequences with high GC content trigger phase separation, transition, and even the formation of gel-like nuclear RNA foci^9,18^.

In addition to the amount^19^, sequence^9^, length^9^, and secondary structure of the RNA^20,21^, the relative salt and molecular crowding conditions largely affect the ability of RNA to form RNA foci^5,22,23^. To date, the self-assembly of repeat expansion RNAs was believed to be associated with their length as longer RNAs possess more sites for RNA-RNA interactions^5,9,24,25^. However, little is known about the molecular mechanism by which a large number of short nucleotide repeats results in formation of RNA foci.

G-quadruplexes are four-stranded DNA or RNA structures comprising G-tetrads formed by the hydrogen bonding of four guanines^26,27^. It is well known that short RNA sequences form RNA G-quadruplexes with stable structures^26–29^. In the previous studies, we demonstrated that G-quadruplex structures formed from short 8-mer RNA remained stable against base pair hydrogen-bond disruption via urea under denaturing conditions^30^. Indeed, short RNAs may exist in a wide range of lengths compared with long RNAs owing to RNA digestion via different cellular RNases^31–33^. Recently, RNA chain length was demonstrated to exhibit a major stabilizing effect on protein-RNA complex condensation^34,35^.

These observations indicate a novel hypothesis that an RNA G-quadruplex formed by short RNAs can trigger RNA phase separation and transition, contributing to higher-order RNA assemblies. Herein, we tested the hypothesis whether two shorter CGG and GGGGCC repeats associated with FXS and ALS and FTD pathogeneses adopt a specific RNA structure, e.g., G-quadruplex, to allow RNA-driven phase transition and promote RNA foci solidification.

## Results

### Self-assembly of short RNA sequences into droplets and solids in vitro

**Fig. 1:**
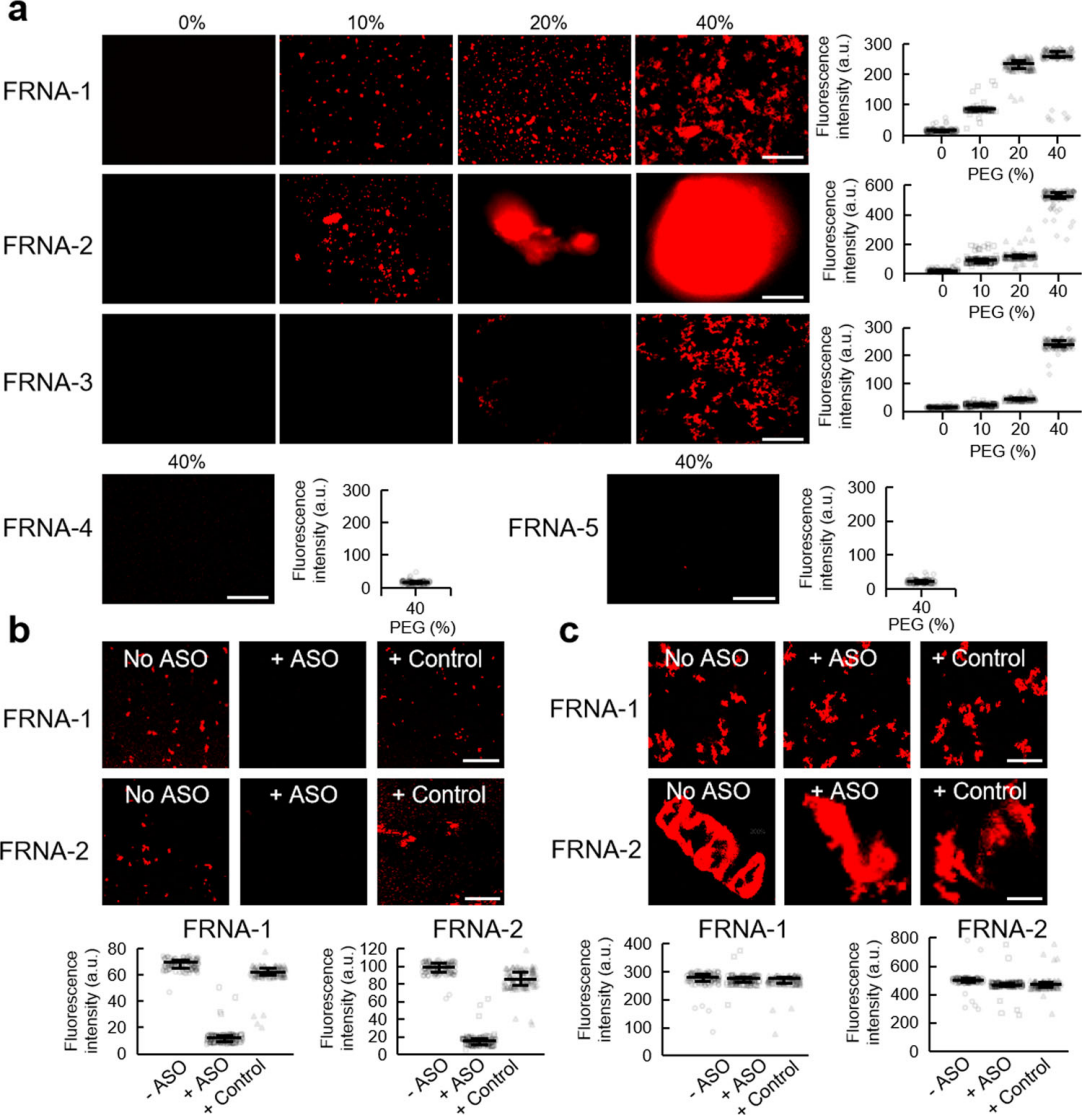
Short RNAs induce aggregation in vitro. **a** Fluorescence micrographs for indicated FRNA-1, FRNA-2, FRNA-3, FRNA-4, and FRNA-5 with different PEG 200 concentrations (0–40% PEG) (*n* = 120). **b** Effects of ASO and control on FRNA-1 or FRNA-2 clustering with 10% PEG (*n* = 120). **c** Effects of ASO and control on FRNA-1 or FRNA-2 clustering with 40% PEG (*n* = 120). Details of data acquisition are described in Methods. Error bars represent mean ± interquartile. Scale bars, 50 µm.

To examine whether short RNAs assemble into large clusters, we synthesized a series of short RNAs with and without fluorescent labels (Supplementary Table 1), of which some sequences have previously been shown to form G-quadruplex structures^36–39^. These sequences included two CGG repeated 8-mer r(GCGGCGGC) RNA-1/FRNA-1 (fluorescent Cy3-labeled), fluorinated r(GC^F^GGCGGC) RNA-2/FRNA-2, one GGGGCC-repeated 8-mer CCGGGGCC RNA-3/FRNA-3, and different lengths of telomere RNA UUAGGG repeats (Tel-RNA). Two RNA r(GCGGCAGC) RNA-4/FRNA-4 and r(GC^F^GGCAGC) RNA-5/FRNA-5 with A replaced G were used as controls. Because biomolecules function in a crowded intracellular environment, we employed a widely used cosolute, PEG 200, to mimic the molecular crowding in a biological system in vitro^5,40,41^. Three short RNA sequences FRNA-1, FRNA-2, and FRNA-3 exhibiting high GC content aggregated into bulky clusters up to sizes of >100 µm with increasing PEG 200; the condensate was not observed with the control FRNA-4 and FRNA-5 (Fig. 1a). This is consistent with RNA self-assembly may be enhanced by the molecular crowding condition^42^, which has an effect on the self-association of assemblies^43^. Clusters were observable at RNA concentrations as low as 28 nM, and increased in size with increasing concentration (Supplementary Fig. 1). Shorter complementary antisense oligonucleotide (ASO) prevented clustering of FRNA-1 and FRNA-2 in 10% PEG, while control oligonucleotide did not (Fig. 1b), corresponding to the previous study that ASO may disrupt RNA foci by inhibiting intermolecular base-pairing^9^. However, ASO cannot disturb FRNA-1 and FRNA-2 clusters in 40% PEG (Fig. 1c), suggesting the intermolecular interactions between the RNAs cannot be competed out by ASO in a cell-like high crowding environment. The disease-related RNAs immediately formed a turbid solution after mixing RNA solution with 10% PEG and were precipitated via solidification using 40% PEG in a cell-like environment, whereas the control RNAs, telomere RNAs remained soluble (Supplementary Fig. 2). These experiments indicate that short RNA sequences can lead to the stabilized clusters and even entry into solid-like state in the crowded cellular environment.

**Fig. 2:**
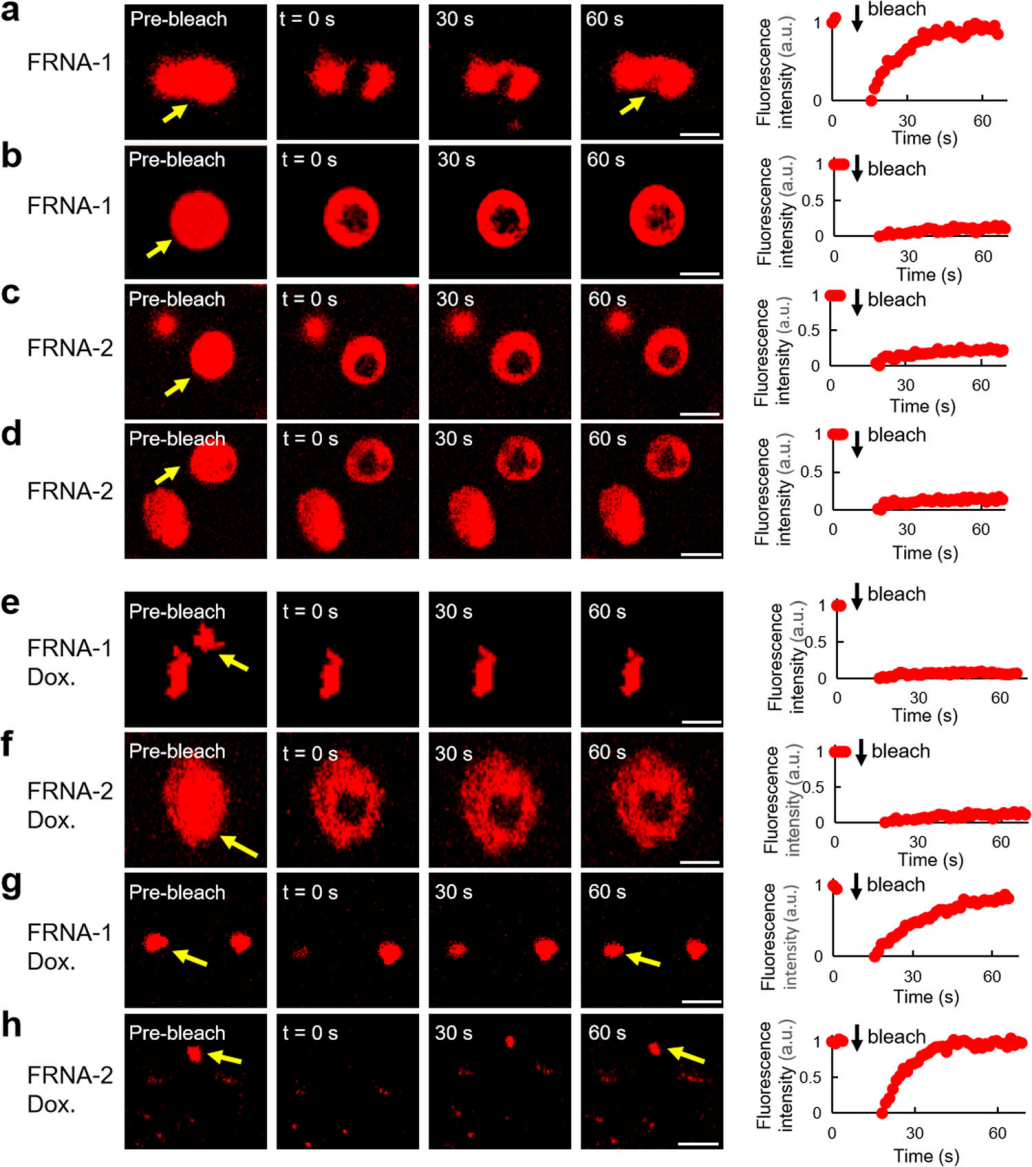
FRAP analysis of RNA aggregates. **a**, **b** Fluorescence recovery after photobleaching experiments for FRNA-1 before and after photobleaching (arrow, bleach site and cluster) at 10% **a** and 40% **b** PEG. Graphs represent recovery plot corresponding to (**a**, **b**). **c**, **d** Fluorescence recovery after photobleaching experiments for FRNA-2 before and after photobleaching (arrow, bleach cluster) at 10% **c** and 40% **d** PEG. Graphs represent recovery plot corresponding to (**c**, **d**). **e**, **f** Inhibitor doxorubicin in fluorescence recovery after photobleaching experiments for FRNA-1 and FRNA-2 before and after photobleaching (arrow, bleach cluster) at 40% PEG. Graphs represent recovery plot corresponding to (**e**, **f**). **g**, **h** Inhibitor doxorubicin in fluorescence recovery after photobleaching experiments for FRNA-1 and FRNA-2 before and after photobleaching (arrow, bleach cluster) at 10% PEG. Graphs represent recovery plot corresponding to (**g**, **h**). Scale bars, 5 µm.

Fluorescence recovery after photobleaching (FRAP) was used to investigate the RNAs properties by observing their relatively immobile physicochemical state following induction^9^. FRNA-1 showed fluorescence recovery in a low PEG concentration (10%) indicating that the RNAs self-assembled into mobile, no recovery in a high PEG concentration (40%) (Fig. 2a, b and Supplementary Movie 1, 2). FRNA-2 in both 10% and 40% PEG is unable to recovery, suggesting that the RNA clusters are immobile (Fig. 2c, d and Supplementary Movie 3, 4). FRNA-3 exhibited fluorescence recovery in 40% PEG (Supplementary Fig. 3a). These observations are consistent with the findings of previous studies that have reported that RNA self-assembly is primarily affected by the RNA structure and crowded cellular environment^5,42,43^.

Next, we employed two well-known phase transition inhibitors, doxorubicin and 1,6-hexanediol, which disrupt interactions between biocondensates, to investigate RNA aggregation^9,44,45^. Treatment with either of the two inhibitors in 40% PEG, no fluorescence recovery was observed (Fig. 2e, f, Supplementary Fig. 3b, c and Supplementary Movie 5–8), suggesting short RNAs preferred to solidify in a cell-like high crowding environment, referring to as a ‘solid-like state’. FRNA-1 and FRNA-2 showed fluorescence recovery in a low PEG concentration (10%) (Fig. 2g, h, Supplementary Fig. 3d, e and Supplementary Movie 9–12). We noted that FRNA-1 showed a similar behavior with no treatment condition but different in FRNA-2. This suggested that the FRNA-2 aggregation was disrupted into weak interactions in a low PEG concentration (10% PEG), whereas the solid-like state in 40% PEG remained unaffected. Furthermore, time-dependence experiments revealed that RNA clusters disappeared in 10% PEG for an extended period, and there was almost no change in 40% PEG (Supplementary Fig. 3f–i). These results indicate that the inhibitors blocked RNA condensate formation and potently dissolved the RNA clusters under low crowding condition. However, they were unable to effectively break the solid RNA in a cell-like high crowding environment.

We further investigated the physical properties of RNA during phase transition using UV-visible spectroscopy. Absorbance of both RNAs dramatically decreased with increasing PEG concentration, but not of control RNA (Supplementary Fig. 4a, b), indicating that RNA aggregation depletes the dissolved RNA in solution in concordance with the above results. Subsequently, we examined the effect of metal ions on RNA aggregation, which are known to be key in higher-order RNA structure formation^46^. Moreover, absorbance declined with increasing K^+^ concentrations but that of control RNA remained the same (Supplementary Fig. 4c, d). Accordingly, we observed no RNA aggregation without K^+^ (Supplementary Fig. 4e). These results indicate that RNA phase transition is not only affected by surrounding environment but is also governed by the higher ordered RNA structures. The clusters were dissolved by RNaseA digestion in 10% PEG, but not in 40% PEG (Supplementary Fig. 4f, g), confirming that the RNAs aggregated into solid-like state in 40% PEG a cell-like environment and induced a strong RNase resistance, which is consistent with the above results.

### NMR reveals agglutinated RNA structures formed by short RNAs

**Fig. 3:**
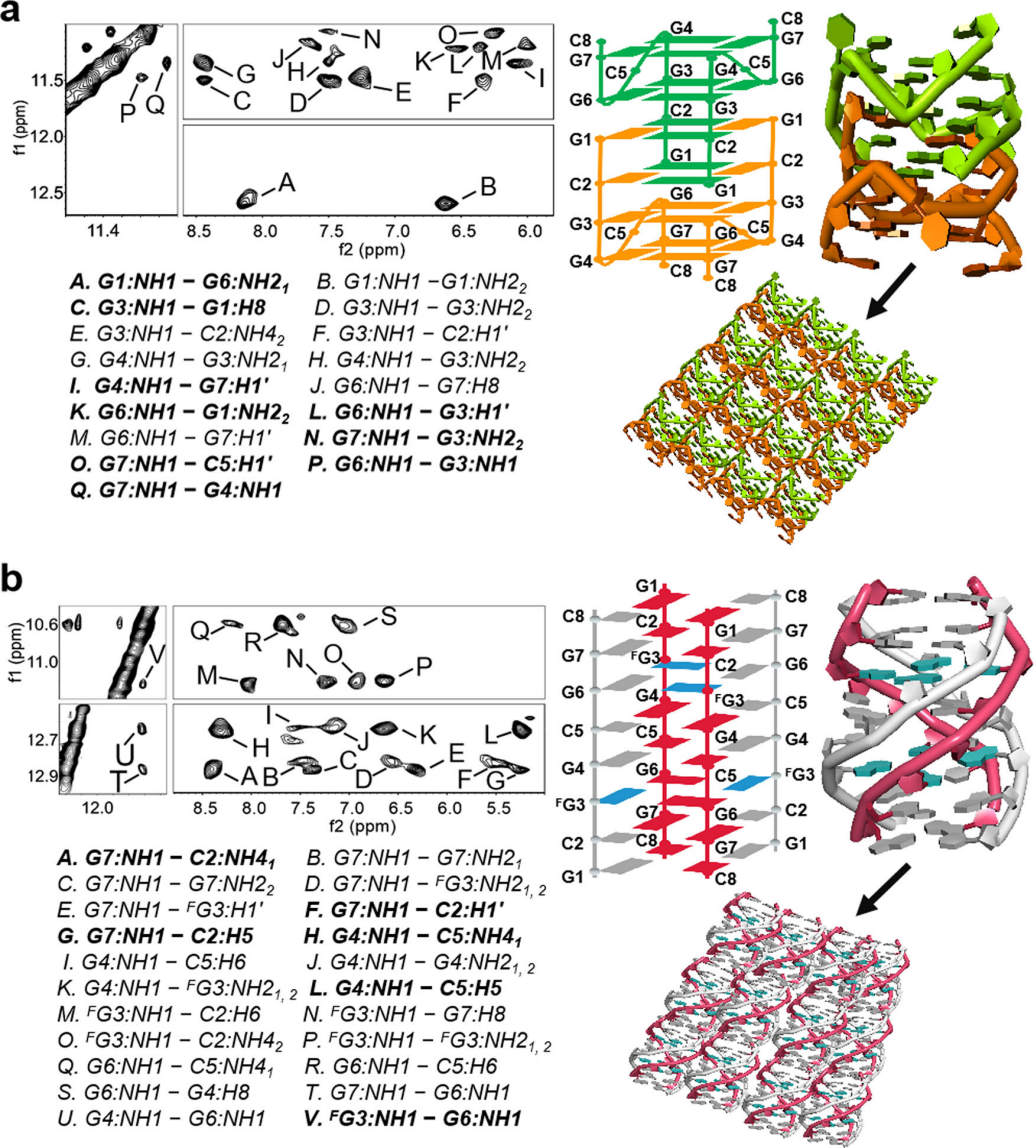
NMR reveals RNA-1 and RNA-2 structures. **a** Imino-imino, imino-amino, imino-H8 (G), and imino-H1′ regions of 2D NOESY spectra of RNA-1. The cross peaks A to Q are assigned. Cross peaks for P (G6:NH1 and G3:NH1), Q (G7:NH1 and G4:NH1), L (G6:NH1 and G3:H1′), and I (G4:NH1 and G7:H1′) are shown in bold for Gtetrads G3:G6:G3:G6 and G4:G7:G4:G7. Other cross peaks of A (G1:NH1 and G6:NH2_1_), C (G3:NH1 and G1:H8), K (G6:NH1 and G1:NH2_2_), O (G7:NH1 and C5:H1′), and N (G7:NH1 and G3:NH2_2_) are shown in bold also. Schematic structure of RNA-1. For clarity, bases from the symmetric G-quadruplex are colored in green and orange. The residue numbers are illustrated. Ribbon view of the G-quadruplex structure with green and orange colors and aggregation by self-assembled G-quadruplex. **b** Imino-imino, imino-amino, imino-H8 (G), and imino-H1′ regions of 2D NOESY spectra of RNA-2. The cross peaks A to V are assigned. Cross peaks for V (^F^G3:NH1 and G6:NH1), A (G7:NH1 and C2:NH4_1_), F (G7:NH1 and C2:H1′), H (G4:NH1 and C5:NH4_1_), L (G4:NH1 and C5:H5), and G (G7:NH1 and C2:H5) are shown in bold for G-tetrads ^F^G3:G6:^F^G3:G6 and base pairs C2:G7 and G4:C5. Schematic structure of RNA-2. Bases from the G-quadruplex are colored in red, silver and blue. The residue numbers are illustrated. Ribbon view of the G-quadruplex structure with red and silver colors and aggregation by self-assembled G-quadruplex.

The above results prompted us to investigate the molecular characteristics of these short RNAs. In the imino proton region of the ^1^H NMR spectrum of RNA-1 (Supplementary Fig. 5a), in the presence of K^+^, four sharp peaks at 11.0–11.5 ppm were observed, reflecting the Hoogesteen G:G base pairs assigned to the region distinct to G-quadruplex structure. One guanosine imino proton resonated at 12.55 ppm, which is typical of Watson–Crick G-C base pairs. The sequential NOE interactions of H6*/*H8-H1′ NOE connectivity could be traced in the 2D NOESY spectra (Supplementary Fig. 5b). Two G-tetrads comprising G3:G6:G3:G6 and G4:G7:G4:G7 were observed and confirmed by the NOE imino interaction peaks of G6:NH1 and G3:NH1, G7:NH1 and G4:NH1, G6:NH1 and G3:H1′, and G4:NH1 and G7:H1′ by the formation of G3:G6 and G4:G7 Hoogesteen base pairs (Fig. 3a). The amino–amino NOE peaks of C2: NH4_1_ and G1:NH2_2_ and the NOE peaks between G1:H8 and C2:H5 were observed (Supplementary Fig. 5b), which enabled the unambiguous identification of G1:C2 Watson–Crick base pairs. These results indicate the formation of a symmetric tetramolecular G-quadruplex structure with G:G:G:G and mixed G:C:G:C tetrads (Fig. 3a and Supplementary Fig. 5c). Furthermore, theresults were consistent with the observation of NOE interactions of G1:NH1 and G6:NH2, G3:NH1and G1:H8, G6:NH1 and G1:NH2, and G7:NH1 and G3:NH2, as well as G7:NH1 and and C5:H1′ (Fig. 3a). The self-assembly of G-quadruplex will promote RNA-RNA interactions to form aggregates.

Next, we uncovered the structural features of RNA-2, wherein an 8-trifluoromethyl-guanosine (^F^G) nucleoside acts as a ^19^F sensor and can be employed to study the RNA structure in vitro and in cells using ^19^F NMR. We successfully synthesized ^F^G and incorporated it into RNA sequences (Supplementary Figs. 10~42). The imino region of the ^1^H NMR spectrum showed that the presence of one set of imino proton signals represent the formation of one major conformation. The guanosine imino protons in ^1^H NMR observed in the range of 12.0–13.0 ppm are typical of Watson–Crick base pairs and those between 10.2 and 11.5 ppm are characteristic of Hoogesteen G:G base pairs (Supplementary Fig. 5d), indicating a G-quadruplex structure exhibiting mixed G:C Watson–Crick pairs. The 2D NOESY demonstrated a tetramolecular G-quadruplex structure with two ^F^G3:G6:^F^G3:G6 tetrads and six G:C base pairs (Fig. 3b). These cross peaks involved the imino-imino interaction of ^F^G3-NH1 and G6-NH1 and NOE contacts of G4:NH1 and C5:NH4_1_, G7:NH1 and C2:NH4_1_, G7:NH1 and C2:H5 from G4:C5 and G7:C2. The cross peaks observed between the H8 proton of G1 and the amino proton of C8 suggested the composition of the G1:C8 base pair (Supplementary Fig. 5e). The continuous set of sequential H6*/*H8-H1′ NOE connectivity can be traced with a break appearing between C2 and ^F^G3 step owing to the absence of a proton at the C8 position of the ^F^G3 (Supplementary Fig. 5e). These results consistent with previous studies reporting a brominated RNA containing 8-bromo guanosine that adopted a tetramolecular G-quadruplex structure (Fig. 3b and Supplementary Fig. 5f)^37^. Similar to RNA-1, the aggregation of RNA-2 will be promoted by self-assembled G-quadruplex.

Conversely, the imino proton spectrum of control RNA exhibited only one resonance at 12.45 ppm as a typical G:C base pair and was identical with the proton peak of G4-NH1 (Supplementary Fig. 5g), thereby suggesting the presence of duplex conformation mixed with single strand. Gel electrophoresis analysis revealed that FRNA-2 exhibited slower mobility compared with control FRNA-5 and confirmed that FRNA-2 possesses a higher ordered topology (Supplementary Fig. 5h). According to CD melting experiments, the *Tm* values of the two G-quadruplexes in 150 mM KCl solution were above 50 °C, suggesting that two RNAs form the stable G-quadurplexes (Supplementary Fig. 5i).

Recently, we demonstrated that fluorinated RNAs can be used to directly observe higher-order RNA structures in vitro and in cells using ^19^F NMR^47,48^. The advantages of ^19^F NMR are the high sensitivity of the ^19^F chemical shift to the environment, absence of any natural background signals in RNA and cells, and relative simplicity of ^19^F NMR spectra^47–49^. Next, we used ^19^F NMR spectroscopy to investigate the structural features of the fluorinated RNAs. In the diluted solution, one peak was observed at −61.29 ppm (Supplementary Fig. 5j), indicating a G-quadruplex formation, which is consistent with the above results. As the PEG concentration increased, the ^19^F signal broadened and attenuated, indicating that the self-assembly of the RNAs into aggregates, which is consistent with the mechanism of ^19^F NMR chemical shift, as demonstrated by previous studies stating that the formation of a large molecular assembly can cause severe broadening of the signal in ^19^F NMR spectroscopy^48,50^. The signals in ^19^F NMR spectroscopy are extremely sensitive to the apparent Mr (aggregates of high molecular mass) because ^19^F has a relatively large chemical shift anisotropy (~39 ppm for a CF_3_ group)^48,51^. Conversely, two peaks of control were observed in the presence of KCl diluted solution, indicating a duplex and single strand RNA, consistent with the ^1^H NMR result (Supplementary Fig. 5k). When the PEG concentration was increased, the peaks for duplex disappeared and only a single strand was clearly observed, which is in accordance with previous studies reporting that a molecular crowding condition induces the destabilization of duplexes with Watson–Crick base pairs^52^. Additionally, CD spectrum also indicated the characteristic pattern of a G-quadruplex structure as a positive band at 270 nm when using RNA-1 and RNA-2 in KCl. This observation is consistent with previously reported CD shape of tetramolecular quadruplexes^37,38^. Notably, increasing PEG contents similarly stabilized the G-quadruplex structures, as evidenced by the heightened signals at 270 nm (with higher PEG concentrations inducing suspension) (Supplementary Fig. 6).

### Solid short RNA foci in cells and in vivo

**Fig. 4:**
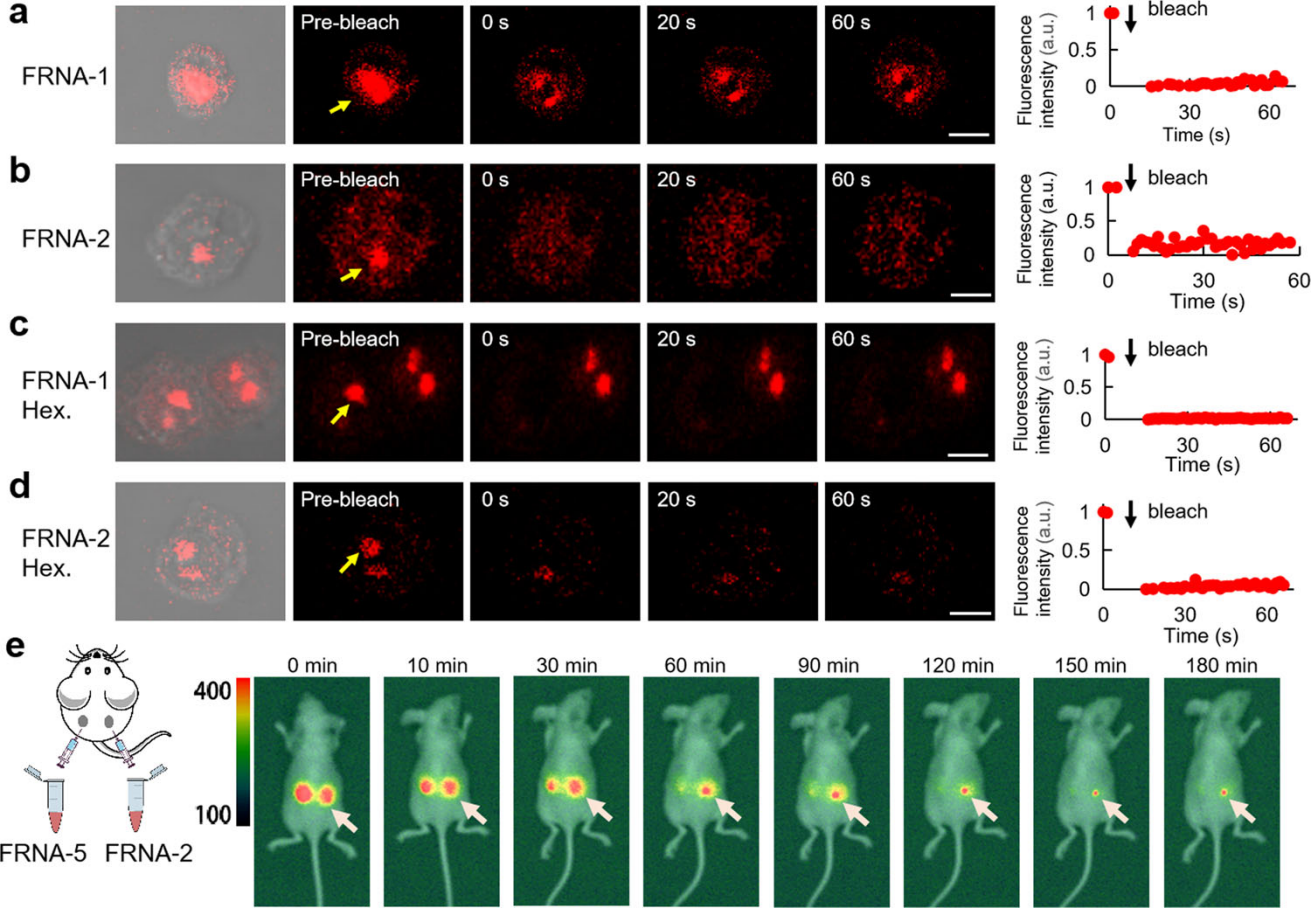
Short RNA foci in cells and in vivo. **a** FRNA-1 clusters localized in HeLa cells and analyzed by FRAP (arrow, bleach cluster). Graphs represent recovery plot corresponding to **a b**, RNA-2 clusters localized in HeLa cells and analyzed by FRAP (arrow, bleach cluster). Graphs represent recovery plot corresponding to **b c**, FRNA-1 clusters localized in HeLa cells treated with inhibitor 1,6-hexanediol and analyzed by FRAP (arrow, bleach cluster). Graphs represent recovery plot corresponding to **c d**, RNA-2 clusters localized in HeLa cells treated with inhibitor 1,6-hexanediol and analyzed by FRAP (arrow, bleach cluster). Graphs represent recovery plot corresponding to **d e**, RNA aggregation in living mice. Time-dependence of fluorescence imaging of RNA-2 (right) and FRNA-5 (left) after injection. Scale bars, 5 µm.

To gain an in-depth understanding of the physicochemical states of RNA within the physiological environment, we constructed the SLO system, which has been confirmed to be highly effective in promoting the entry of macromolecules, such as nucleic acids and proteins, into cells, thus introducing RNAs into HeLa cells^53,54^. Upon photobleaching a portion of FRNA-1 punctum, the fluorescence signal did not recover from the unbleached region to the bleached region (Fig. 4a and Supplementary Movie 13), suggesting that RNA within the foci cannot undergo internal rearrangement. Thus, similar to their solid-like behavior in vitro, short RNA foci in cells also displayed solid-like properties. Upon photobleaching, the foci of FRNA-2 also did not exhibit fluorescence recovery (Fig. 4b and Supplementary Movie 14), indicating that the RNA cannot move into and out of the foci, consistent with internal immobility. FRAP experiments showed no fluorescence restoration even after doxorubicin or 1,6-hexanediol treatment, which indicates solid-like RNA properties (Fig. 4c, d, Supplementary Fig. 7a, b and Supplementary Movie 15–18)^5,55^. RNA-driven condensed foci of the two RNAs were observed, and two or more foci could not fuse with one another for an extended period (Supplementary Fig. 7c, d), consistent with the hallmark of solid like behavior. Furthermore, there was almost no change for FRNA-1 and FRNA-2 clusters in treatment with either of the two inhibitors (Supplementary Fig. 7e, f). Conversely, in comparison to the two RNAs, the aggregations in the control FRNA-4 and FRNA-5 samples were not observed (Supplementary Fig. 7g, h).

The aggregation of shorter RNAs into solid foci in cells led us to explore whether they form aberrant foci in vivo. Nude mice with robust metabolic systems including nucleases were used in this study. The two RNA species in solution were injected on either side of the back of the mice to observe the change in fluorescence signals over time (Fig. 4e). FRNA-2 was stably present alongside aggregation even after 3 h; however, control FRNA-5 was rapidly metabolized after 1 h, as visualized by fluorescence imaging. These results demonstrate that short RNA can strongly aggregate and was poorly metabolized by nucleases in vivo and indicate that short CGG-repeat RNA accompanied by strong aggregation can contribute to the pathogenesis of neurological diseases caused by accumulated RNA foci.

We further used ^19^F NMR to investigate the RNA aggregations in cells. The RNA-2 and control RNA-5 were examined to make a comparison of the in vitro and in-cell NMR spectra, respectively (Supplementary Fig. 8). In cells, only one signal was observed in the ^19^F NMR spectrum of control RNA, which are nearly identical to its observed in diluted solution and 40% PEG, indicating the single strand RNA in living human cells. Conversely, RNA-2 showed the broadened and attenuated ^19^F signal in cells, indicating that the self-assembly of the RNAs into aggregates, which is consistent with the above results.

### Short RNA foci induces cell dysfunction

**Fig. 5:**
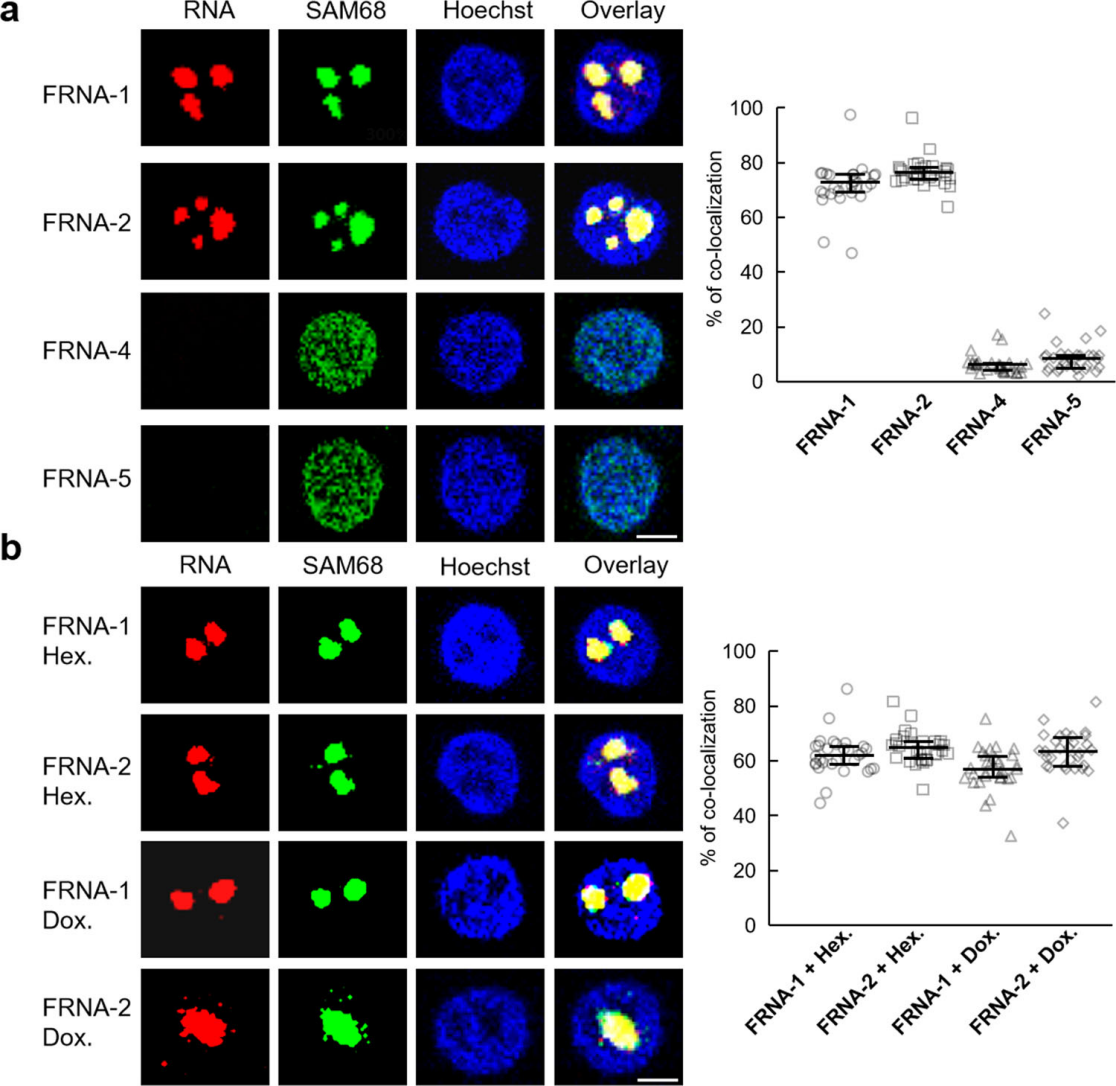
Short RNA foci disturb cell function. **a** Sam68 colocalizes with FRNA-1, FRNA-2 and control FRNA-4, FRNA-5 aggregates. Sam68 was immunostained using anti Sam68 antibodies (green). Blue fluorescence indicated nuclei with Hoechst. Red color is from RNAs. The overlay panel shows a colocalization event. The percentage of endogenous Sam68 colocalized within FRNA-1, FRNA-2 and control FRNA-4, FRNA-5 (*n* = 30). **b** Sam68 colocalizes with FRNA-1 and FRNA-2 aggregates in cells treated with inhibitor doxorubicin or 1,6-hexanediol. Data are representative of at least three independent experiments (*n* = 30) and results are presented as mean ± interquartile. Scale bars, 5 µm.

The aggregation of short RNAs into solid foci prompted us to explore their effect on cellular functions. Reportedly, Sam68, an RNA binding protein involved in alternative splicing regulation, in patients with fragile X-associated tremor/ataxia syndrome loses its splicing regulatory function and induces altered mRNA processing by sequestrating and colocalizing with CGG repeats^13,56^. Similar to the results of endogenous Sam68 colocalized with longer CGG repeats aggregates in COS7 cells, we found that ~70% of either FRNA-1 or FRNA-2 colocalized with Sam68 in the nucleus compared with <10% of control FRNA-4 and FRNA-5 (Fig. 5a). Notably, RNA induced colocalization persisted even after the addition of the inhibitors 1,6-hexanediol and doxorubicin (Fig. 5b), and this result was consistent with the solid RNA foci not being affected by these molecular aggregation antagonists. Thus, such RNA foci from short CGG repeats retained the RNA in cells as a sequestrator of splicing factors.

**Fig. 6:**
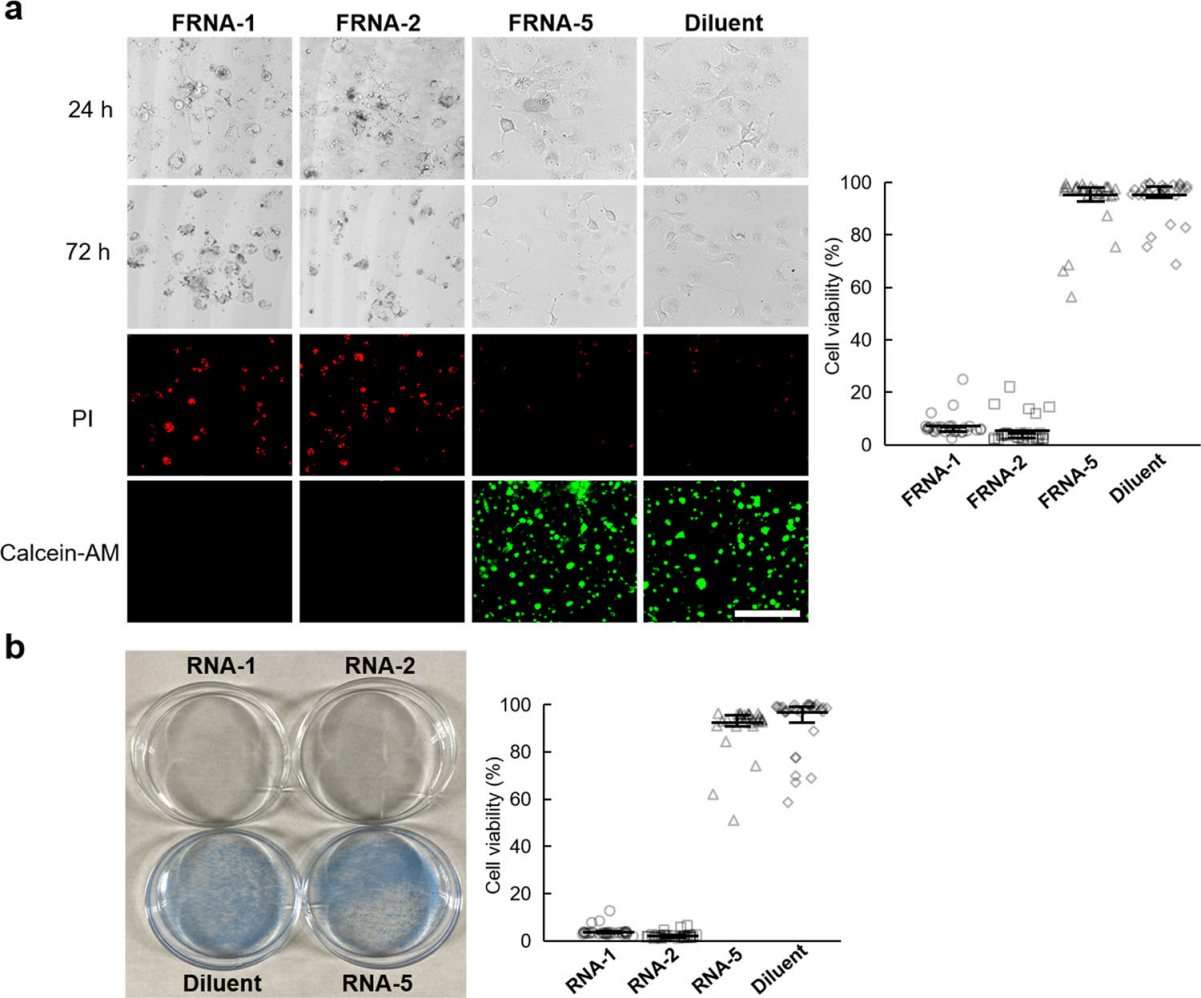
Short RNA foci induce cell death. **a** Morphologic changes of Hela cells during the treatment of RNAs and diluent. The resulting cells were incubated and imaged at 24 and 72 h. Dead and live cells at 72 h were stained with a nuclear counterstain and visualized as fluorescence. Dead cells were stained with PI and fluoresced red; live cells were stained with Calcein-AM and fluoresced green (*n* = 30). **b** Cells were treated with RNAs and diluent. The cells were then incubated and assayed (clonogenic assay). Colonies of cells were stained with methylene blue. Quantification of clonogenic capacity. Colonies in triplicate cultures, shown above, were counted and plotted as a percentage of the diluent-treated control (*n* = 30). Error bars represent mean ± interquartile. Scale bars, 250 µm.

To further explore the effect of RNA foci on cell toxicity, COS7 cells were incubated with short RNAs and imaged at 24 and 72 h (Fig. 6a). Treatments with RNAs showed considerable morphological changes after 24 h, with more pronounced shrinkage appearing at 72 h. Conversely, neither FRNA-5 nor the diluent induced observable morphological changes. The live and dead cells at 72 h were stained and visualized using a nuclear counterstain to examine the toxicity of the short RNAs. Intense red fluorescence from the dead cells was evident compared with green fluorescence from the live cells treated with control RNA or buffer, revealing that the short CGG repeats caused cell death, and ~95% of the cells were confirmed to be nonviable based on the percentage calculation of red fluorescence in the total fluorescence range. A clonogenic assay was employed to examine the clonogenic capacity of the short RNA-treated cells (Fig. 6b). COS7 cells were treated with short RNAs and diluent for 72 h and then stained with methylene blue for live cell imaging. The clonogenicity of the cells pretreated with either of the short RNAs was almost completely suppressed; conversely, the cells treated with the control and diluent alone showed a substantial increase in their clonogenic capacity. These results suggest that the RNA foci with short CGG repeats can disrupt functional cellular processes, eventually inducing cell death^57–59^.

## Discussion

In summary, we showed that the propensity of short RNAs to form G-quadruplex structures can induce aggregation into solid-like states. Recently, numerous studies have characterized long poly-RNAs and ribonucleoproteins owing to their ability to induce phase transitions and mediate RNA foci formation^60^. Our results demonstrate that the structure-specific properties of short RNAs can induce their liquid-to-solid phase transition in vitro, which can even be observed in vivo. These RNA foci induce the colocalization of RNA binding proteins and suppress the clonogenicity of cells, eventually induce cell death^11,12^.

The self-assembly of RNA by forming hydrogen bonds between bases promote both intramolecular and intermolecular RNA-RNA interactions^5,61^. G-quadruplex structures are comprised of several planar layers using G-tetrads^27,62^. The self-assembled G-quadruplex via end-to-end stacking into rod-shaped aggregates forms aggregates and induces the phase transition of RNA, thereby raising the possibility that such phenomena could contribute to RNA foci formation. Previous studies have suggested that small double-stranded DNA and RNA prefer to stack end-to-end while repelling sideways, causing the self-organization of motifs through liquid crystallization^63–66^. The self-assembly of DNA or RNA is a spontaneous process driven by free energy minimization, which arises from end-to-end adhesion with the stacking of bases and the weak electrostatic attractive interaction along the sides between the negative charges of the backbone phosphates of DNA or RNA oligomers^64,67,68^. In this manner, G-quadruplex RNA is most likely to stack via the strong end-to-end attraction of the basic G-tetrad units because of the wider surface to stack compared with duplex^69^. Specifically, the formation of G:C base pairs at the end of each strand is particularly favored at promoting end-to-end attraction. In addition, we and other groups have reported that RNA can form polymorphic higher-order G-quadruplexes comprising stacked G-quadruplex subunits^33,47^. These results unanimously demonstrate that short nucleotides gather and trigger phase behaviors via intermolecular organization in agreement with biological activity demands.

Previous studies have demonstrated that the molecular crowding condition stabilizes the G-quadruplex conformation^41,47^. RNA self-assembly is enhanced in the crowded cellular environment, which exhibits a greater effect on the self-association of molecules or assemblies^5^. It is believed that molecular crowding demonstrates a greater effect on the effective concentration of molecules where the availability of solvent is much more reduced^5,41^. These observations suggest that high concentration of exposed RNA induce intermolecular RNA G-quadruplex interactions, which contribute to higher-order RNA assemblies^5,47^. Collectively, G-quadruplex structure and crowding environment both are benefits and indispensable in the promotion of short RNA phase transition, in which particular architecture of G-quadruplex with numerous effective sites favoring interplay among assemblies, in more stable and higher concentrations pattern under crowding environment, dramatically contribute supramolecular structure formation via intermolecular G-quadruplex interaction.

Despite the fact that telomeric RNA sequences can adopt a stable G-quadruplex conformation, the lack of cytosine in telomeric sequences appears to be unfavorable for promoting end-to-end attraction. According to a previous report, the regular appearance of three hydrogen bonds in favored G:C base pairs is crucial for linking subunits or assemblies together^9,70^. Thus, G-rich sequences in the telomeric region may face challenges in forming supramolecular structures via intermolecular G-quadruplex interactions due to the absence of cytosine. It is hypothesized that expanded CGG DNA repeats containing G:C base pairs will have the ability to aggregate. This is consistent with previous study suggesting that the condensation of GGGGCC repeats in vitro is more prominent for longer repeats^23^.

Our data suggest that RNA foci formation in repeat expansion disease impedes cell growth and cause cell death, which is a clear example of the production of toxic RNA aggregates that have the potential for multiple intermolecular interactions and the formation of a cellular multimeric RNA assembly.

**Fig. 7:**
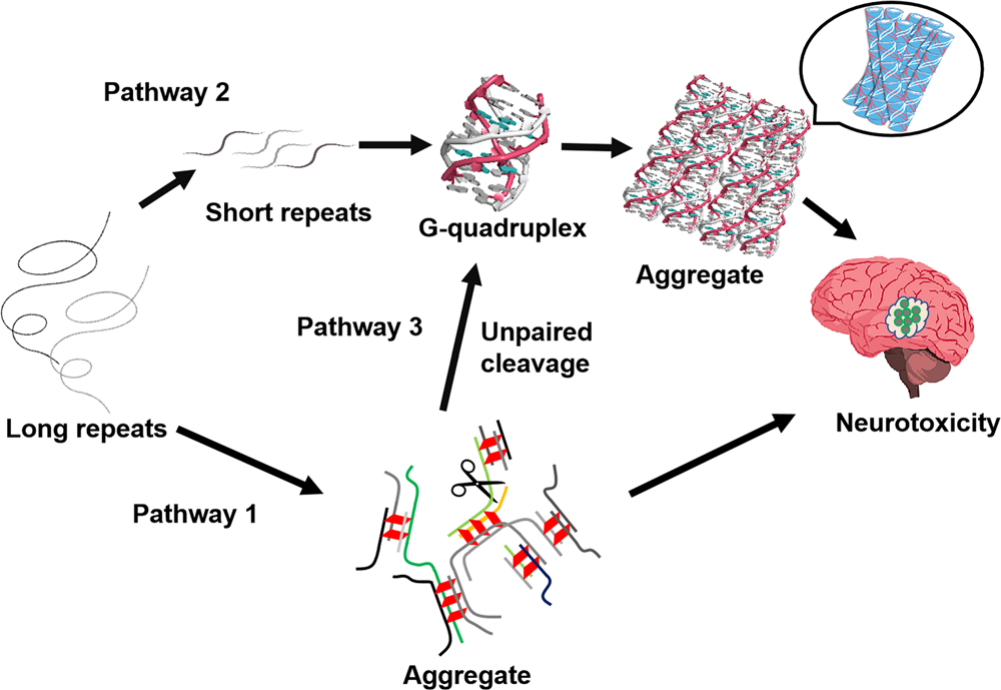
Schematic representation of pathways for RNA foci formation and neurotoxicity. In addition to pathway 1 of longer RNAs, pathway 2 and 3 lead to the formation of RNA foci in repeat expansion disorders. See text for details.

In the case of RNA foci in repeat expansion diseases, the simplest interpretation is that longer RNAs have more sites for multimolecular RNA aggregation through intermolecular RNA-RNA interactions, including RNA G-quadruplex, and thus show enhanced self-association both in cells and in vitro (pathway 1) (Fig. 7)^9^. Our findings suggest the alternative pathways of RNA aggregate foci-induced neurological diseases. Different RNases in cells cleave longer RNAs, producing a large amount of short RNAs to form RNA G-quadruplexes and further induce RNA aggregation (pathway 2). Alternatively, multimolecular RNA complexes formed via pathway 1 can be degraded at the unpaired regions outside of G-quadruplexes, producing numerous RNA G-quadruplex fragments that can accumulate as RNA foci (pathway 3). This is consistent with the findings of previous studies suggesting that longer RNAs having the CGG trinucleotide region undergo a faint digestion at the CGG trinucleotide sequences because of the secondary structure at the region^30^. Furthermore, previous studies have demonstrated that the telomere RNA G-quadruplex induces a strong RNase resistance in UUAGGG telomere RNA repeats^31,32^, and RNA G-quadruplex resisting telomere RNA degradation provides a concentration supply and longer lifetime of the telomere RNA to participate in essential biological processes^25^. This agrees with our observations that RNA clusters in a cell-like environment were not dissolved by RNaseA and induced a strong RNase resistance, similarly to the result that the RNA foci cannot be disturbed by ASO and agents.

The finding that short RNAs form RNA foci and is promoted by RNA G-quadruplex structures opens new doors to better understand the essential biological role of RNA aggregation in neurodegenerative diseases. This increases the avenues of developing new approaches targeting RNA G-quadruplex structures to treat repeat expansion diseases.

## Methods

### RNA sample preparation

By using an automatic solid-phase phosphoramidite chemistry and DNA/RNA synthesizer, designed RNAs were synthesized at a ratio of 1.0 µmol. We synthesized RNA sequences (Supplementary Figs. 10~42). Following the RNAs were cleavage from the column and deprotected by using AMA (Ammonium Hydroxide*/*40% aqueous methylamine 1:1 v*/*v) at room temperature for 20 min and at 65 °C for 10 min, respectively. TBDMS protections were removed by treatment with triethylamine trihydrofluoride followed by filtration through an ion exchange cartridge. The oligomers were further purified by HPLC in a linear gradient of 50 mM ammonium formate in 1:1 acetonitrile*/*H_2_O and 50 mM ammonium formate in H_2_O. The oligomers were desalted through a NAP 10 column (GE Healthcare) and identified by MALDI-TOF-MS on an Autoflex III smart beam mass spectrometer (negative mode). The all RNA sequences used in this study shown in Supplementary Table 1.

### RNA phase separation, gelation and solidification

For phase separation or transition experiments, RNAs or Cy3 labeled RNAs were diluted to 0.01-0.3 mM in 150 mM KCl, 25 mM MgCl_2_, 10 mM Tris-HCl buffer (pH 7.0), unless indicated otherwise. RNA was denatured at 85 °C for 3 min in a thermocycler and cooled down in ice to room temperatures, and PEG 200 was added and kept for another 5 min, then imaged immediately. Samples were visualized using TCS SP8 confocal microscopy (Leicamicrosystems). The data were recorded using Leica software. The laser of TCS SP8 for 63× was HC PL APO CS2 63x/1.40 OIL. The red fluorescence for Cy3 dye was an excitation wavelength of 550 nm. The excitation/emission filter of 520−550 nm/550−600 nm was used for RNA samples. Max intensity z-projection and a single 0.5-µm optical slice was used for all images. For the experiments with aggregated disruption reagent treated RNA samples, determined concentrations of doxorubicin or 1,6-hexanediol/digitonion were added into RNA condensates after RNA aggregation occurred for 20 min, then the fluorescence imaging was monitored over time.

### FRAP assay in vitro

RNA clusters were prepared as described above. RNA clusters were allowed to settle on to the glass surface for ~5 min. A region of ~1.5 µm^2^ was photobleached using a 488 nm laser with 100% power using bleaching program of the Leica software on TCS SP8 in 10 s and the fluorescence recovery process was monitored by time-lapse imaging within 60 s. For the aggregated disruption reagents treated RNA samples, FRAP was introduced after connective imaging of RNA clusters for 90 min. The fluorescence intensity of the bleached region was normalized and corrected for photobleaching. To determine fluorescence relaxation time, the recovery curves were fitted to the equation *I* = *A* − *I*_0_ exp(−t/*τ*_FRAP_), where *A* and *I*_0_ are also fit parameters. Time-lapse images were recorded per second using HC PL APO CS2 63×/1.40 OIL for tracking photo recovery behavior.

### Cell culture and imaging

HeLa cells (CCL2) grown in Dulbecco’s modified Eagle’s medium (DMEM) medium containing 10% FBS under a 5% CO_2_ atmosphere were harvested (2 × 10^5^) and washed twice by using Hanks’ Balanced Salt Solution (HBSS) buffer. Streptolysin O (Bioacademia) was activated with 10 mM DTT and 0.05% bovine serum albumin at 37 °C for 2 h. To form pores on plasma membranes of HeLa cells, the activity SLO (streptolysin O) was added for getting a final concentration of 0.1 µg mL^−1^. Then, the HeLa cells were incubated at 4 °C for 15 min with gentle rotation. The cells were washed three times with ice-cold HBSS buffer, followed by incubation with 5 µM RNAs in 400 µL HBSS buffer at 37 °C for 30 min. For resealing of the HeLa cells membranes, CaCl_2_ was added to a final concentration of 1 mM and the HeLa cells were incubated at 37 °C for 30 min. The HeLa cells were washed three times with HBSS buffer containing 1 mM CaCl_2_. The resealed HeLa cells were added by 100 µL DMEM medium and directly imaging.

For RNA aggregation disruption assays, doxorubicin (stock, 10 mM in DMSO) was diluted to 2 µM in cell culture medium and added to cells pre-treated with SLO system for 24 h. Cells were incubated with an equivalent dilution of DMSO only as control, for 24 h, and imaged as described above. Imaging for connective 90 min and no cells dying was observed. 1,6-hexanediol was diluted to 10% concentration with 10 µg mL^−1^ digitonion in the cells pre-treated with SLO system for 30 min. Imaging for sequential 90 min and no cells dying was observed. The cells were visualized using BZ-9000 fluorescence microscopy (KEYENCE) or TCS SP8 confocal microscopy (Leicamicrosystems). The data were recorded using Leica software. The laser of BZ-9000 for 20× was CFI Plan Fluor ELWD DM20×C; the laser of TCS SP8 for 63× was HC PL APO CS2 63x/1.40 OIL. The red fluorescence for Cy3 dye was an excitation wavelength of 550 nm. The excitation/emission filter of 520−550 nm/550−600 nm was used for RNA samples.

### FRAP assay in cells

To depict accurately the physiochemical dynamicity of RNA aggregation in cells, we performed fluorescence recovery after photobleaching (FRAP) assay. An area of ~1.5 µm^2^ was photobleached upon fluorescence region using a 488 nm laser with 100% power using bleaching program of the Leica software on TCS SP8 in 10 s and the fluorescence recovery process was monitored by time-lapse imaging within 60 s. For inhibition assay in living cell, FRAP was established after connective cellular imaging for 90 min by following above approach. The fluorescence intensity of the bleached region was normalized and corrected for photobleaching. To determine fluorescence relaxation time, the recovery curves were fitted to the equation *I* = *A* − *I*_0_ exp(−t/*τ*_FRAP_), where *A* and *I*0 are also fit parameters. Time lapse images were recorded per second using HC PL APO CS2 63×/1.40 OIL for tracking photo recovery behavior.

### ASO effect on RNA phase transition

RNAs (0.1 mM) in 150 mM KCl, and 10 mM Tris-HCl buffer (pH 7.0) was denatured at 85 °C for 3 min in a thermocycler and cooled down in ice to room temperatures, and PEG 200 was added and kept for another 3 min. ASO RNA CGCCGCCG (0.5 mM) and a control sequence GCGGCAGC (0.5 mM) were added and incubated for 30 min. The acquired sample directly used to fluorescence imaging assay.

### RNaseA digestion

RNAs (0.1 mM) in 150 mM KCl and 10 mM Tris-HCl buffer (pH 7.0) was denatured at 85 °C for 3 min in a thermocycler and cooled down in ice to room temperatures, and PEG 200 was added and kept for another 3 min. RNaseA (0.1 U mL^−1^) was added and incubated for 30 min. The acquired sample directly used to fluorescence imaging assay.

### RNA aggregates immunofluorescence assay

These RNAs and control sequences were transfected into live HeLa cells (2 × 10^4^) by transportation with Lipofectamine 3000 (*Thermo Fisher Scientific)* according to the manufacturer’s protocol in a final concentration of 10 µM. The treated cells were washed by PBS buffer and fixed with 4% paraformaldehyde. Thereafter, these cells were permeabilized with 0.2% Triton X-100 and stained using a fluorescent antibody, SAM68 (Santa Cruz, sc-514468, AF488) in a dilution of 1:400. After labeling, these cells were nuclear stained with 5 µg mL^−1^ Hoechst and imaged using TCS SP8 confocal microscopy (Leica microsystems). Afterward, if necessary, the intracellular RNA aggregates disruption assay was constructed by introduction of 10% hexanediol and 10 µg mL^−1^ digitonion or 2 µM doxorubicin (stock, 10 mM in DMSO) in live cells followed by immunofluorescence imaging.

### RNA aggregates give rise to cell toxicity

In this study, COS7 cells (CV-1, GIBCO^TM^) grown in Dulbecco’s modified Eagle’s medium (DMEM) medium containing 10% FBS under a 5% CO_2_ atmosphere were harvested (2 × 10^4^) and washed twice by using Hanks’ Balanced Salt Solution (HBSS) buffer before use. The RNAs were transfected into live COS7 cells by transportation with Lipofectamine 3000 (*Thermo Fisher Scientific)* according to the manufacturer’s protocol in a final concentration of 10 µM. After that, the cells were imaged in bright-field after 16, 24, 48, and 72 h. After incubation for 72 h, staining of nuclear DNA in dead and live cells was carried out using PI (Dojindo) and calcein-AM (Dojindo). The cells were incubated with PI or calcein AM in PBS (1.0 µM) for 20 min at 37 °C. The excitation and the absorbance filter was 550/12 nm and 600/40 nm for PI, or 488/40 and 530/30 for calcein-AM. Cell viability study was represented by the cells with green fluorescence (normalized by the green fluorescence of calcein staining from the PBS treated cells sample). In each group experiments, 3 independent experiments were completed and >10 images obtained in each result. At least 5 images that were randomly selected from the totally obtained confocal images >10 pieces containing intact cells, in which the representative images was divided into at least 4 subunits with equal size and the most representative one finally defined as presentation.

### Effect of RNA aggregates on clonogenic capacity of COS7 cells

The COS7 cells (2 × 10^4^) were cultured as description above. The RNAs were transfected into living COS7 cells by transportation with Lipofectamine 3000 (*Thermo Fisher Scientific)* according to the manufacturer’s protocol in a final concentration of 10 µM. The cells then were imaged in bright-field after 16, 24, 48, and 72 h. Finally, the cell colonies were stained for 20 min in 4% (w/v) methylene blue solution in PBS and washed. Colonies were counted and plotted as percentage of diluent-treated control. In each group experiments, 3 independent experiments were completed and 3 images obtained in each result, 1 representative image that was randomly selected from the totally obtained 3 confocal images and defined as presentation.

### RNA aggregation in vivo

The animal protocol was reviewed and approved by the Institutional Animal Care and Use Committee of University of Miyazaki. BALB/c nude mice (aged 6 weeks) were purchased from Charles River Laboratories Japan (Yokohama, Japan). Mice were injected a solution of FRNA-2 in saline or a solution of FRNA-5 in saline. Fluorescence imaging was performed using an in vivo imaging system (Lumazone CMS, Shoshin EM, Japan) at 3 h post injection. The excitation/emission filter of 520–550 nm/550–580 nm was used. Fluorescence intensity was quantified using the SlideBook 6 imaging software (Intelligent Imaging Innovations, Denver, CO, U.S.A.). All images were normalized by dividing the fluorescence image by a reference illumination image.

### Ultraviolet assay of RNA phase transition

The 10 µM RNAs solution containing 10 mM Tris-HCl buffer (pH 7.0) with different concentrations of KCl or PEG 200 were measured at absorbance of 550 nm (wavelength of Cy3-absorption). Before each sample was measured, the suspended solids were precipitated by centrifugation at 4,000 *g* for 10 min at room temperature. The concentration of the soluble RNA after centrifugation was determined by measuring absorbance at 550 nm.

### CD melting spectrum

CD experiments were performed by using a JASCO model J-820 CD spectrophotometer (JASCO Corporation, Tokyo, Japan). Condition: 20 µM RNA in 10 mM Tris-HCl pH 7.0 at 10°C. The melting curves were obtained by monitoring at 270 nm, 10-20 µM RNA in 0.3 mL samples containing 150 mM KCl and 10 mM Tris-HCl buffer (pH 7.0).

### ^19^F NMR spectroscopy

For ^19^F NMR measurement in vitro, RNA samples of 0.1 mM concentration were dissolved in 150 µl of designed solution containing 10% D_2_O, 150 mM KCl and 10 mM Tris-HCl buffer (pH 7.0). Samples were prepared by heating the ^19^F-labeled oligonucleotides at 85 °C for 3 min and gradually cooling to room temperature. The ^19^F NMR spectrum was measured on a Bruker AVANCE 400 MHz spectrometer at a frequency of 376.05 MHz and referenced to the internal standard CF_3_COOH (–75.66 ppm). The experimental parameters are recorded as follows: spectral width 89.3 kHz, ^19^F excitation pulse 15.0 µs, relaxation delay 1.5 s, acquisition time 0.73 s, scan numbers 256, and line width 3.For in-cell ^19^F NMR measurement, the 3 mM RNAs transfected cells were suspended in 200 µl of DMEM containing 10% D_2_O and transferred to a Shigemi tube (Shigemi 5 mm Symmetrical NMR microtube). The experiment was performed at 23 °C with a scan numbers value in 10^4^. After the intracellular NMR measurement, 100 µL of DMEM was added to the cell suspension, and the supernatant was collected by centrifugation at 400 × g for 3 min. The ^19^F NMR spectrum of the supernatant was measured with the same number of scans as the in-cell ^19^F NMR measurement. The remaining cells were mixed with 200 µl of DMEM containing 10% D_2_O and disrupted by ultrasound, the cell lysate was obtained and transferred to a Shigemi tube for performing ^19^F NMR spectrum with the same number of scans as the in-cell ^19^F NMR measurement.

### ^1^H NMR experiments

For spectra recorded in 90% H_2_O*/*10% D_2_O water signal was suppressed using the 3–9–19 WATERGATE pulse sequence or excitation sculpting with gradient pulse. The data were processed with TopSpin 3.0 (Bruker BioSpin Gmbh) software and analyzed with MestReNova software. For 1D NMR measurement, RNA samples of 2 mM concentration were dissolved in 150 µl of designed solution containing 10% D_2_O, 150 mM KCl and 10 mM Tris-HCl buffer (pH 7.0). The 2D NMR spectrum in 90% H_2_O*/*10% D_2_O was collected from 360 scans with 150 ms mixing time at 23 °C. On average, 2048 complex points and 512 FIDs were collected within the spectral width of 14097 Hz. The sample solutions were as follows: 3 mM RNA were dissolved in 150 µl of designed solution containing 10% D_2_O, 150 mM KCl and 10 mM Tris-HCl buffer (pH 7.0). Samples were prepared by heating the oligonucleotides at 85 °C for 3 min and gradually cooling to room temperature.

### Native gel electrophoresis

20 µM of FRNA-2 and FRNA-5 were dissolved in the 20 µL of solution containing 150 mM KCl, 25 mM MgCl_2_ and 10 mM Tris-HCl buffer (pH 7.0). Each sample was treated with heat denaturation, renaturation and was stored at 25 °C at least for 12 h. Then 3 µL of 30% glycerol was added before loading onto gel. Native gel electrophoresis (PAGE) was run on 20% polyacrylamide gel (acrylamide*/*bisacrylamide, 29:1) at 4 °C, in 1X TBE buffer.

### Molecular Modeling

We manually generated the model of RNA structure based on the reported structure (PDB code 3R1E) using the BIOVIA Discovery Studio 4.5. The molecular dynamics simulation was performed by the standard dynamics cascade in BIOVIA Discovery Studio 4.5 with some modifications. The structure was heated from 50 K to 283 K over 4 ps and equilibration at 283 K with 100 ps simulation time. The save results interval in the production step was 2 ps during 100 ps simulation time at 283 K. 10 best conformations generated by simulation were further energy minimized, and the conformation with lowest energy was selected.

### Statistics and Reproducibility

For the fluorescence image of studying RNA aggregation in vitro, in each group experiments, 3 independent experiments were completed and >20 images obtained in each result. At least 10 images that were randomly selected from the totally obtained confocal images >20 pieces, in which the most representative one was divided into at least 4 subunits with equal size and the most representative one finally defined as presentation. The extent of phase separation/transition was quantified by the mean fluorescence of total >120 pixels (per pixel size 10 µm × 10 µm), in which the fluorescence intensity of each pixel was determined by ImageJ software (Wayne Rasband, NIH, USA). These pixels were afforded by equally dividing at least 10 independent imaging areas (~ 1200 µm^2^ each) that were randomly selected in the all obtained confocal images. Error bars represent standard deviation (s.d.). The extent of phase separation/transition of RNA was also quantified by the index of dispersion (σ^2^/µ). In brief, the each fluorescence intensity per pixel (pixel size 3.7 µm × 3.7 µm) divided equally from a imaging area (~2500 µm^2^) was determined by ImageJ software (Wayne Rasband, NIH, USA), and followed by that the mean fluorescence intensity (µ) was normalized as well as calculation of standard deviation (σ^2^) among ~180 pixels. Total 10 independent imaging areas (~2500 µm^2^ each) were analyzed for each condition to achieve a representative measure across the sample. Each data point in the bar graphs represents one imaging area. This algorithm accurately identified the inhomogeneity of RNA aggregations, as depicted in Supplementary Fig. 9. For the image of FRAP assay, in each group experiments, 3 independent experiments were completed and >30 images obtained as time dependence in each result, 3 or 4 images at respective scheduled time point were selected from total >30 images during the entire analysis profile around 60 s, and presented. For the fluorescence image in cells, in each group experiments, 3 independent experiments were completed and >10 images obtained in each result. At least 5 images that were randomly selected from the totally obtained confocal images >10 pieces containing intact cells, in which the representative images was divided into at least 16 subunits with equal size and the most representative one finally defined as presentation. The extent of aggregated extent of RNA foci in living cells was quantified by the mean fluorescence intensity from total >30 pixels (per pixel size 5 µm × 5 µm), in which the fluorescence intensity of each pixel was determined by ImageJ software (Wayne Rasband, NIH, USA). These pixels were afforded by equally dividing at least 5 independent imaging areas (~ 400 µm^2^ each) that were randomly selected in the all obtained confocal images. For the imunofluorescence image in cell, the percentage of co-localization was expressed as the number of nuclei presenting co-localization/total number of nuclei containing RNA aggregates. Three independent transfections total thirty cells were counted. Results are presented as mean ± interquartile. In each group experiments, 3 independent experiments were completed and >10 images obtained in each result. At least 5 images that were randomly selected from the totally obtained confocal images >10 pieces containing intact cells, in which the representative images was divided into at least 4 subunits with equal size and the most representative one finally defined as presentation. For the RNA aggregation toxicity assay, in each group experiments, 3 independent experiments were completed and >10 images obtained in each result. At least 5 images that were randomly selected from the totally obtained confocal images >10 pieces containing intact cells, in which the representative images was divided into at least 4 subunits with equal size and the most representative one finally defined as presentation.

### General

All reagents were commercial and from TCI (Tokyo Chemical Industry Co., Ltd.), Sigma-Aldrich or Wako (Wako Pure Chemical Industries, Ltd.) and used without extra purification unless otherwise labeled. All experiments involving air and/or moisture sensitive reagents were performed under an Ar environment. Thin-layer chromatography was completed using TLC Silica gel 60 F_254_ (Merck). The middle pressure liquid chromatography (MPLC) system was employed using EPCLC-AI-580S (Yamazen Corporation, Japan) installed with silica gel column (Hi-Flash Column, Yamazen Corporation). ^1^H NMR (400 MHz), ^19^F NMR (100 MHz) and ^31^P-NMR spectra were recorded on a BRUKER (AV-400M) magnetic resonance spectrometer. DMSO-d_6_ was employed as the solvent. Coupling constants (*J*) values are indicated in Hz and corrected to within 0.5 Hz. Signal patterns are shown as s, singlet; d, doublet; t, triplet; q, quartet; m, multiplet. High-resolution mass spectra (HRMS) were recorded by electrospray ionization (ESI) on a Thermo Scientific Q Exactive instrument (SN01600L, Thermo Fisher Scientific). MALDI-TOF mass spectra were produced on an autoflexIII TOF mass spectrometer (Bruker Daltonics, Billerica, MA, USA).

### 2’,3’,5’-tri-*O*-acetylguanosine (1)

Guanosine (3.0 g, 10.4 mmol), trimethylamine (11.7 mL, 82.8 mmol) and 4-dimethylaminopyridine (138 mg, 1.12 mmol) were dissolved in 40.5 mL anhydrous acetonitrile, acetic anhydride (3.3 mL, 33 mmol) was added dropwise and the mixture incubated for additional 1.5 h at 0 °C and more 1 h at room temperature. The reaction was quenched by adding methanol (3.45 mL, 85.36 mmol). The volume was minimized to 1/3 using a vacuum evaporator and diethyl ether was added to precipitate white solid. The product was obtained by filtration, washed with diethyl ether, and then stirred for 2 h with acetone (60 mL) at 50 °C. The filtrate generated 3.6 g (94%) of white powder. ^1^H NMR (400 MHz, DMSO-d_6_) δ 10.73 (s, 1H), 7.94 (s, 1H), 6.55 (s, 2H), 5.99 (d, *J* = 8.0 Hz, 1H), 5.80 (t, *J* = 8.0 Hz, 1H), 5.50 (dd, *J* = 4.0 Hz, 1H), 4.41-4.25 (m, 3H), 2.12-2.05 (m, 9H); HRMS (ESI) for C_22_H_35_N_6_O_8_ [M + TEA + H]^+^: Calcd. 511.2507; Found. 511.2494.

### 2’,3’,5’-tri-*O*-acetyl-8-trifluoromethylguanosine (2)

2’,3’,5’-tri-*O*-acetyl-guanosine (3.6 g, 7.6 mmol) and Zinc Trifluoromethanesulfinate (4.5 g, 24.4 mmol) were executed in dimethylsulfoxide (92 mL) with dramatic stirring. After the solution turn to be transparent (around 20 min), tert-butyl hydroperoxide (70% aqueous, 5.12 mL, 38.4 mmol) in 10 aliquots (512 μL each) in 25 min intervals. The mixture progressivelly turn pale yellow when addition of tert-butyl hydroperoxide and continue kept for 24 h at room temperature. The mixture was diluted into 600 mL water and extracted with dichloromethane (3 × 160 mL). The collected organic layers were washed with water (3 × ~100 mL), brine (~100 mL) and dried over with sodium sulfate. The obtained agent was filtered, washed with dichloromethane and the filtrate was evaporated in vacuum. The oily crude product was purified using MPLC with the mixture of methanol and dichloromethane (5%, v/v) to give white product (3.2 g, 55%). ^1^H NMR (400 MHz, DMSO-d_6_) δ 12.20 (s, 1H), 6.29 (s, 1H), 5.95 (t, J = 6.4 Hz, 2H), 4.55-4.52 (m, 1H), 4.46-4.38 (m, 2H), 2.15-2.04 (m, 9H); ^19^F NMR (372 MHz, DMSO-d_6_) δ -61.1843 (s, 3F); HRMS (ESI) for C17H15O8N5F3Na [M+Na]^+^: Calcd. 500.1065; Found. 500.0981.

### 8-trifluoromethylguanosine (3)

2’,3’,5’-tri-*O*-acetyl-8-trifluoromethylguanosine (3.2 g,6.68 mmol) was taken in 400 mL flask. Methylamine (33% in ethanol, 35.52 mL, 340.5 mmol) was complemented and leaded to a reaction mixture, and reacted for 4 h at room temperature. The solution was evaporated in vacuum and the crude sample was purified by MPLC with the mixture of methanol and dichloromethane (10%, v/v). The product was given as yellow powder (2.0 g, 94%). ^1^H NMR (400 MHz, DMSO-d_6_) δ 11.05 (s, 1H), 6.72 (s, 2H), 5.64 (d, *J* = 6.0 Hz, 1H), 5.50 (d, *J* = 4.4 Hz, 1H), 5.14–4.92 (m, 3H), 4.17 (dd, *J* = 4.8 Hz, 1H), 3.91 (dd, *J* = 3.2 Hz, 1H), 3.71-3.52 (m, 2H); ^19^F NMR (372 MHz, DMSO-d_6_) δ -59.8351 (s, 3F); HRMS (ESI) for C_11_H_11_O_5_N_5_F_3_ [M-H]^−^: Calcd. 350.0688; Found. 350.0695.

### *N2*-dimethylformamidyl-8-trifluoromethylguanosine (4)

8-Trifluoromethylguanosine (2.0 g, 5.68 mmol) and N,N-dimethylformamide dimethyl acetal (5.42 mL, 40.2 mmol) were mixed with anhydrous dimethylformamide (40 mL). The mixture was incubated for 1 h at room temperature and evaporated in vacuum. The crude product was purified by MPLC with mixture of methanol and dichloromethane (18%, v/v), the product was given as white foam (1.92 mg, 84%). ^1^H NMR (400 MHz, DMSO-d_6_) δ 11.76 (s, 1H), 8.54 (s, 1H), 5.68 (d, *J* = 6.0 Hz, 1H), 5.42 (d, *J* = 6.4 Hz, 1H), 5.24 (d, *J* = 4.8 Hz, 1H), 5.05 (*J* = 6.0 Hz, 1H), 4.89 (*J* = 4.6 Hz, 1H), 4.27 (dd, *J* = 5.2 Hz, 1H), 3.94 (dd, *J* = 3.6 Hz, 1H), 3.72-3.56 (m, 2H), 3.18 (s, 3H), 3.08 (s, 3H); HRMS (ESI) for C_14_H_16_O_5_N_6_F_3_ [M-H]^−^: Calcd. 405.1231; Found. 405.1225.

### *N2*-dimethylformamidyl-8-trifluoromethyl-2’-*O*-(tert-butyldimethylsilyl)-3’,5’-*O*-(di-tert-butylsilylene)guanosine (5)

*N2*-dimethylformamidyl-8-trifluoromethylguanosine (400 mg, 0.96 mmol) was mixed with anhydrous dimethylformamide (4 mL), and di-tert-butylsilyl bis (trifuoromethanesulfonate) (460 mg, 1.06 mmol) was added dropwise. The mixture kept for 15 min at 0 °C. Imidazole (327 mg, 4.8 mmol) was complemented and continue to reacted for 15 min at 0 °C and further 15 min at room temperature. Tert-butyldimethylsilyl chloride (691.2 mg, 4.6 mmol) was added, the mixture reacted for 4 h at 60 °C. The mixture was concentrated in vacuo and the crude sample was purified by MPLC in the mixture of chloroform and ethyl acetate (25%, v/v). The product was given as a white foam (520 mg, 89%). ^1^H NMR (400 MHz, DMSO-d_6_) δ 9.19 (s, 1H), 8.38 (s, 1H), 8.01 (s, 1H), 5.82 (d, *J* = 1.2 Hz, 1H), 5.09 (dd, *J* = 2.0 Hz, 1H), 4.52 (dd, *J* = 6.0 Hz, 1H), 4.42 (dd, *J* = 4.8 Hz, 1H), 4.06 (*dt*, *J* = 5.2 Hz, 1H), 3.93 (t, *J* = 6.8 Hz, 1H), 3.18-3.14 (m, 7H), 2.95-2.87 (m, 6H), 1.68 (s, 6H), 1.06–0.86 (m, 45H), 0.08-0.04 (m, 12H); HRMS (ESI) for C28H48O5N6F3Si2 [M + H]^+^: Calcd. 661.3099; Found. 661.3160.

### *N2*-dimethylformamidyl-8-trifluoromethyl-5’-*O*-(4,4’-dimethoxytrityl)-2’-*O*-tert-butyldimethylsilylguanosine (6)

Compound 5 (660 mg, 1.0 mmol) was taken in 4.5 mL dichloromethane, and 109.2 μL hydrofluoric acid-pyridine solution (70% hydrofluoric acid, 30% pyridine) with 0.68 mL pyridine were added, and stirred at 0 °C for 2 h. The mixture was extracted by dichloromethane and integrated organic layer concentrated in vacuum for producing residue 520 mg. Without additional purification, the sample (520 mg) with 4,4’-dimethoxytrityl chloride (483.2 mg, 1.43 mmol) were dissolved in 6 mL anhydrous pyridine and stirred for further 4 h at room temperature. The solvent was evaporated in vacuum and purified using MPLC in the mixture of dichloromethane and ethyl acetate (25%, v/v). The product was given as a white foam (507 mg, 60%).^1^H NMR (400 MHz, DMSO-d_6_) δ 9.05 (s, 1H), 8.61 (dt, *J* = 12 Hz, 1H), 8.20 (s, 1H), 7.41-7.14 (m, 8H), 6.77-6.73 (m, 4H), 5.81 (d, *J* = 4.0 Hz, 1H), 5.14 (dd, *J* = 4.0 Hz, 1H), 4.54 (dd, *J* = 6.4 Hz, 1H), 4.02 (dd, *J* = 12.4 Hz, 1H), 3.76 (s, 6H), 3.44-3.40 (m, 2H), 2.99 (s, 3H), 2.63 (s, 3H), 2.04 (s, 1H), 0.86 (s, 10H), 0.118 (s, 3H), 0.008 (s, 3H); HRMS (ESI) for C_41_H_49_O_7_N_6_F_3_SiNa [M+Na]^+^: Calcd.845.3384; Found. 845.3332.

### 3’*-O*-[(2-Cyanoethoxy)(diisopropylamino)phosphino]-*N2*-dimethylformamidyl-8-trifluoromethyl-5’-*O*-(4,4’-dimethoxytrityl)-2’-*O*-tert-butyldimethylsilylguanosine (7)

Compound 6 (1.0 g, 2.42 mmol) evaporated using 6 mL anhydrous acetonitrile as three times and mixed with 16 mL anhydrous dichloromethane. Diisopropylethylamine (1.56 mL, 9.0 mmol) and 1-methylimidazole (0.18 mL, 2.25 mmol) were complemented. After 5 min, 2-cyanoethyl-*N,N*-diisopropylamidochlorophosphoramidite (1.5 mL, 7.26 mmol) was added, the reaction mixture was performed for 1.5 h at room temperature. The mixture was extracted with dichloromethane and aqueous layer, followed by that concentrated in vacuum, the obtained crude sample was purified using MPLC in the mixture of ethyl acetate and dichloromethane (25%, v/v). The compound was given as a white foam (1.6 g, 70%). ^1^H NMR (400 MHz, DMSO-d_6_) δ 8.76 (s, 1H), 8.03 (s, 1H), 7.92 (s, 1H), 7.48-7.17 (m, 12H), 6.78-6.72 (m, 5H), 5.91-5.88 (m, 1H), 5.30 (m, 1H), 4.58-4.31 (m, 2H), 4.11 (s, 1H), 3.76-3.47 (m, 16H), 2.98-2.56 (m, 5H), 2.31 (s, 1H), 2.10-2.02 (m, 2H), 1.29-0.73 (m, 30H), 0.08-0.00 (m, 11H); ^31^P NMR (161 MHz, DMSO-d_6_) δ 150.86, 148.17; HRMS (ESI) for C_56_H_81_O_8_N_9_F_3_ [M + TEA + H]^+^: Calcd. 1124.5782; Found. 1124.5729.

### Reporting summary

Further information on research design is available in the Nature Portfolio Reporting Summary linked to this article.

### Supplementary information

Supplementary Information

Description of Additional Supplementary Files

Supplementary Data 1

Supplementary Data 2

Supplementary Movies 1–18

Reporting Summary

## Data Availability

All data supporting the findings of this study are available within the paper and its Supplementary Information. The source files for all the graphs presented in the paper, Supplementary Data 1, 2, and supplementary movies are also available in Dryad (10.5061/dryad.sj3tx96bz).
