# Supplementary material for: RNA structure promotes liquid-to-solid phase transition of short RNAs in neuronal dysfunction
Source: Commun Biol. 2024 Jan 29;7:137. doi: 10.1038/s42003-024-05828-z (PMC10824717; doi:10.1038/s42003-024-05828-z)
Supplement: Supplementary file 1 — Supplementary Information [file 42003_2024_5828_MOESM1_ESM.pdf]

## Supplementary Information

## Supplementary Table 1

| **DNA/RNA sequences** | **Name** | **Cy3 labeled DNA/RNAs** | **Name** |
|---|---|---|---|
| r(GCGGCGGC) | RNA-1 | Cy3-r(GCGGCGGC) | FRNA-1 |
| r(GC$^{F}$GGCGGC) | RNA-2 | Cy3-r(GC$^{F}$GGCGGC) | FRNA-2 |
| r(CCGGGGCC) | RNA-3 | Cy3-r(CCGGGGCC) | FRNA-3 |
| r(GCGGCAGC) | RNA-4 | Cy3-r(GCGGCAGC) | FRNA-4 |
| r(GC$^{F}$GGCAGC) | RNA-5 | Cy3-r(GC$^{F}$GGCAGC) | FRNA-5 |
| r(UAGGGU) | Tel-RNA-6 | | |
| r(UAGGGU)$_2$ | Tel-RNA-7 | | |
| r(UAGGGU)$_4$ | Tel-RNA-8 | | |
| r(UAG$^{Br}$GGU), G is brominated | Tel-RNA-9 | | |
| r(GGGUUAGGG) | Tel-RNA-10 | | |
| rAGGG(UUAGGG)$_3$ | Tel-RNA-11 | | |

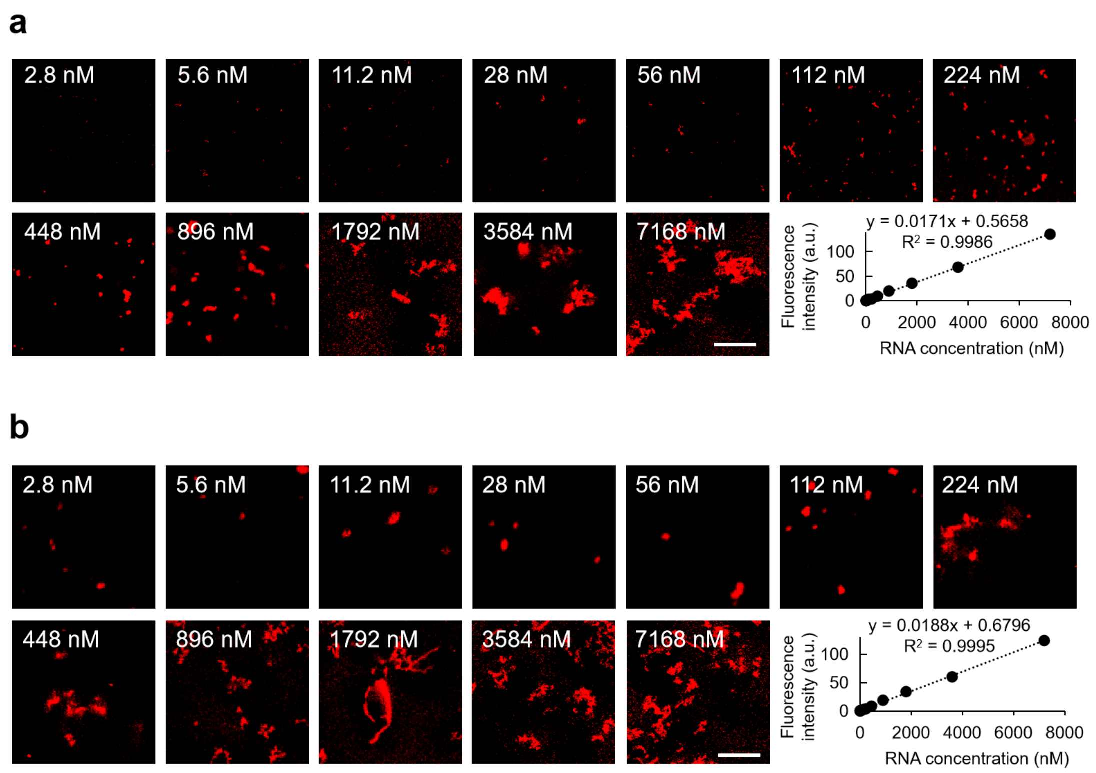


**Supplementary Figure 1. Aggregation of short RNAs at indicated concentrations.** (A) Fluorescence micrographs for indicated FRNA-1 clusters with different RNA concentrations at 40% PEG, in which RNA concentration represents the concentration of trinucleotide repeats (47×) converted from 8-mer CGG repeats at equivalent nucleoside concentrations. Scale bars, 5 µm. (B) Fluorescence micrographs for indicated FRNA-2 clusters with different RNA concentrations with 1000 nM FMRP, in which RNA concentration represents the concentration of trinucleotide repeats (47×) converted from 8-mer CGG repeats at equivalent nucleoside concentrations. Scale bars, 5 µm.

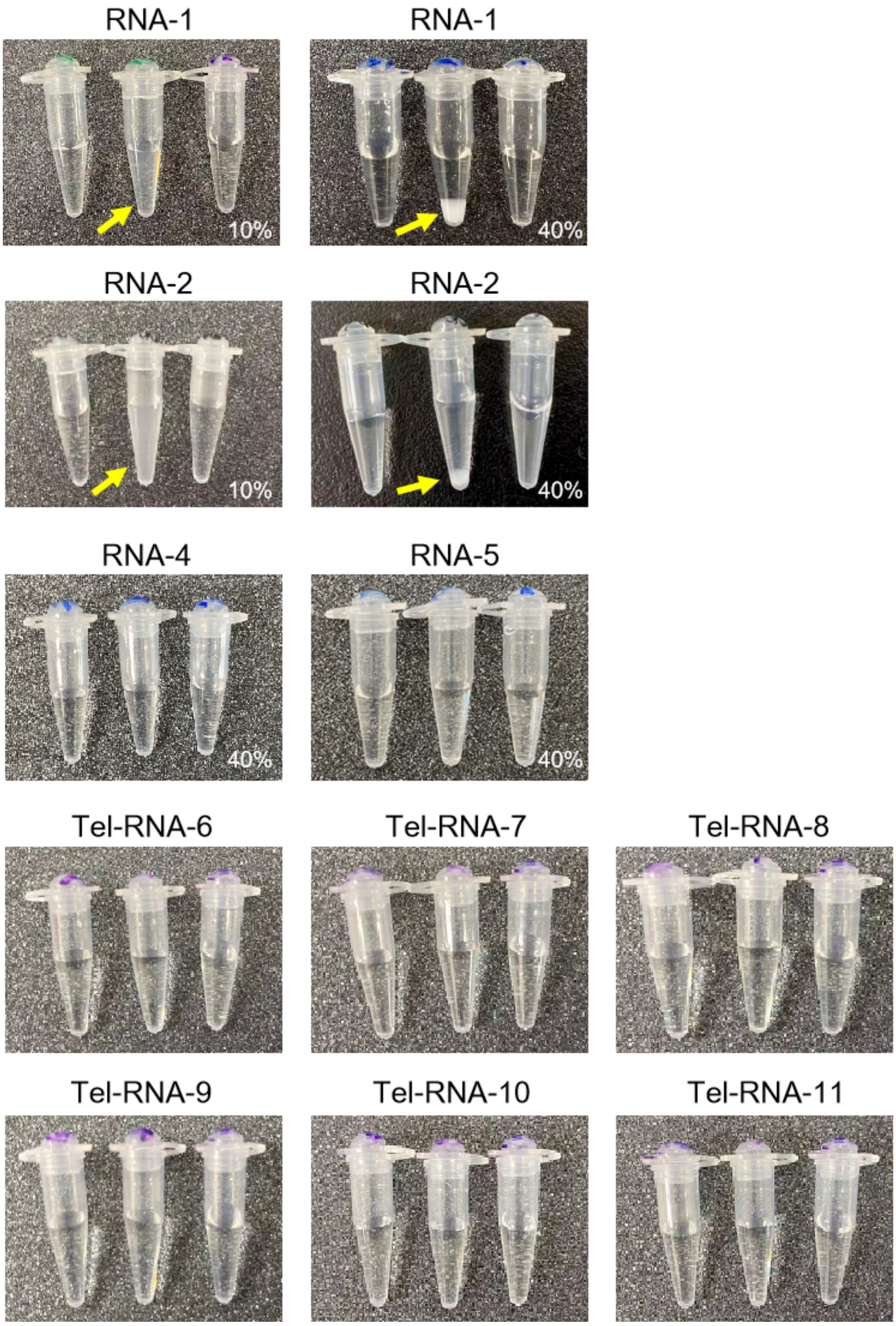


**Supplementary Figure 2. Aggregation of short RNAs.** Mixing PEG (right tube) with RNA-1 and RNA-2 (left tube) immediately resulted in a turbid solution (10% PEG) or solid-like state (centre tube) (40% PEG) as indicated by arrows, while mixing 40% PEG (right tube) with control RNA-4, RNA-5, and different lengths of telomere RNA UUAGGG repeats (left tube) remained unchanged (centre tube).

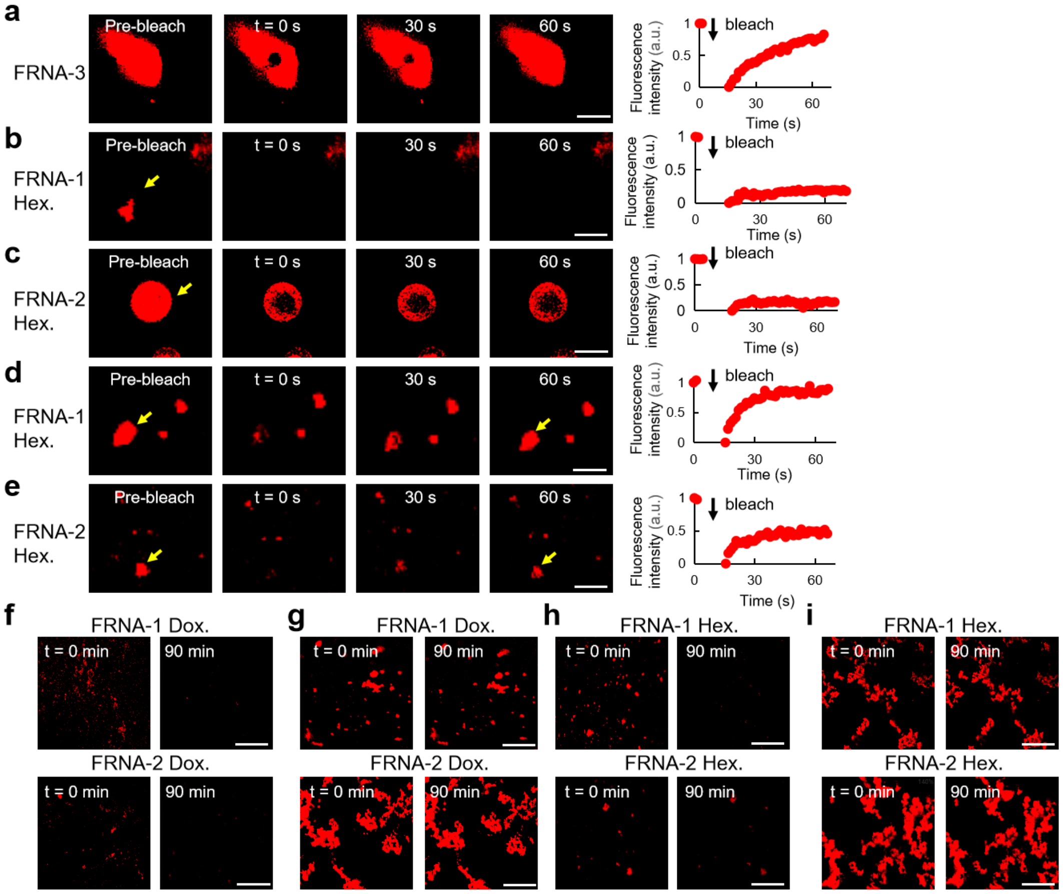


**Supplementary Figure 3. FRAP assay and time-dependence experiments. a,** FRAP for FRNA-3 before and after photobleaching at 40% PEG. Graphs represent recovery plot corresponding to **a**. Scale bars, 5 µm. **b, c**, Inhibitor 1,6-hexanediol in fluorescence recovery after photobleaching experiments for FRNA-1 and FRNA-2 before and after photobleaching (arrow, bleach cluster) at 40% PEG. Graphs represent recovery plot corresponding to **b** and **c**. Scale bars, 5 µm. **d, e**, Inhibitor 1,6-hexanediol in fluorescence recovery after photobleaching experiments for FRNA-1 and FRNA-2 before and after photobleaching (arrow, bleach cluster) at 10% PEG. Graphs represent recovery plot corresponding to **d** and **e**. Scale bars, 5 µm. **f, g,** FRNA-1 or FRNA-2 aggregation was inhibited by doxorubicin as time-dependent at 10% (**f**) and 40% (**g**) PEG. Scale bars, 50 µm. **h, i,** FRNA-1 or FRNA-2 aggregation was inhibited by 1,6- hexanediol as time-dependent at 10% (**h**) and 40% (**i**) PEG. Scale bars, 50 µm.

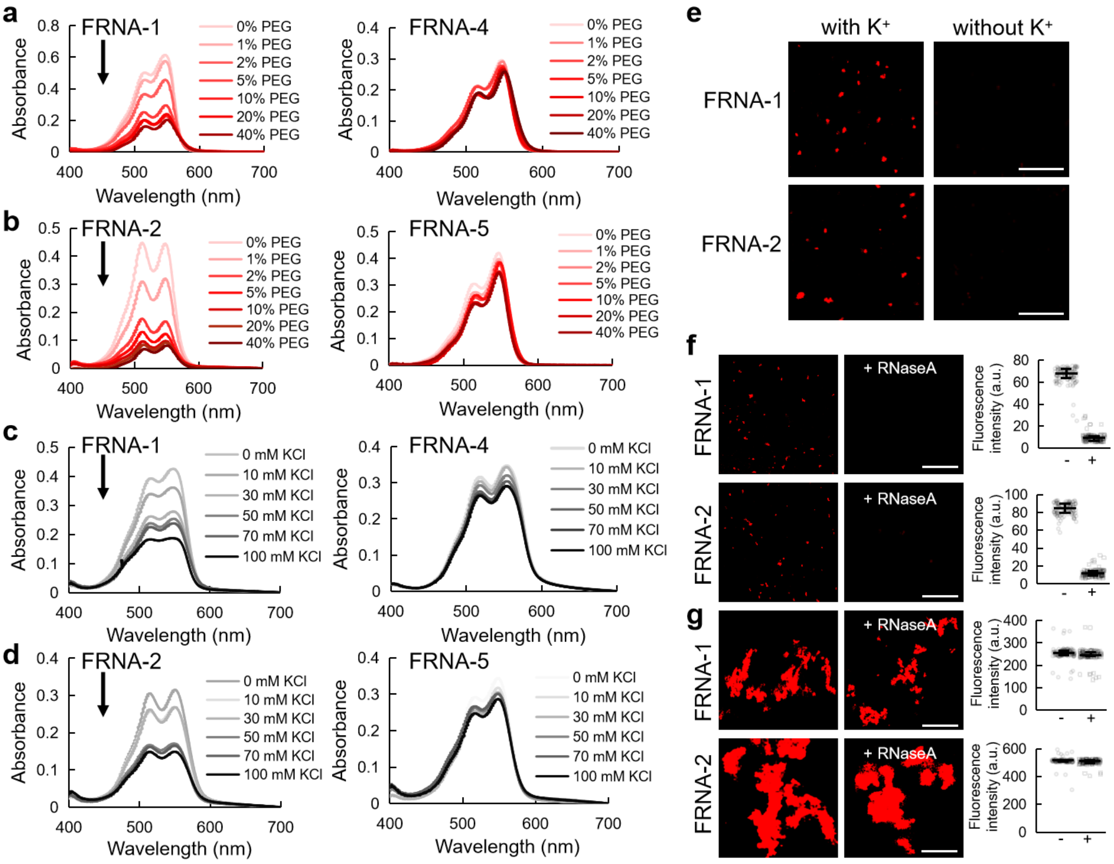


**Supplementary Figure 4. PEG and metal ion effect on RNA aggregation. a, b,** Absorbance (400 ~ 700 nm) of FRNA-1, FRNA-2, and control FRNA-4, FRNA-5 reduced at varying degrees after adding different ratios of PEG within 150 mM KCl. **c, d,** Absorbance (400 ~ 700 nm) of FRNA-1, FRNA-2, and control FRNA-4, FRNA-5 reduced at varying degrees after adding different concentrations of KCl under 40% PEG. (RNA clusters were precipitated by centrifugation at 12,000*g* for 5 mins before measurement each time). **e,** FRNA-1 and FRNA-2 clusters disappeared without KCl at 10% PEG. Condition: RNAs in 150 mM KCl and 10 mM Tris-HCl, pH 7.0. **f, g,** The FRNA-1 and FRNA-2 clusters were treated with RNaseA for 30 min at room temperature in at 10% (**f**) and 40% (**g**) PEG (n = 120). Error bars represent mean ± interquartile. Scale bars, 50 μm.

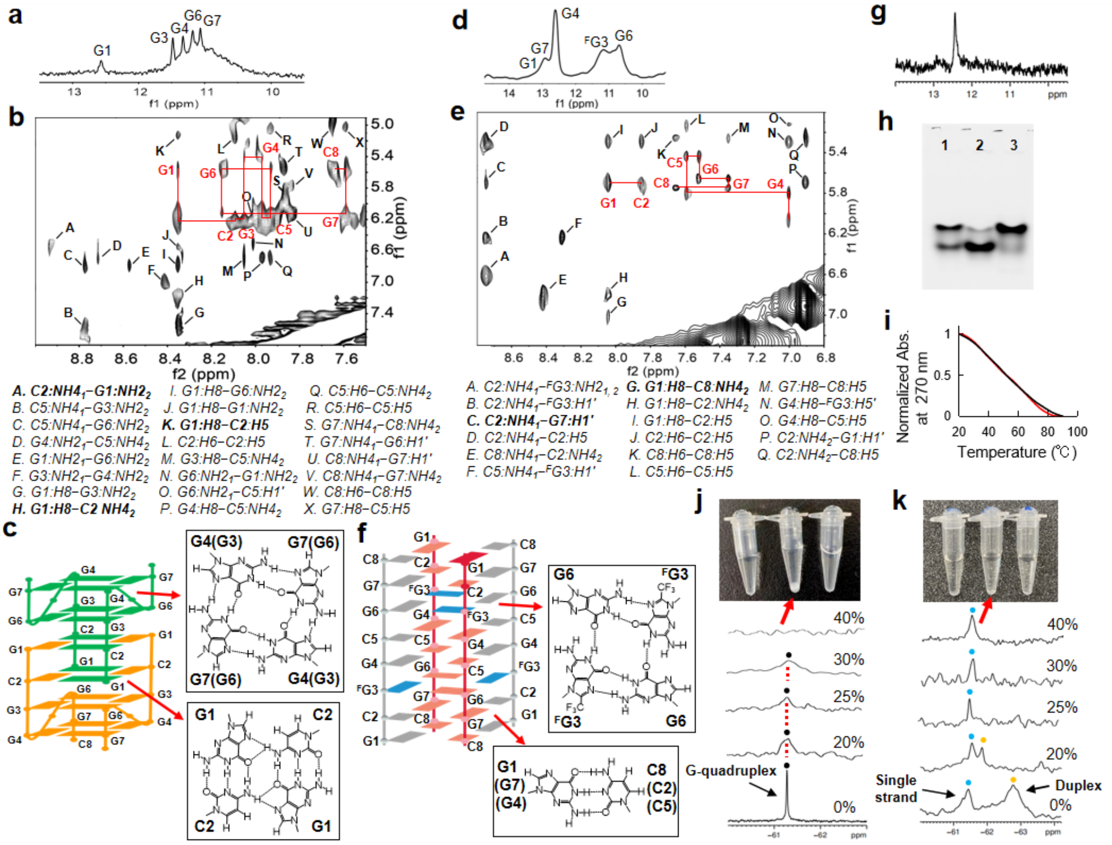


**Supplementary Figure 5. NMR and gel analysis of RNA aggregates. a,** Imino proton NMR spectrum of RNA-1. **b,** Expanded 2D NOESY contour plots of RNA-1. Red line shows sequential pathway in aromatic-anomeric region. The cross peaks A to X are assigned. Cross peaks for A (C2:NH4$_1$ and G1:NH2$_2$), H (G1:H8 and C2:NH4$_2$), and K (G1:H8 and C2:H5) are shown in bold. **c,** Schematic structure of RNA-1 with G3:G6:G3:G6 and G4:G7:G4:G7 G-tetrads and G1:C2:G1:C2 tetrads. **d,** Imino proton NMR spectrum of RNA-2. **e,** Expanded 2D NOESY contour plots of RNA-2. Red line shows sequential pathway in aromatic-anomeric region. The cross peaks A to Q are assigned. Cross peaks for G (G1:H8 and C8:NH4$_2$) and C (C2: NH4$_1$ and G7:H1') are shown in bold. **f,** Schematic structure of RNA-2 with $^F$G3:G6:$^F$G3:G6 tetrads and G:C base pairs. **g,** Imino proton NMR spectrum of RNA-5. **h,** Comparison of gel electrophoretic mobility for the mixture lane 2 and lane 3 RNA (lane 1), FRNA-5 (lane 2), FRNA-1 (lane 3). **i,** CD melting curves for RNA-1 (red line) and RNA-2 (back line) monitored at 270 nm in the presence of 150 mM KCl. **j,** $^{19}$F NMR spectra of RNA-2 in dilute solution and mixing with different concentration PEG 200. The single $^{19}$F signal changed to broad and attenuate corresponding to solid-like state (centre tube). The peaks of G-quadruplex are marked with back spots. **k**, $^{19}$F NMR spectra of RNA-5 in dilute solution and mixing with different concentration PEG 200. RNA remained soluble after mixing with PEG 200. Blue and yellow spots indicated the single strand and duplex.

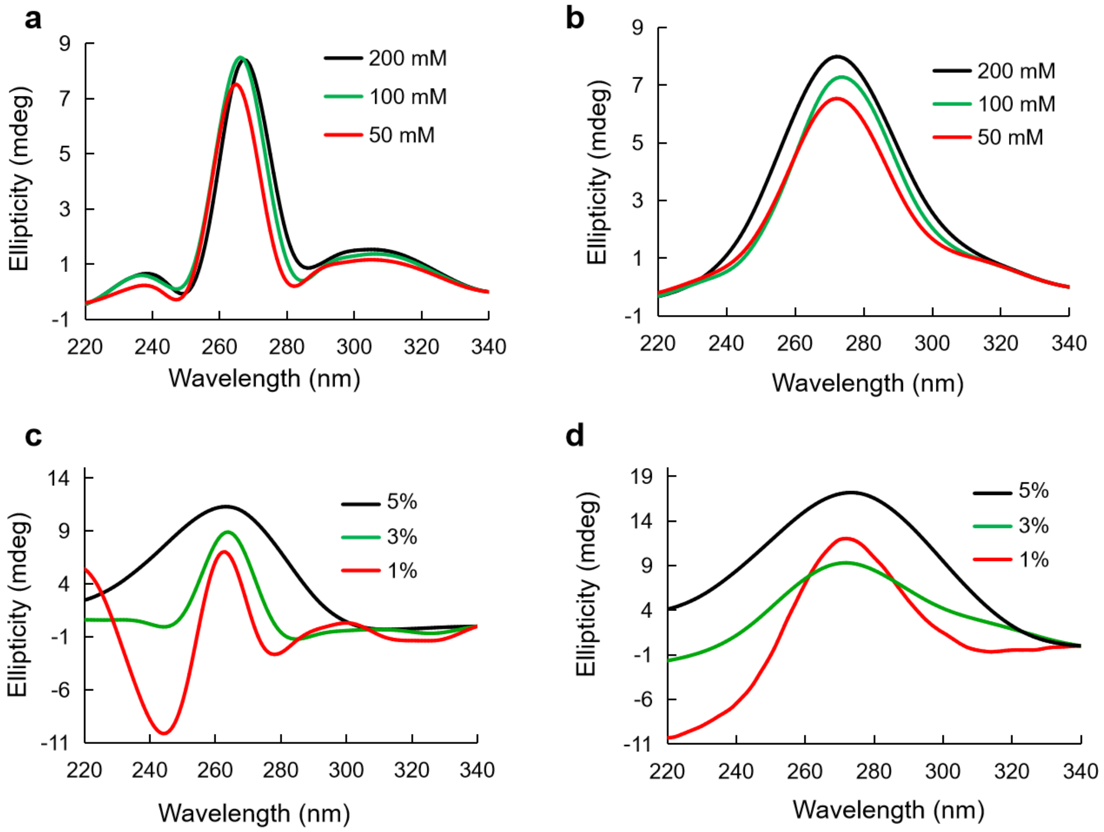


**Supplementary Figure 6. CD spectrum in KCl or PEG with gradient concentration. a,** CD spectrum of RNA-1 in different concentration of KCl at 10°C. **b,** CD spectrum of RNA-2 in different concentration of KCl at 10°C. **c,** CD spectrum of RNA-1 in different concentration of PEG 200 with 100 mM KCl at 10°C.**d,** CD spectrum of RNA-2 in different concentration of PEG 200 with 100 mM KCl at 10°C.

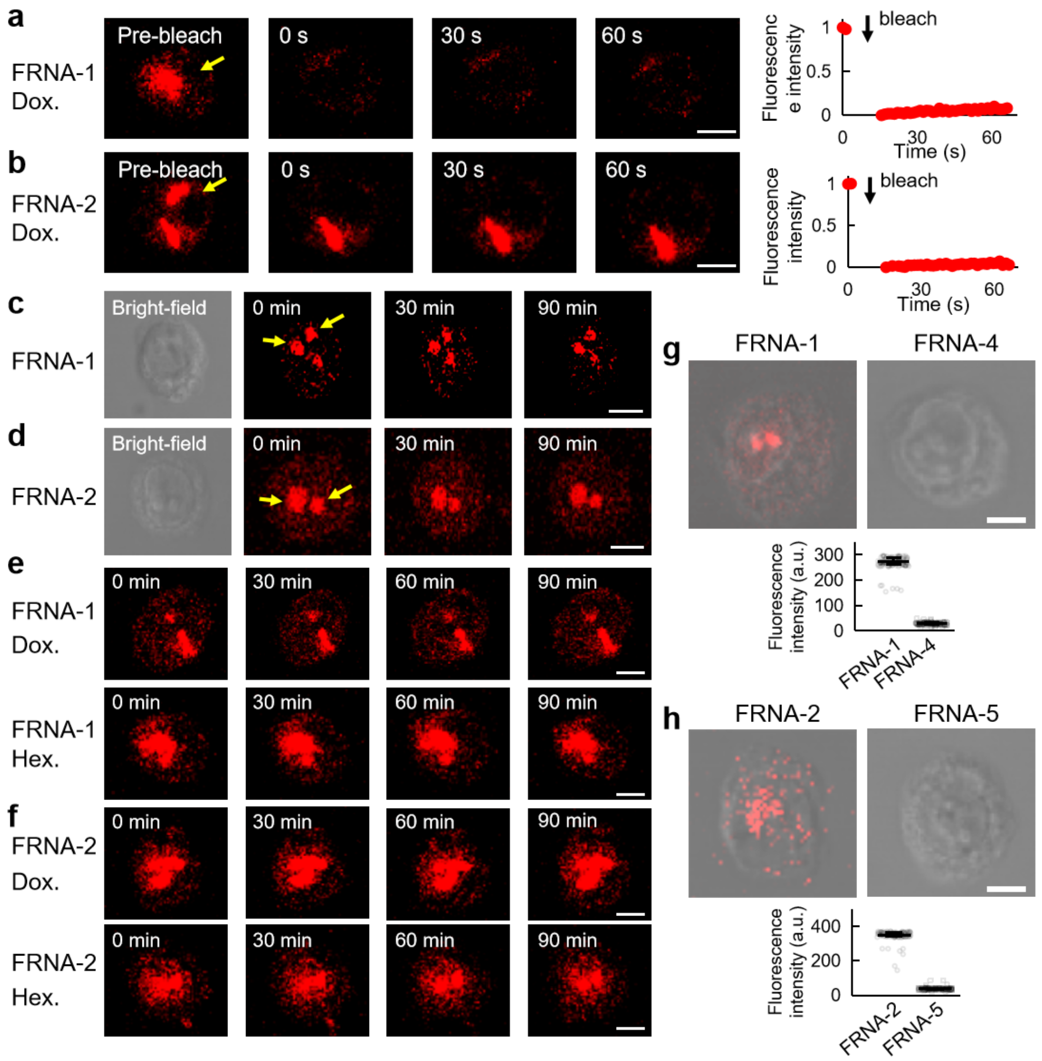


**Supplementary Figure 7. FRAP assay and time-dependence experiments. a,** FRNA-1 clusters localized in HeLa cells treated with inhibitor doxorubicin and analyzed by FRAP (arrow, bleach cluster). Graphs represent recovery plot corresponding to **a. b,** FRNA-2 clusters localized in HeLa cells treated with inhibitor doxorubicin and analyzed by FRAP (arrow, bleach site). Graphs represent recovery plot corresponding to **b. c,** Time-dependence of FRNA-1 clusters in HeLa cells. **d,** Time-dependence of FRNA-2 clusters in HeLa cells. **e,** Time-dependence of FRNA-1 clusters in HeLa cells treated with inhibitor doxorubicin or 1,6-hexanediol. **f,** Time-dependence of FRNA-2 clusters in HeLa cells treated with inhibitor doxorubicin or 1,6- hexanediol. **g,** Micrographs of FRNA-1 and FRNA-4 in HeLa cells (n = 30). **h,** Micrographs of FRNA-2 and FRNA-5 in HeLa cells (n = 3). Error bars represent mean ± interquartile. Scale bars, 5 μm..

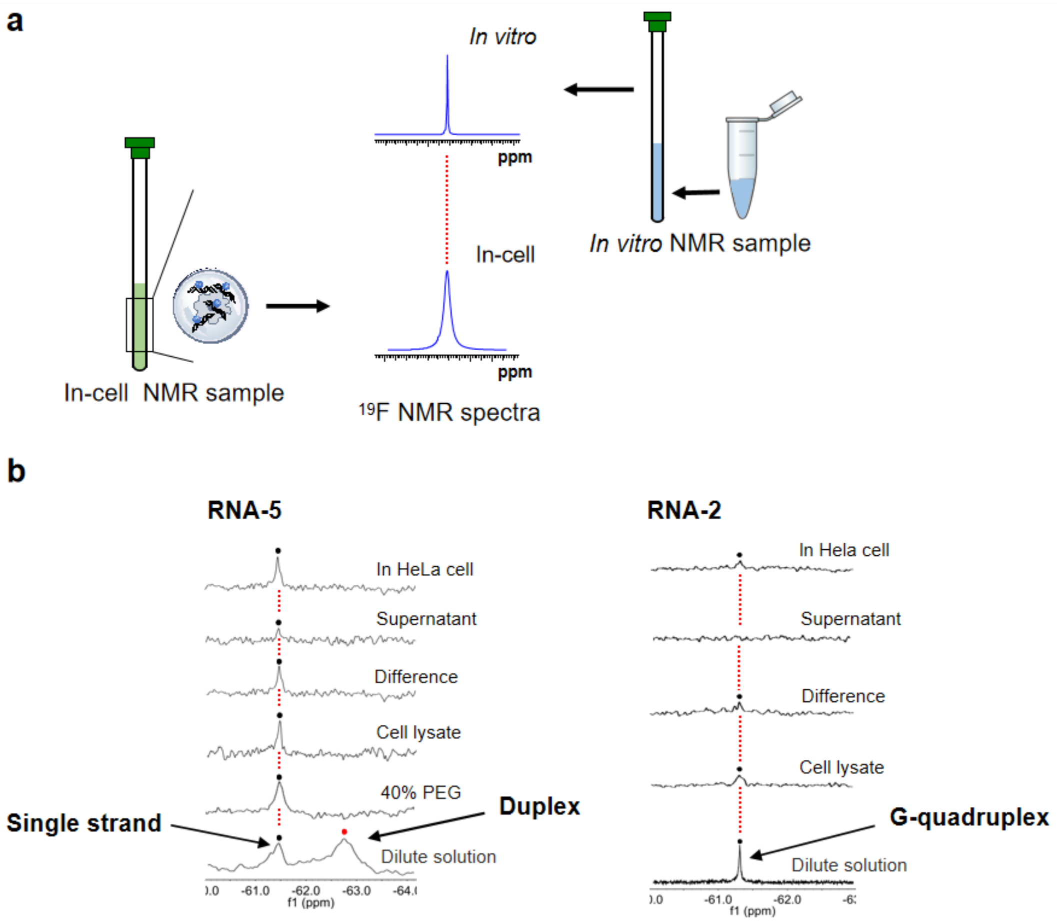


**Supplementary Figure 8. $^{19}$F NMR analysis of RNA aggregations in cells. a,** Schematic overview of in-cell $^{19}$F NMR experiments. The reference *in vitro* spectrum was compared to the in-cell $^{19}$F NMR spectrum, enabling reliable determination of the intracellular RNA conformation. **b,** Comparison of $^{19}$F NMR spectra of RNAs in dilute solution, in HeLa cell, in supernatant, in cell lysate, difference spectrum between HeLa cell and supernatant, and in 40% PEG.

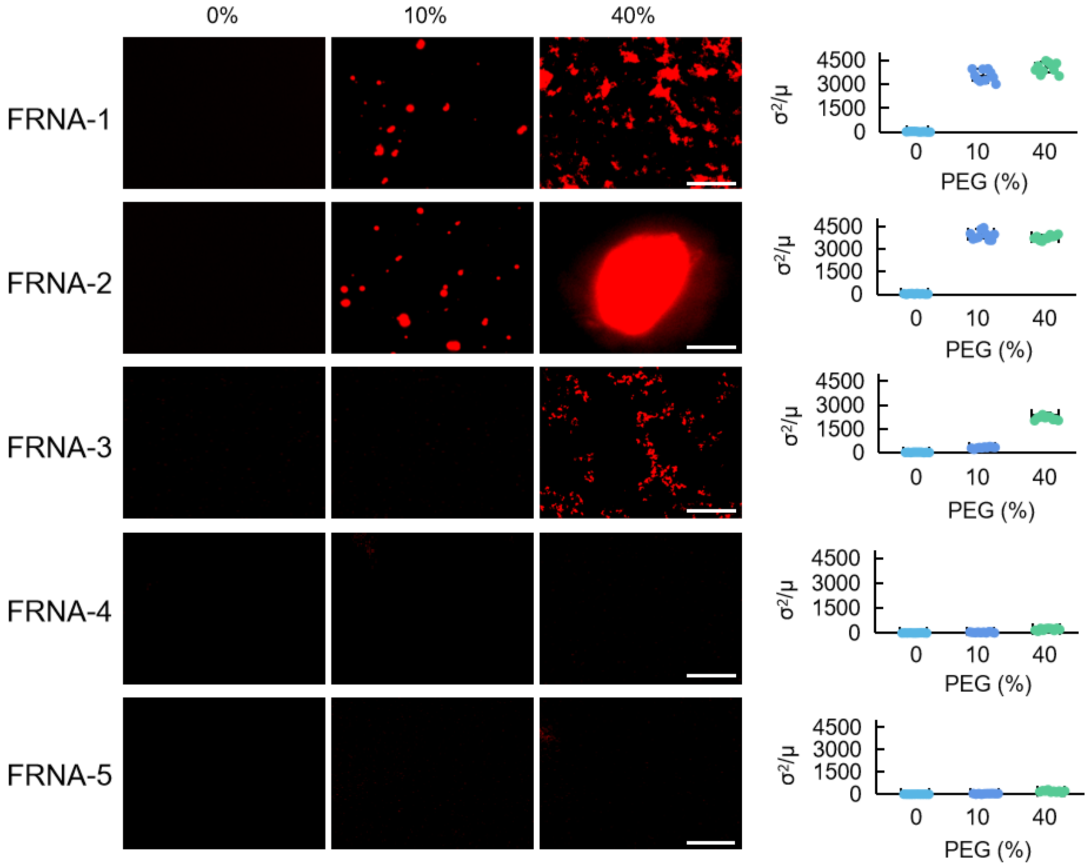


**Supplementary Figure 9. Fluorescence micrographs and quantitation of inhomogeneity as normalized variance ($\sigma^2/\mu$) of RNA clusters for short RNAs aggregation *in vitro*.** Fluorescence micrographs for indicated FRNA-1, FRNA-2, FRNA-3, FRNA-4 and FRNA-5 and with different PEG 200 concentrations (0% – 40%). Quantitation of inhomogeneity as normalized variance ($\sigma^2/\mu$) of RNA clusters with different PEG 200 concentrations (n = 10). Error bars represent mean ± s.d. Scale bars, 50 μm.

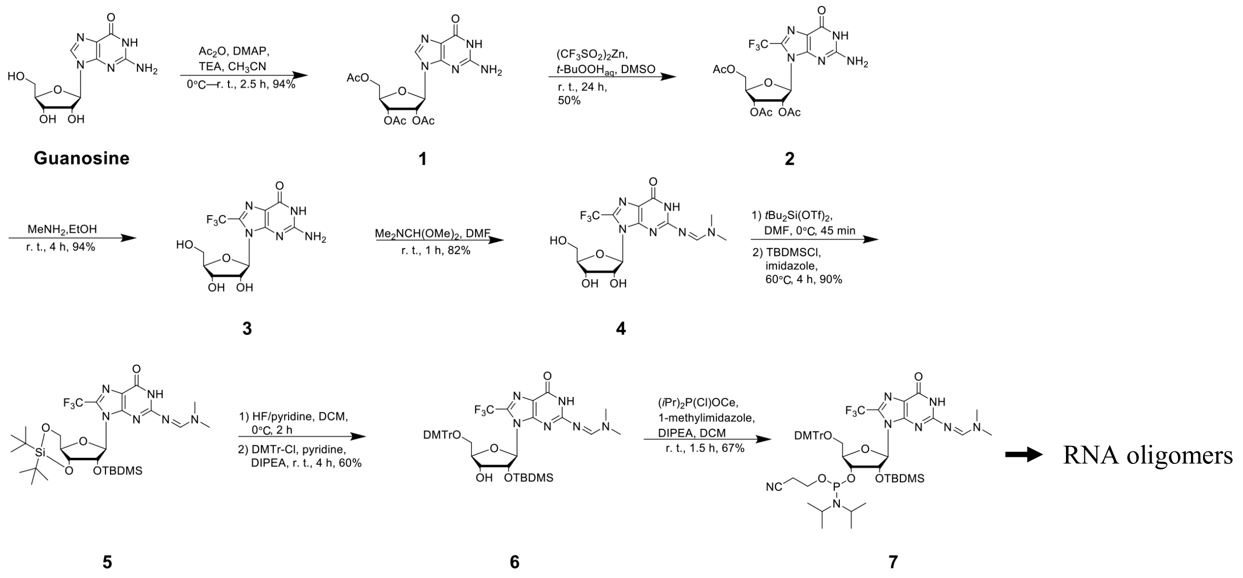


**Supplementary Figure 10.** Scheme for synthesis of 8-trifluoromethyl-guanosine ($^{F}$G) and incorporation into RNA sequences by phosphoramidite chemistry.

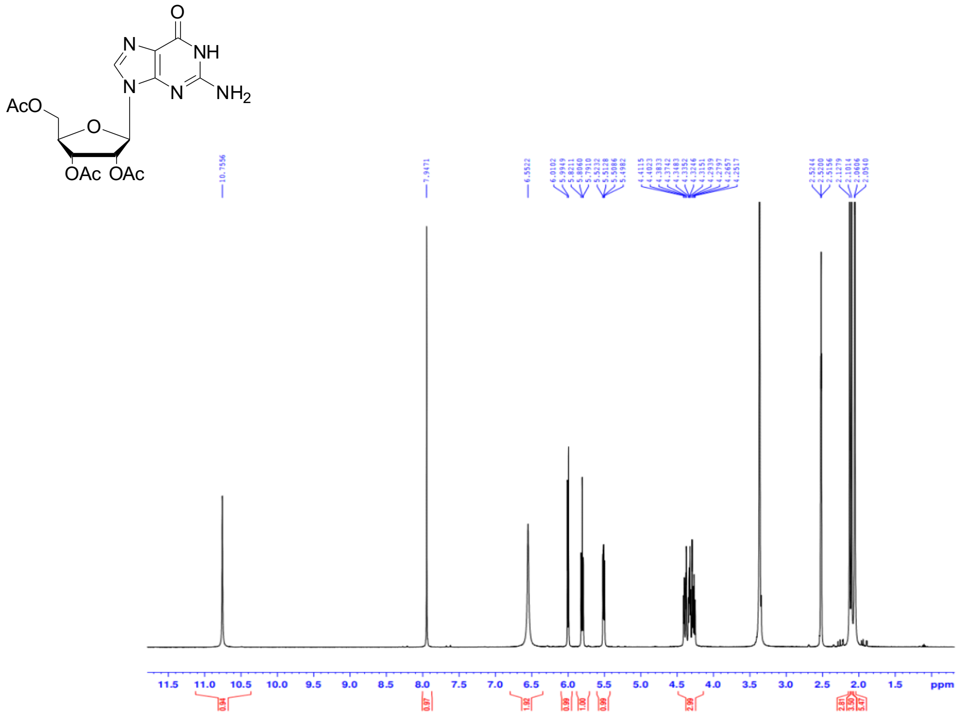

**Supplementary Figure 11.** $^1$H NMR spectrum of 2',3',5'-tri-*O*-acetylguanosine (**1**).

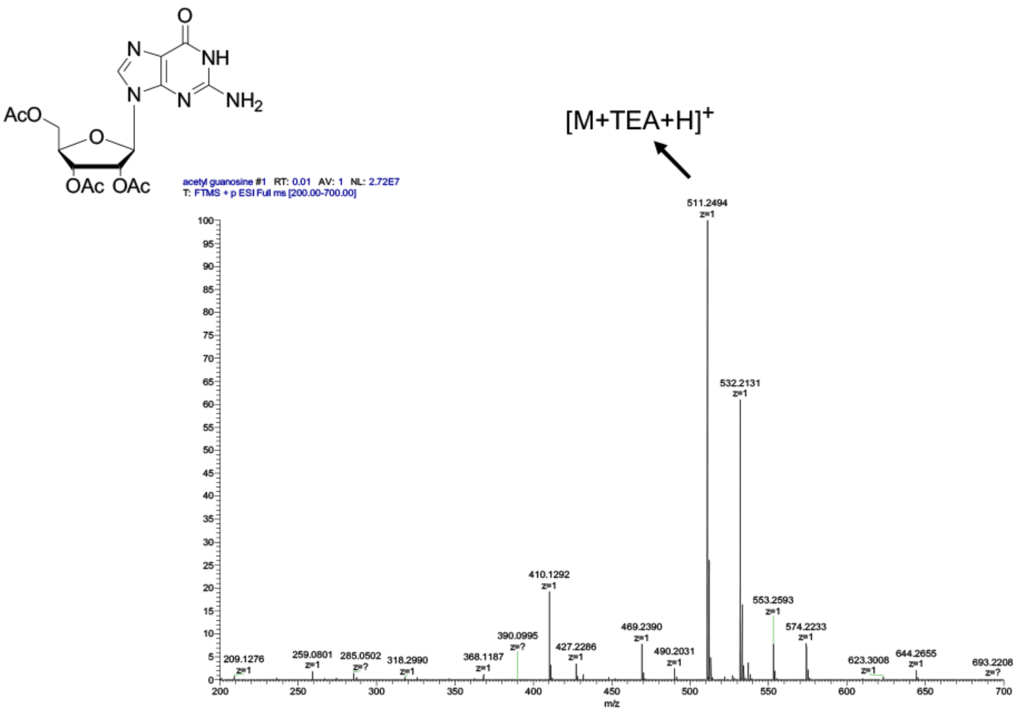


**Supplementary Figure 12.** HRMS spectrum of 2',3',5'-tri-*O*-acetylguanosine (**1**).

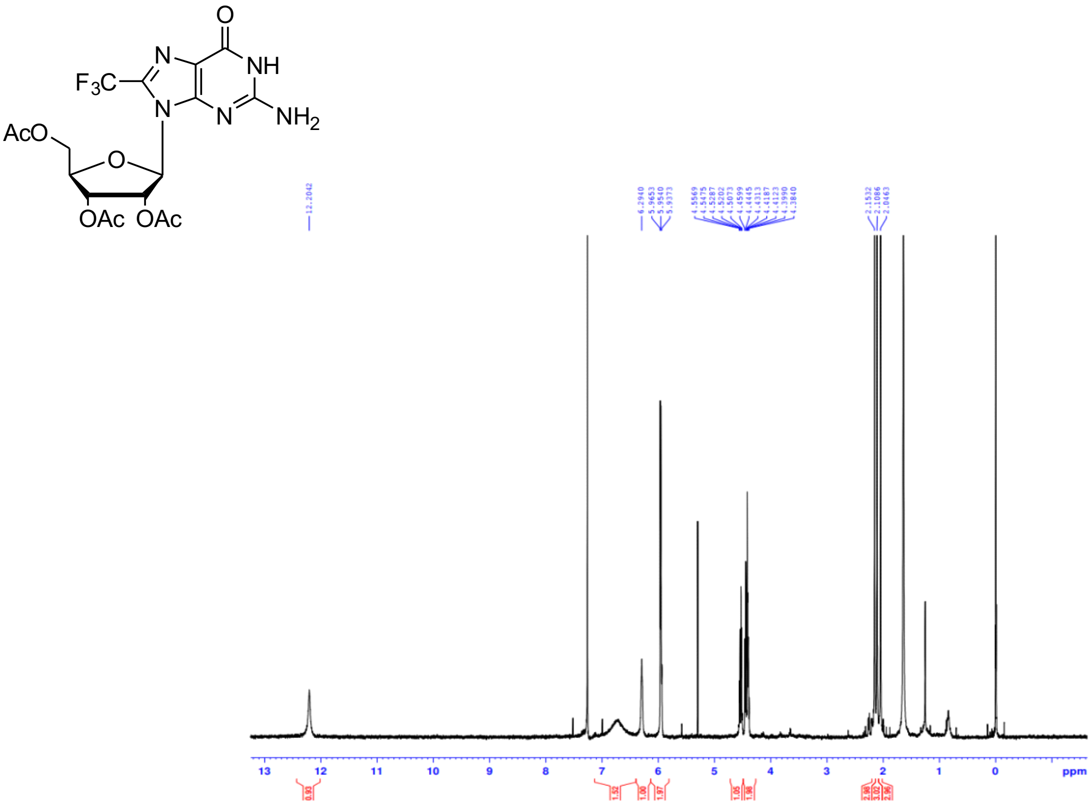

**Supplementary Figure 13.** $^{1}$H NMR spectrum of 2',3',5'-tri-*O*-acetyl-8-trifluoromethylguanosine (**2**).

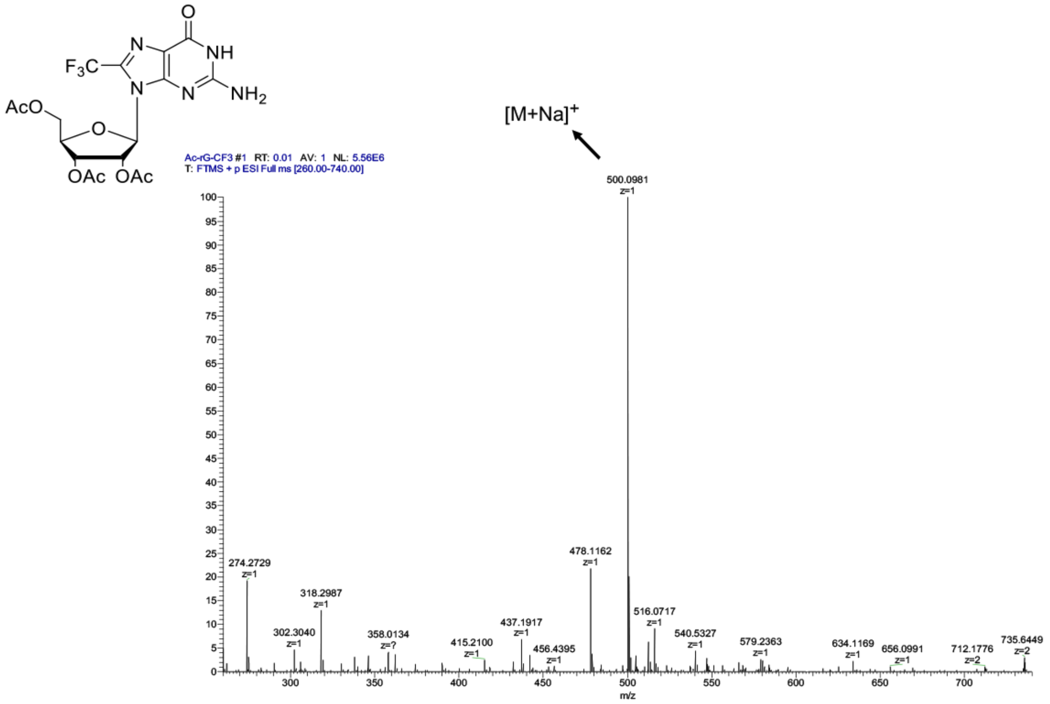


**Supplementary Figure 14.** HRMS spectrum of 2',3',5'-tri-*O*-acetyl-8-trifluoromethylguanosine (**2**).

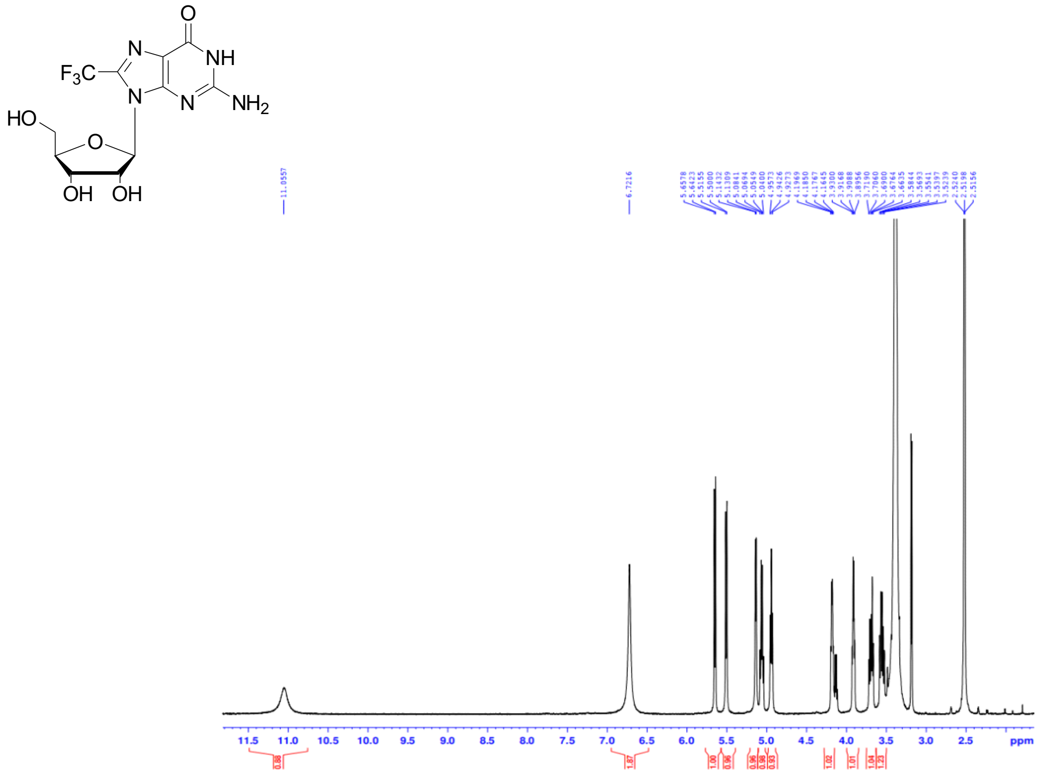


**Supplementary Figure 15.** $^{1}$H NMR spectrum of 8-trifluoromethylguanosine (**3**).

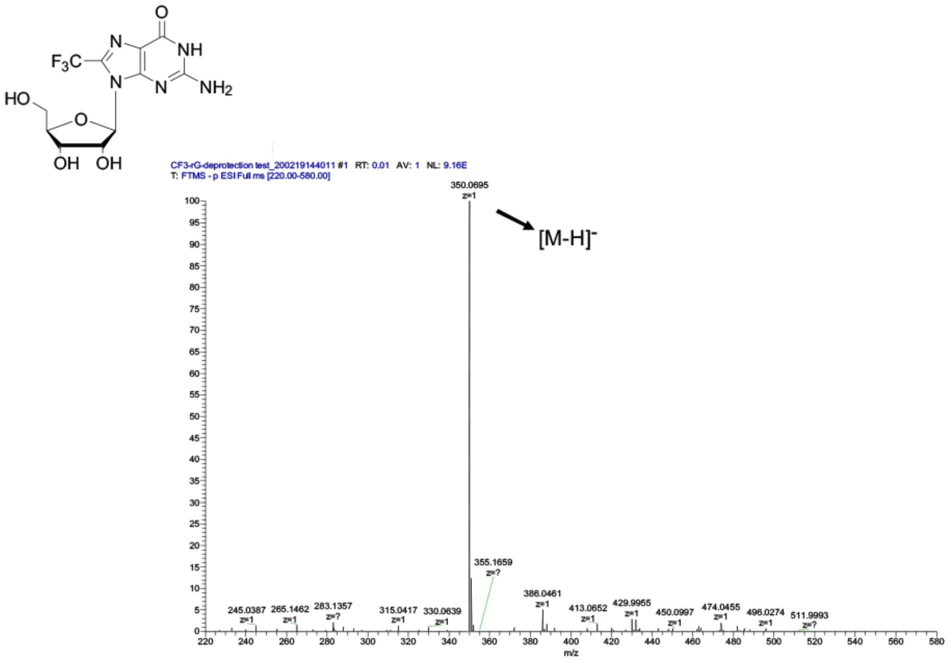


**Supplementary Figure 16.** HRMS spectrum of 8-trifluoromethylguanosine (**3**).

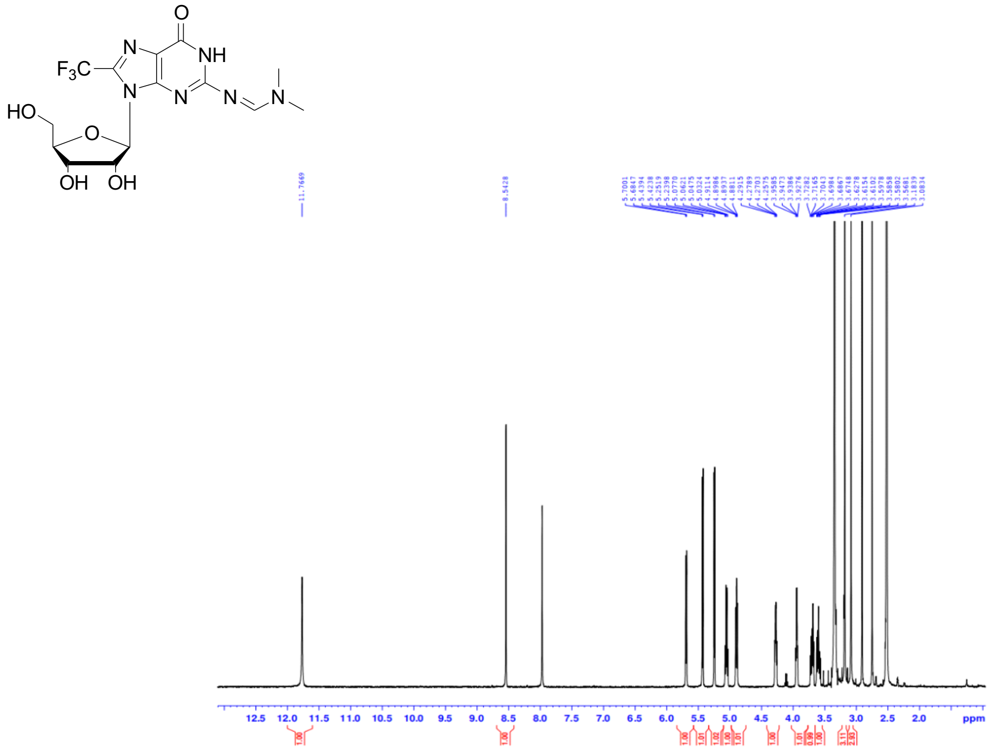


**Supplementary Figure 17.** $^{1}$H NMR spectrum of *N2*-dimethylformamidyl-8-trifluoromethylguanosine (**4**).

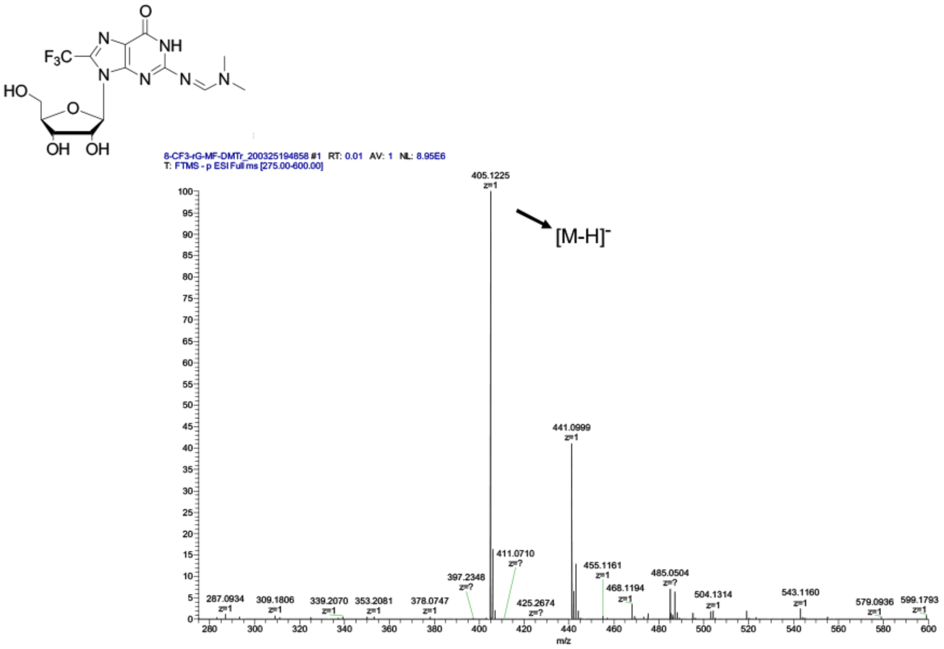


**Supplementary Figure 18.** HRMS spectrum of *N2*-dimethylformamidyl-8-trifluoromethylguanosine (**4**).

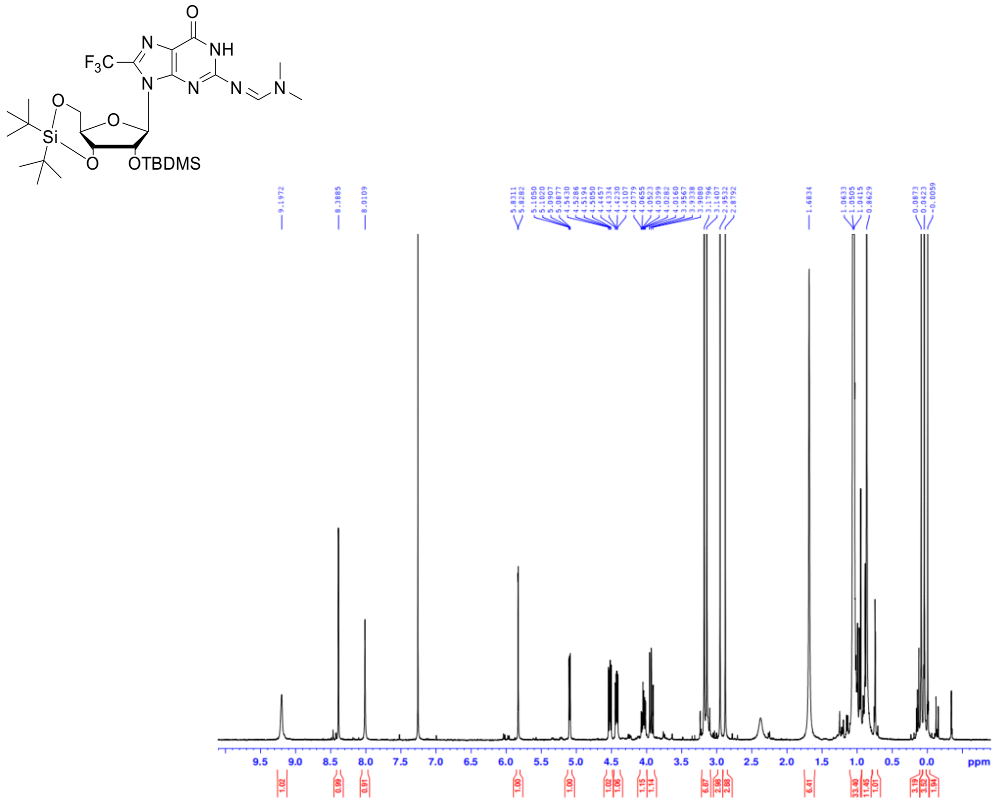


**Supplementary Figure 19.** $^{1}$H NMR spectrum of *N2*-dimethylformamidyl-8-trifluoromethyl-2'-*O*-(tert-butyldimethylsilyl)-3',5'-*O*-(di-tert-butylsilylene)guanosine (**5**).

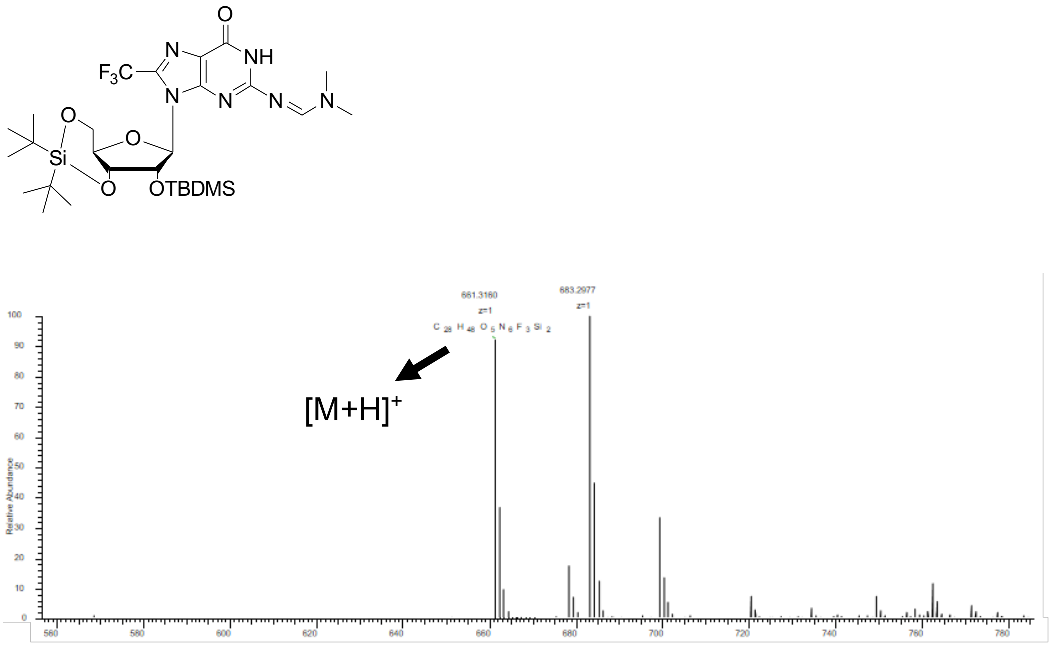


**Supplementary Figure 20.** HRMS spectrum of *N2*-dimethylformamidyl-8-trifluoromethyl-2'-*O*-(tert-butyldimethylsilyl)-3',5'-*O*-(di-tert-butylsilylene)guanosine (**5**).

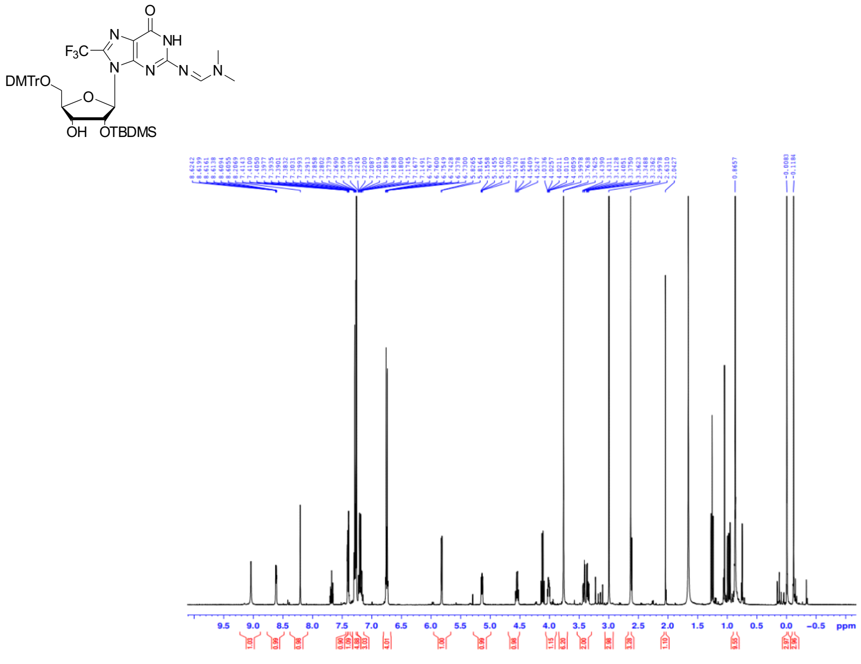


**Supplementary Figure 21.** $^{1}$H NMR spectrum of *N2*-dimethylformamidyl-8-trifluoromethyl-5'-*O*-(4,4'-dimethoxytrityl)-2'-*O*-tert-butyldimethylsilylguanosine (**6**).

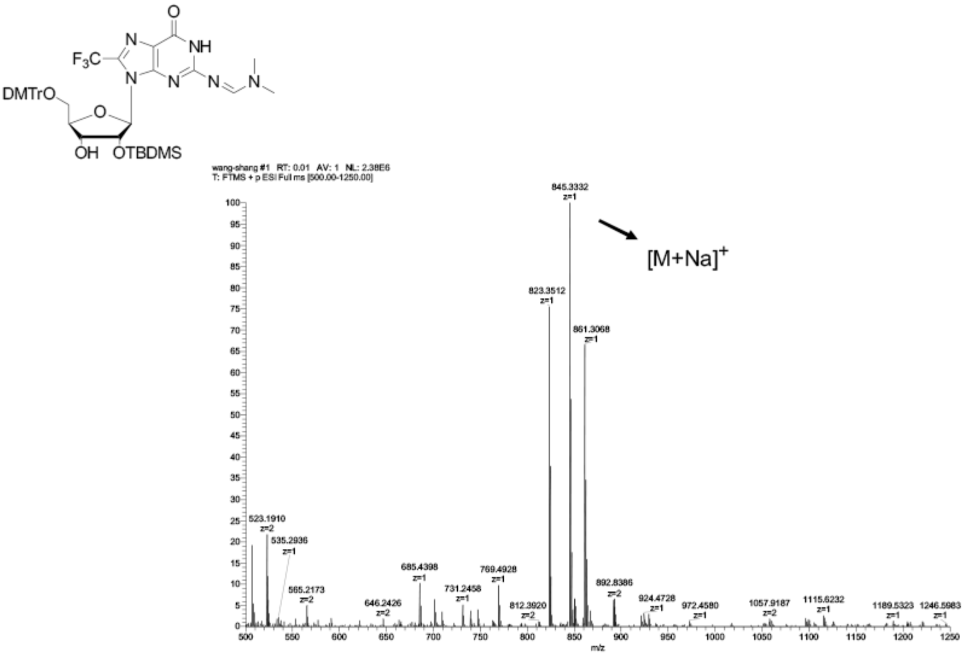


**Supplementary Figure 22.** HRMS spectrum of *N2*-dimethylformamidyl-8-trifluoromethyl-5'-*O*-(4,4'-dimethoxytrityl)-2'-*O*-tert-butyldimethylsilylguanosine (**6**).

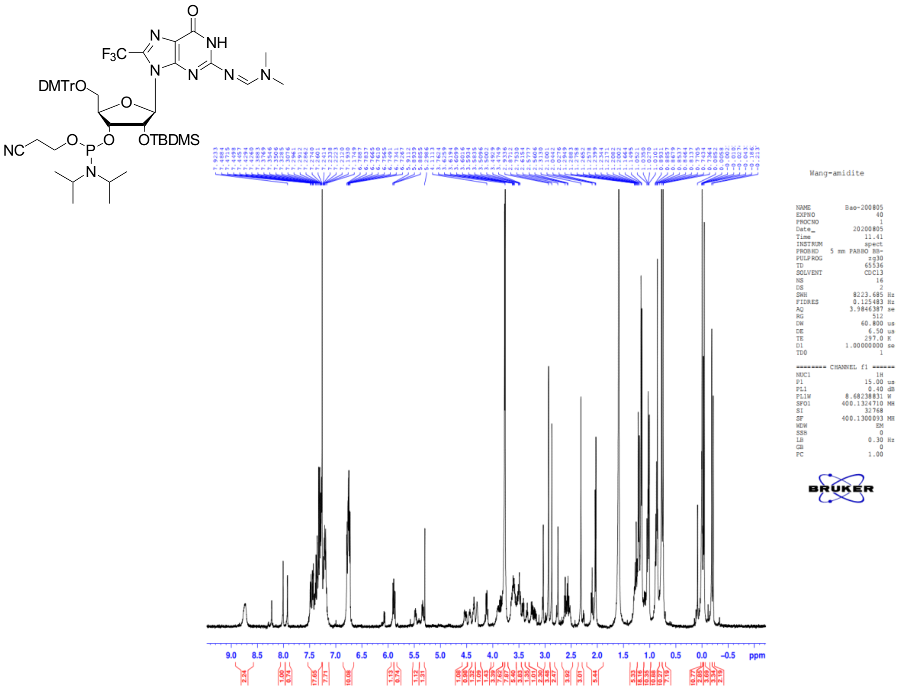


**Supplementary Figure 23.** $^{1}$H NMR spectrum of 3'-*O*-[(2-Cyanoethoxy)(diisopropylamino)phosphino]-*N2*-dimethylformamidyl-8-trifluoromethyl-5'-*O*-(4,4'-dimethoxytrityl)-2'-*O*-tert-butyldimethylsilylguanosine (**7**).

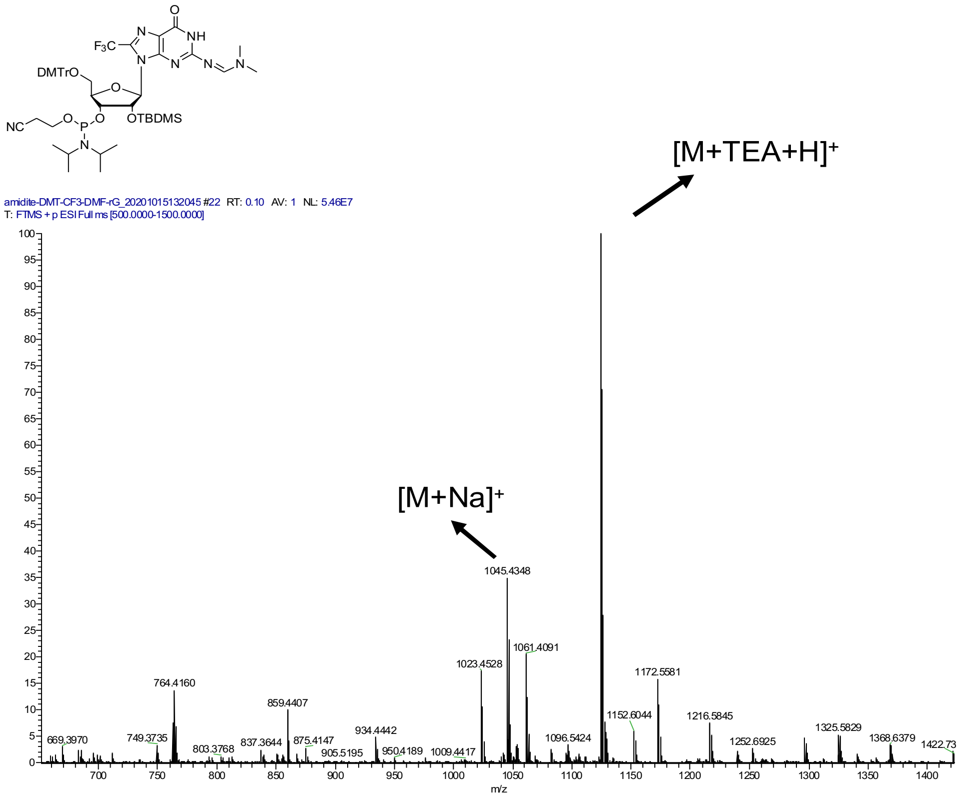


**Supplementary Figure 24.** HRMS spectrum of 3'-*O*-[(2-Cyanoethoxy)(diisopropylamino)phosphino]-*N2*-dimethylformamidyl-8-trifluoromethyl-5'-*O*-(4,4'-dimethoxytrityl)-2'-*O*-tert-butyldimethylsilylguanosine (**7**).

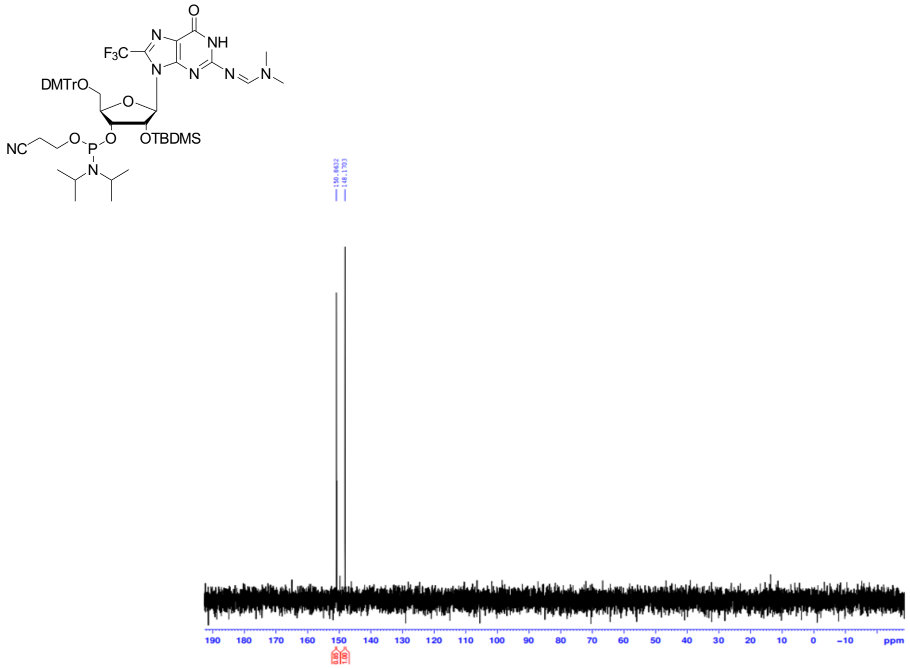


**Supplementary Figure 25.** $^{31}$P NMR spectrum of 3'-*O*-[(2-Cyanoethoxy)(diisopropylamino)phosphino]-*N2*-dimethylformamidyl-8-trifluoromethyl-5'-*O*-(4,4'-dimethoxytrityl)-2'-*O*-tert-butyldimethylsilylguanosine (**7**).

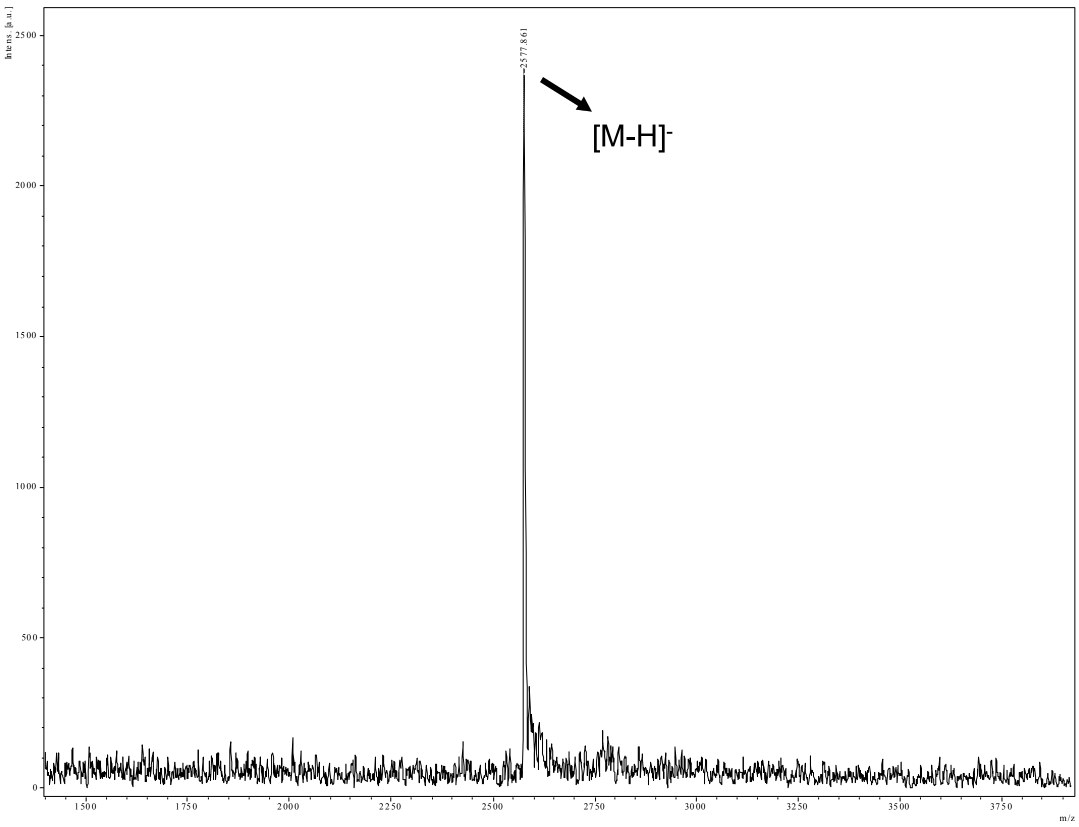


**Supplementary Figure 26.** MALDI TOF MS of r(GCGGCGGC) RNA-1. Calcd. [M-H]$^-$: 2578.634; Found 2577.861.

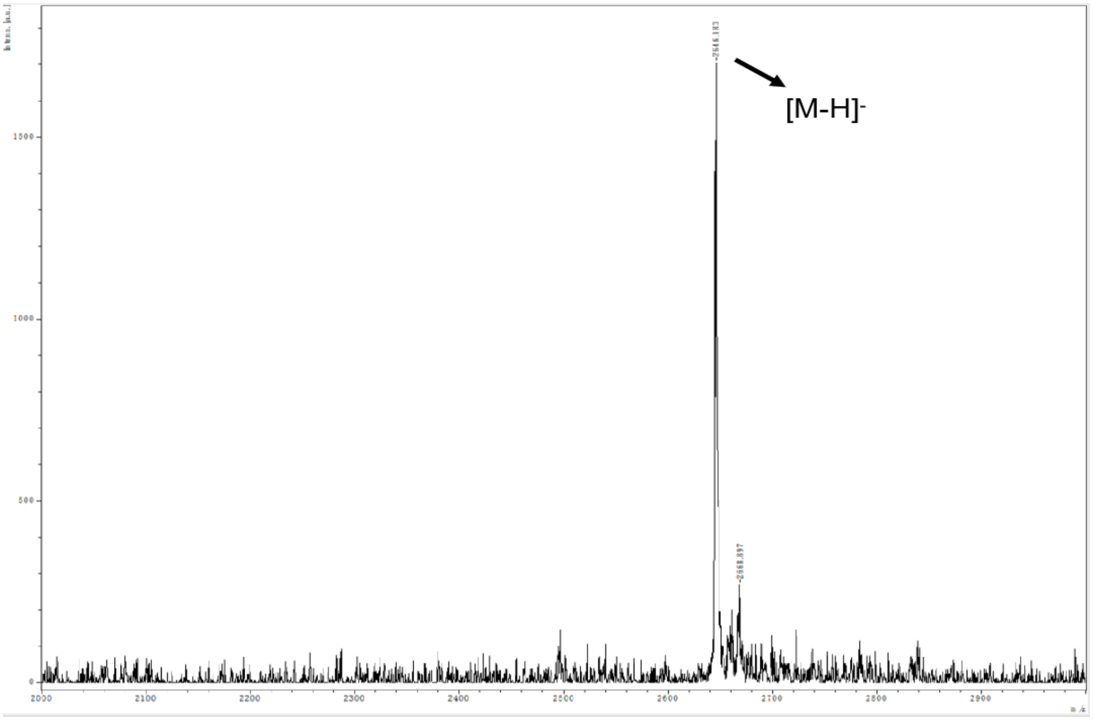


**Supplementary Figure 27.** MALDI TOF MS of r(GC$^F$GGCGGC) RNA-2. Calcd. [M-H]$^-$: 2646.894; Found 2646.183.

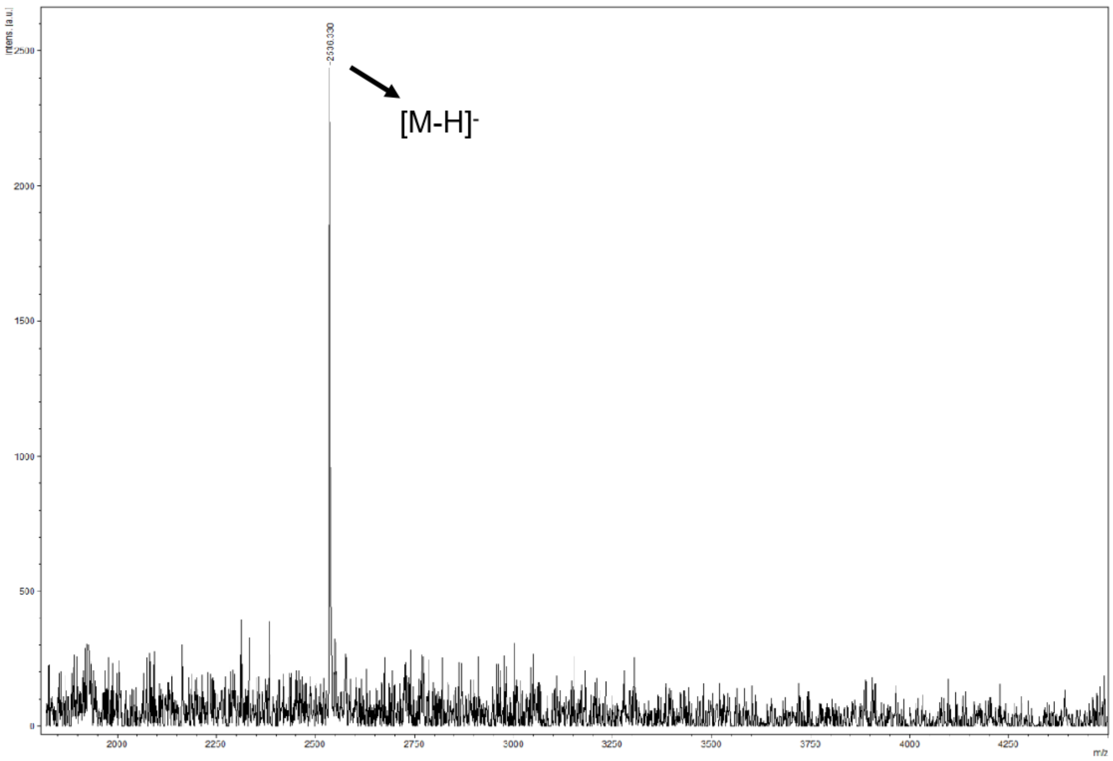


**Supplementary Figure 28:** MALDI TOF MS of r(CCGGGGCC) RNA-3.Calcd. [M-H]$^-$: 2537.897; Found 2536.330.

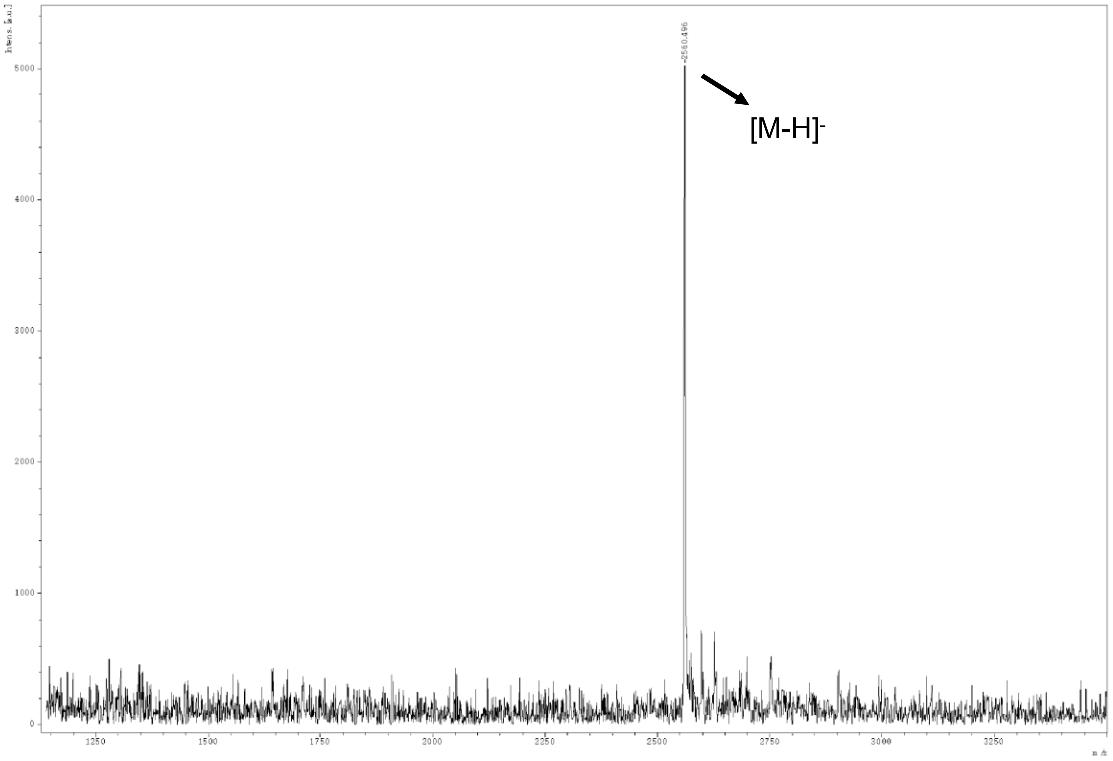


**Supplementary Figure 29.** MALDI TOF MS of r(GCGGCAGC) RNA-4. Calcd. [M-H]$^-$: 2561.605; Found 2560.496.

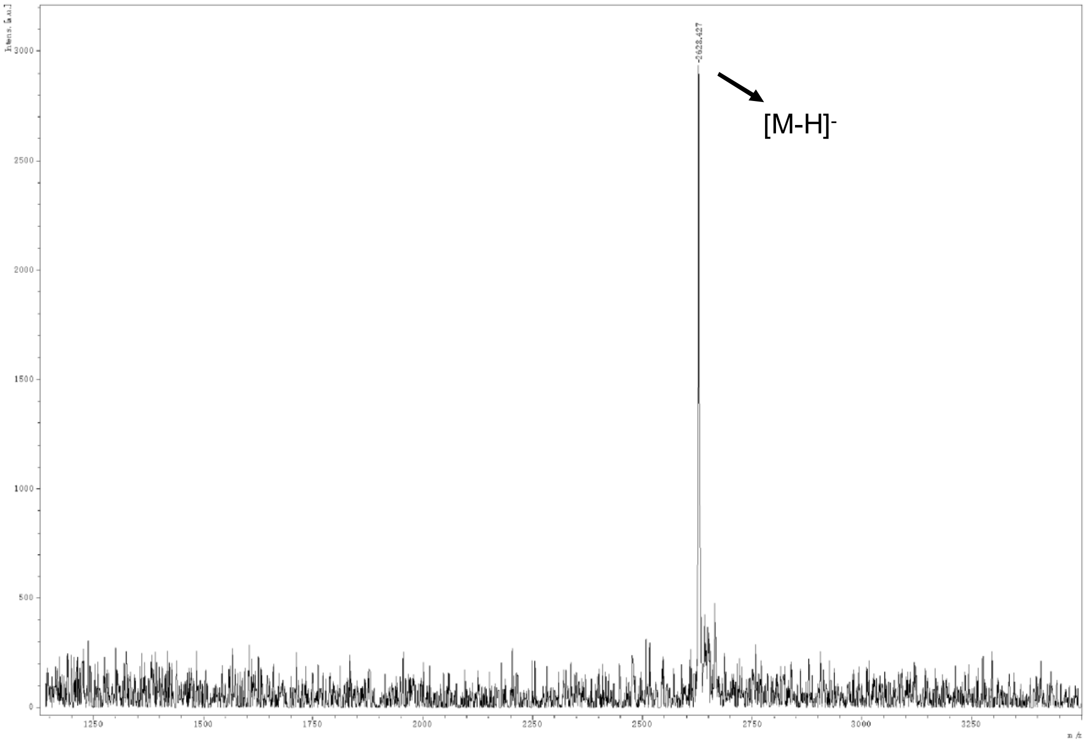


**Supplementary Figure 30.** MALDI TOF MS of r(GC$^{F}$GGCAGC) RNA-5. Calcd. [M-H]$^{-}$: 2629.096; Found 2628.427.

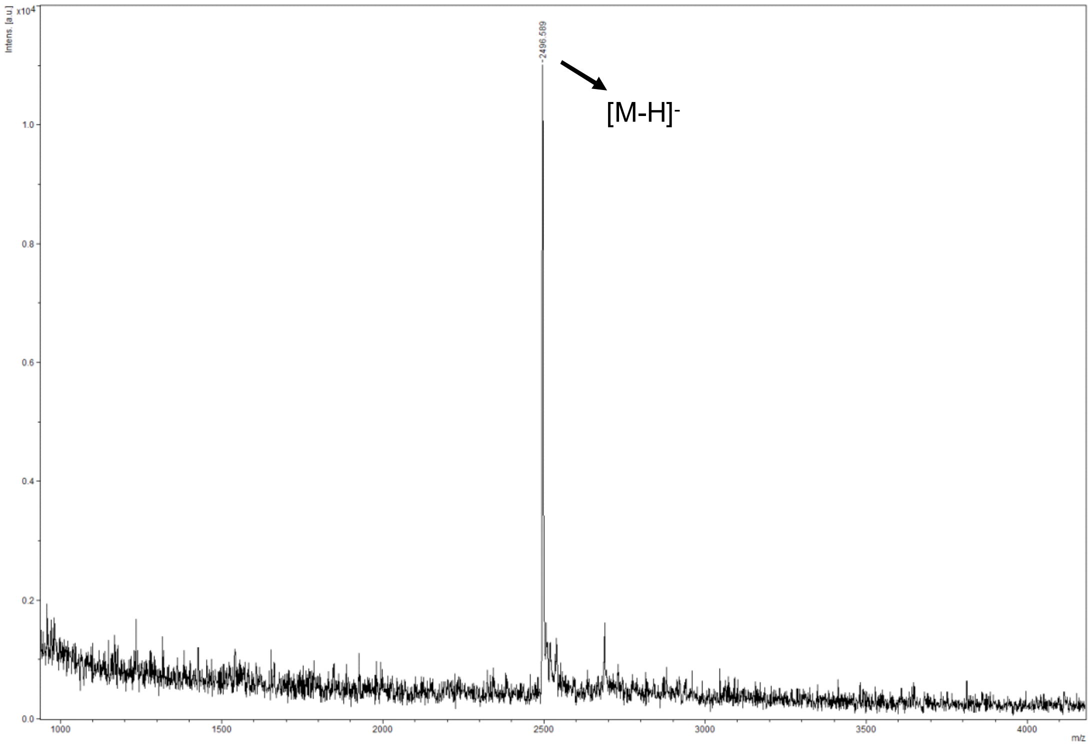


**Supplementary Figure 31.** MALDI TOF MS of ASO r(CGCCGCCG). Calcd. [M-H]$^{-}$: 2496.413; Found 2496.589.

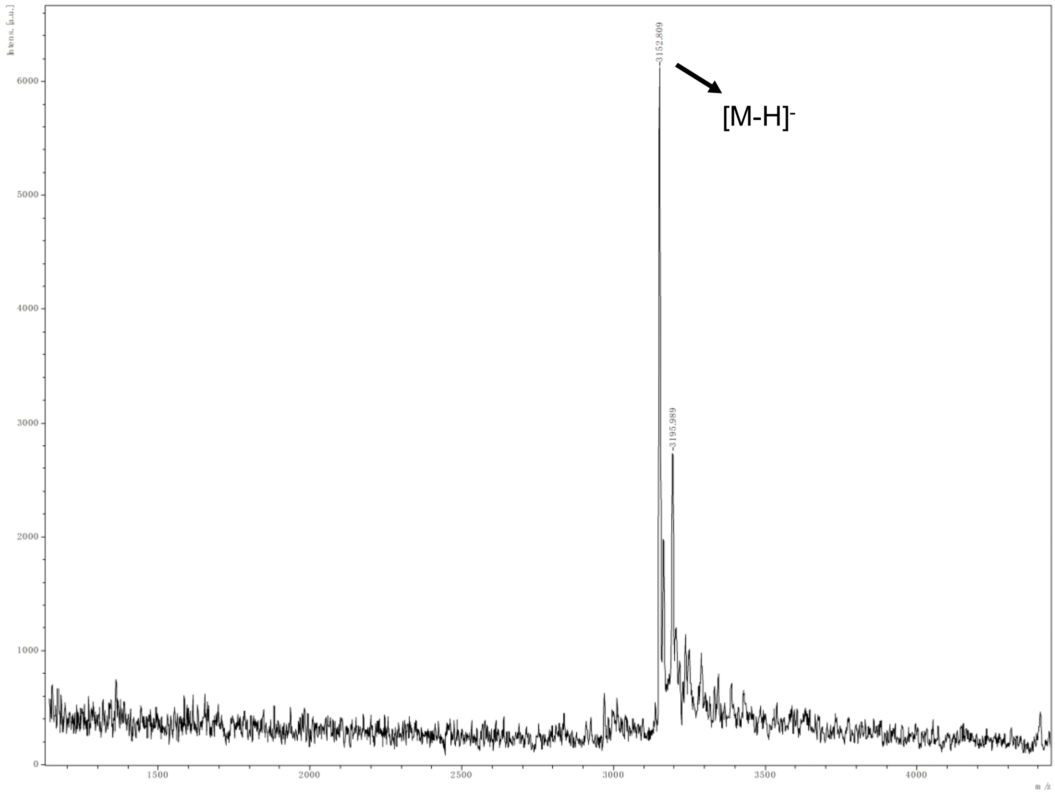


**Supplementary Figure 32.** MALDI TOF MS of Cy3-r(GCGGCGGC) FRNA-1. Calcd. [M-H]$^-$: 3153.474; Found 3152.809.

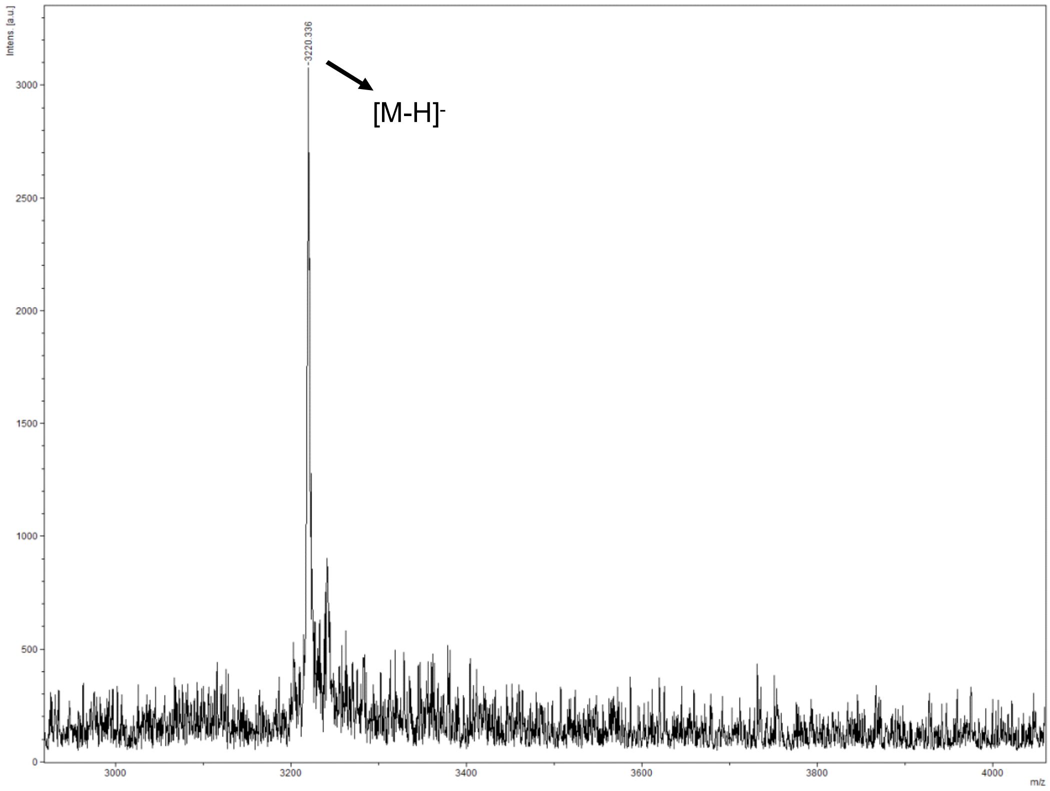


**Supplementary Figure 33.** MALDI TOF MS of Cy3-r(GC$^F$GGCGGC) FRNA-2. Calcd. [M-H]$^-$: 3221.185; Found 3220.336.

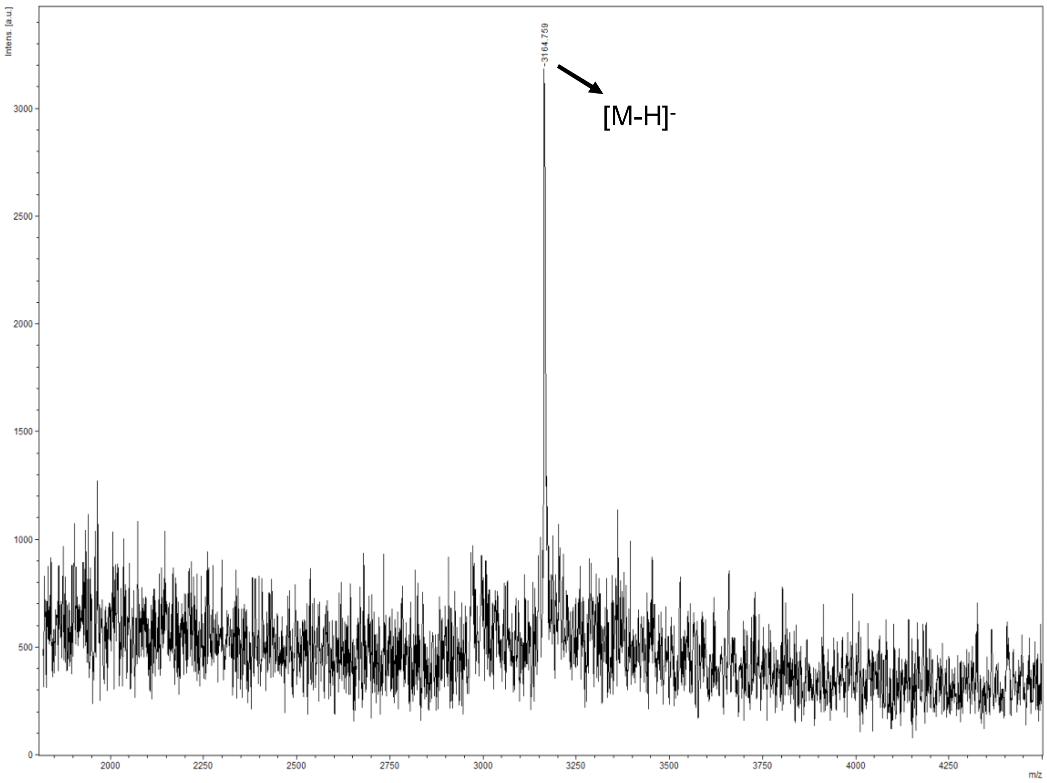


**Supplementary Figure 34.** MALDI TOF MS of Cy3-r(CCGGGGCC) FRNA-3. Calcd. [M-H]$^-$: 3163.298; Found 3164.759.

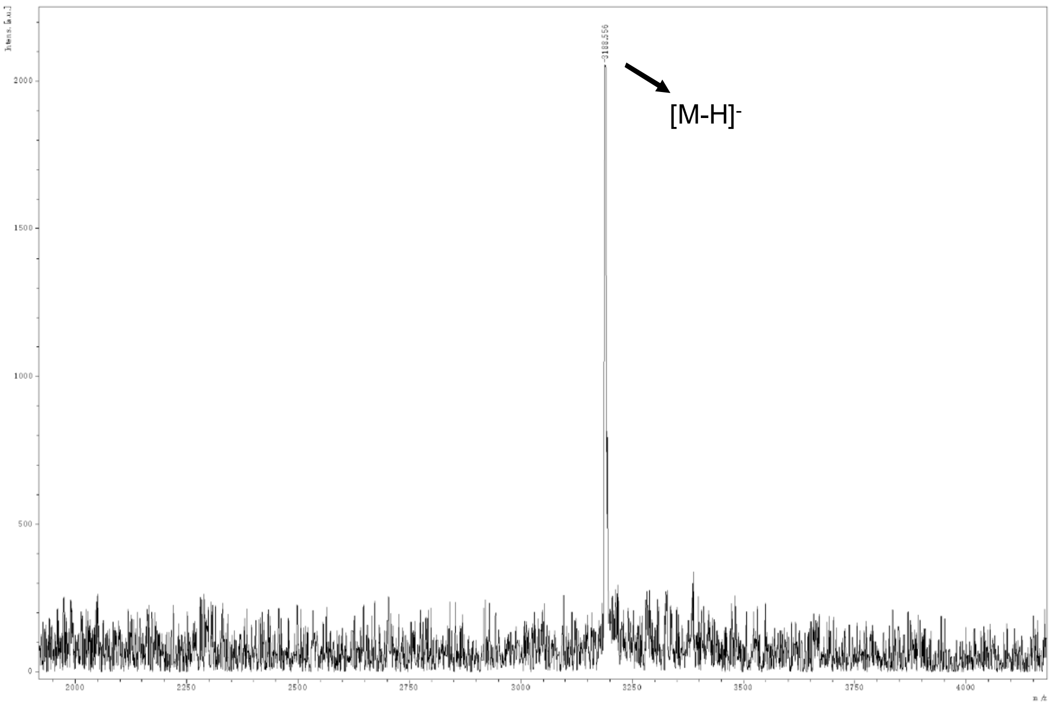


**Supplementary Figure 35.** MALDI TOF MS of Cy3-r(GCGGCAGC) FRNA-4. Calcd. [M-H]$^-$: 3189.662; Found 3188.556.

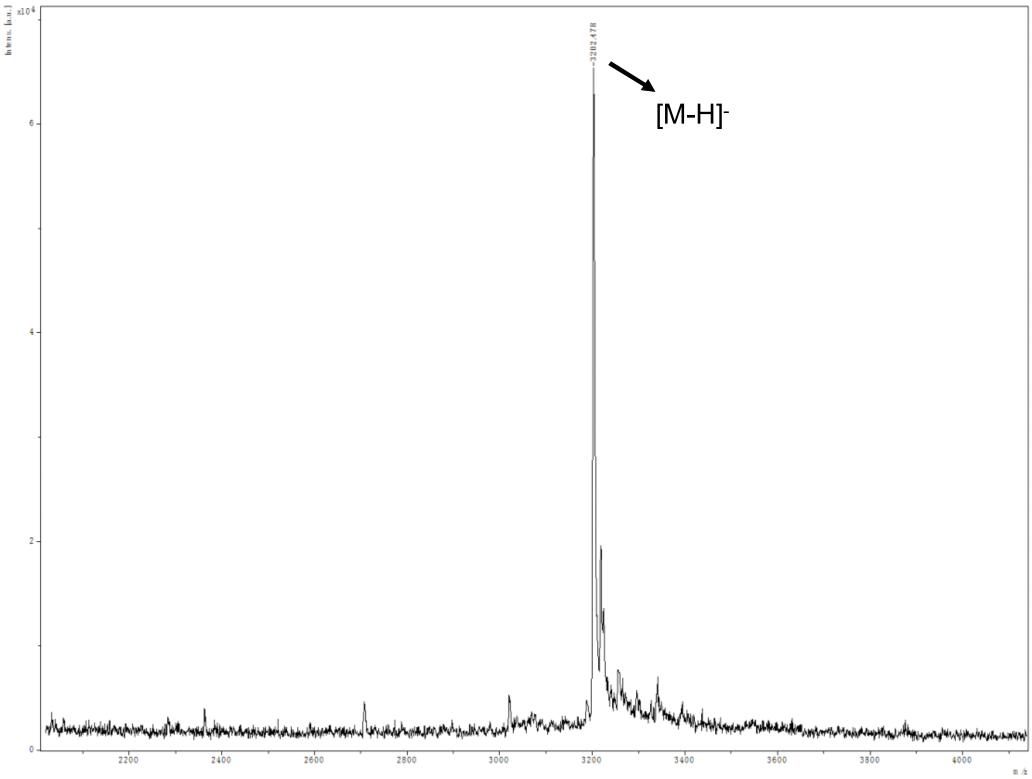


**Supplementary Figure 36.** MALDI TOF MS of Cy3-r(GC$^{F}$GGCAGC) FRNA-5. Calcd. [M-H]$^{-}$: 3203.749; Found 3202.478.

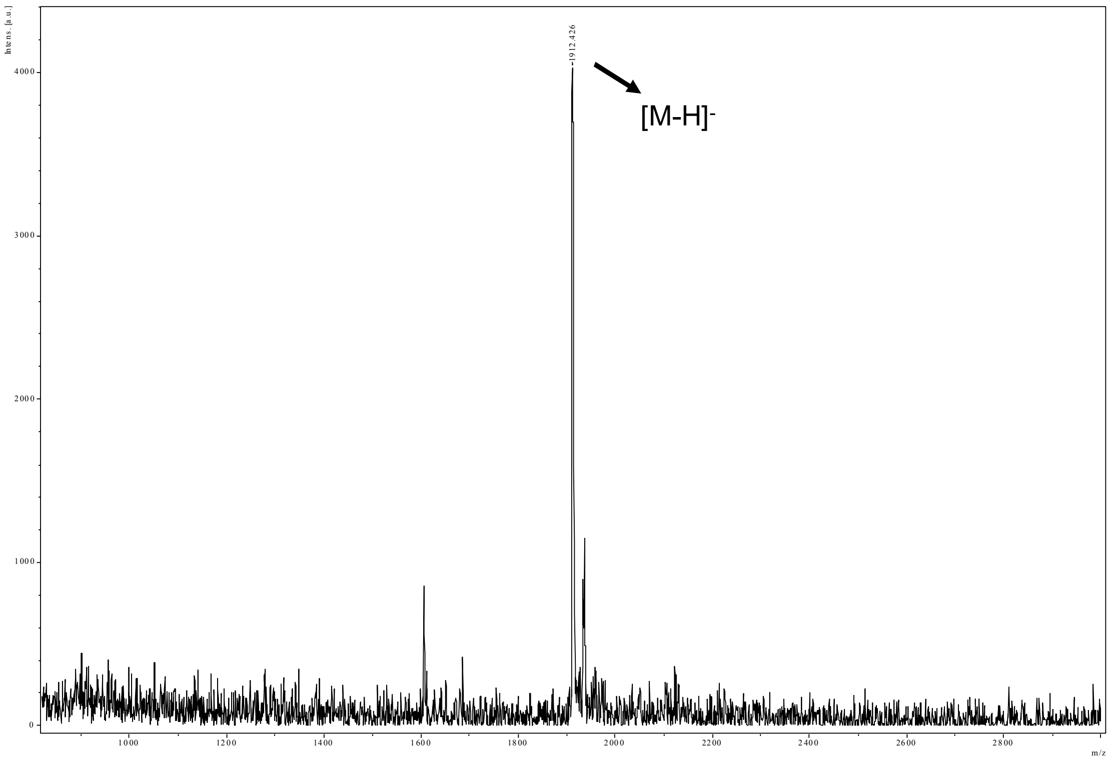


**Supplementary Figure 37.** MALDI TOF MS of r(UAGGGU). Calcd. [M-H]$^{-}$: 1913.095; Found 1912.426.

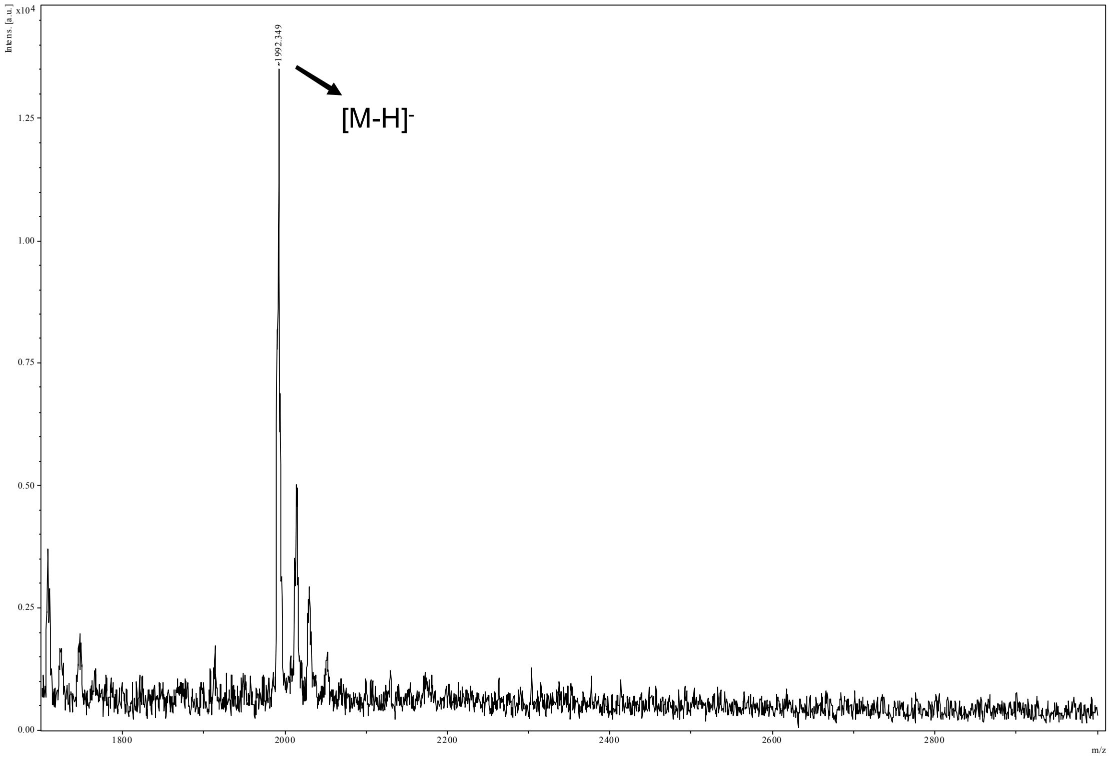


**Supplementary Figure 38.** MALDI TOF MS of r(UAG$^{Br}$GGU). Calcd. [M-H]$^-$: 1992.115; Found 1992.349.

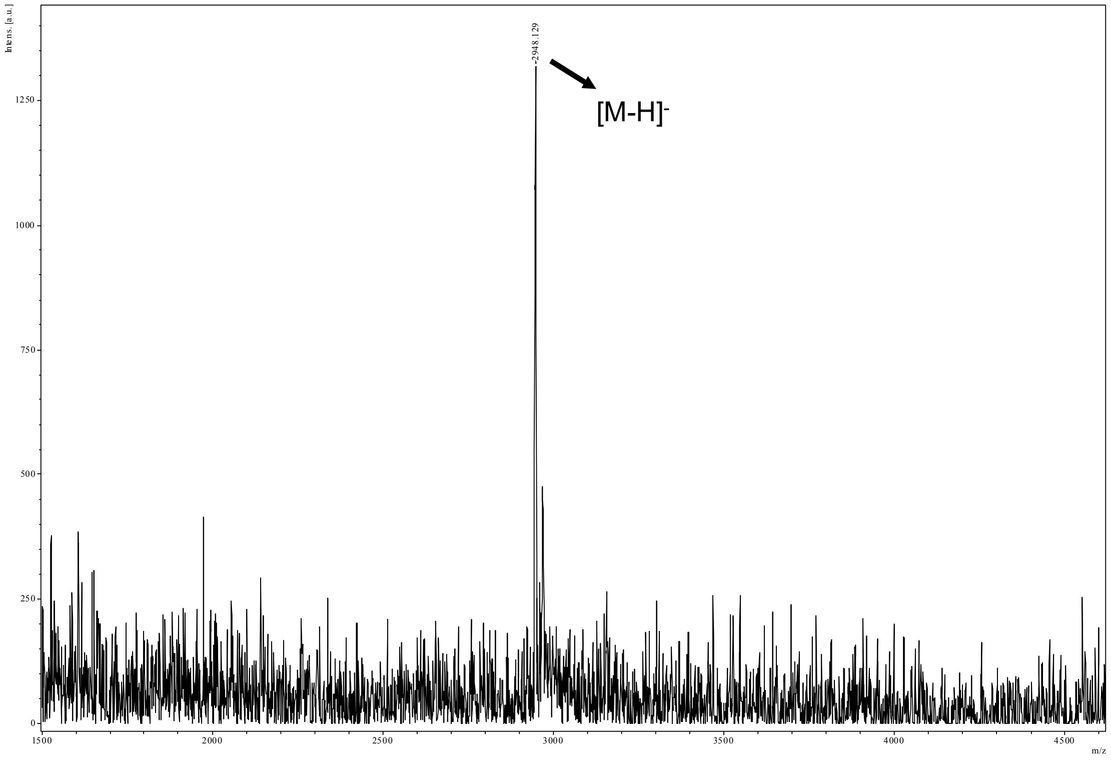


**Supplementary Figure 39.** MALDI TOF MS of r(GGGUUAGGG). Calcd. [M-H]$^-$: 2948.528; Found 2948.129.

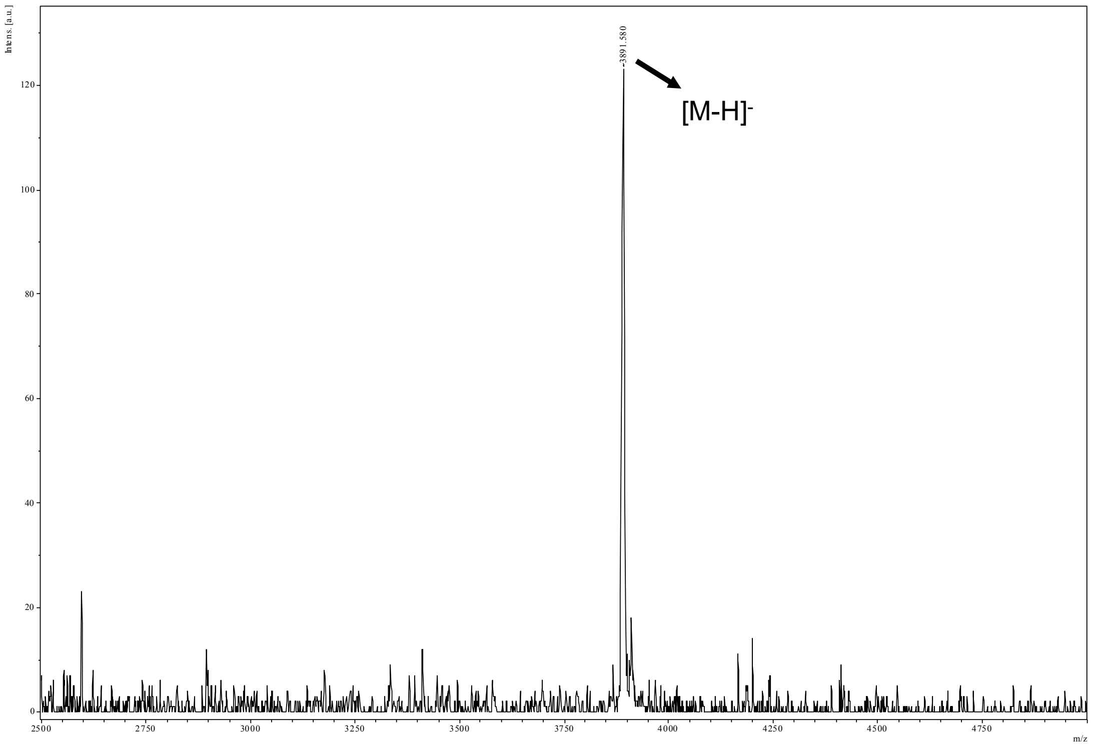


**Supplementary Figure 40.** MALDI TOF MS of r(UAGGGU)$_2$. Calcd. [M-H]$^-$: 3891.547; Found 3891.580.

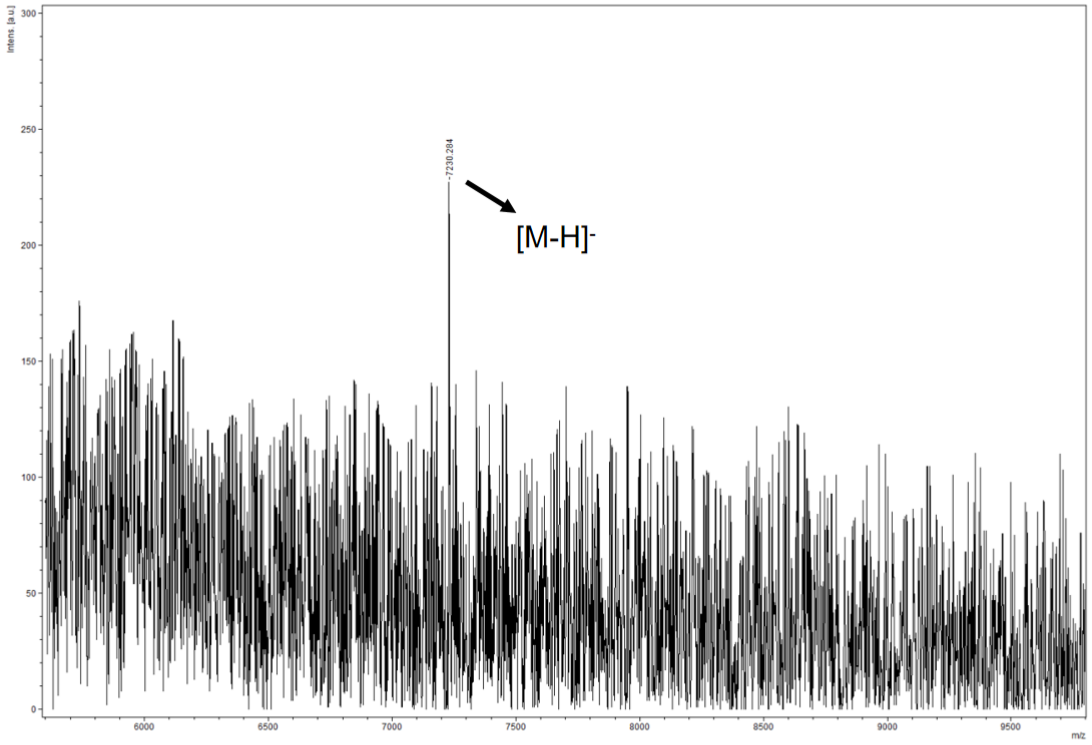


**Supplementary Figure 41.** MALDI TOF MS of rAGGG(UUAGGG)$_3$. Calcd. [M-H]$^-$: 7232.095; Found 7230.284.

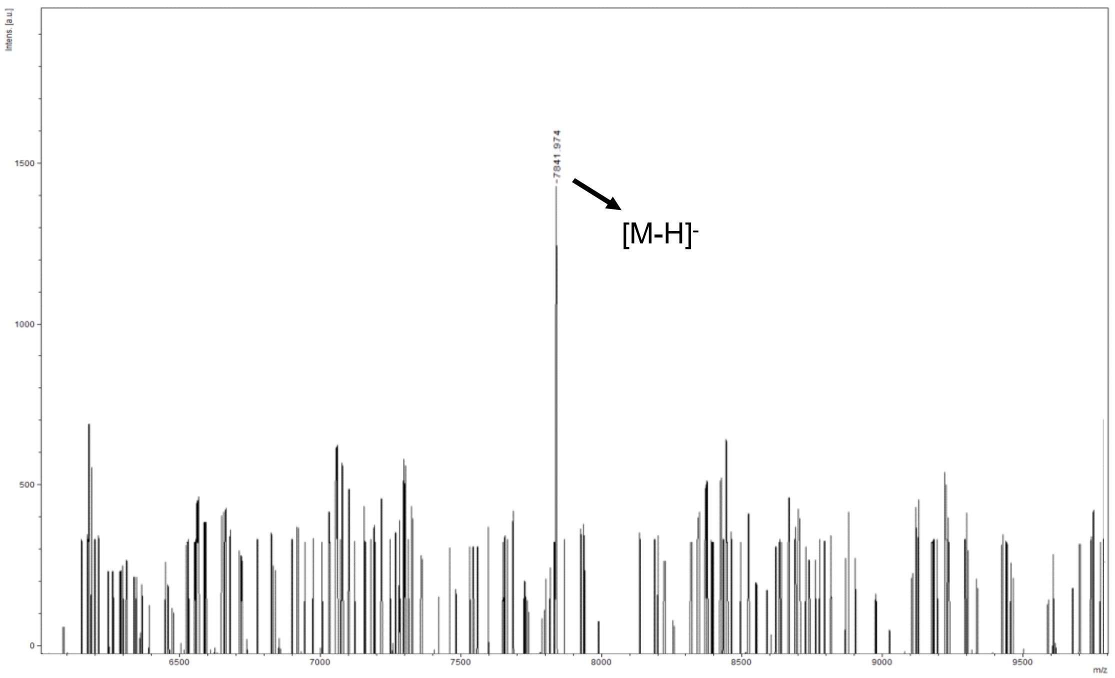


**Supplementary Figure 42.** MALDI TOF MS of r(UAGGGU)$_4$. Calcd. [M-H]$^-$: 7843.679; Found 7841.974.
