# Supplementary material for: RNA structure promotes liquid-to-solid phase transition of short RNAs in neuronal dysfunction
Source: Commun Biol. 2024 Jan 29;7:137. doi: 10.1038/s42003-024-05828-z (PMC10824717; doi:10.1038/s42003-024-05828-z)
Supplement: Supplementary file 2 — Description of Additional Supplementary Files [file 42003_2024_5828_MOESM2_ESM.pdf]

Description of Additional Supplementary Files

File name: Supplementary Data 1
Description: Source data for graph from Main manuscript file.

File name: Supplementary Data 2
Description: Source data for graph from Supplementary Information.

File name: Supplementary Movie 1
Description: The clusters of FRNA-1 with 10% PEG were photobleached and the fluorescence was monitored over 60 s. Scale bar is 2.5 μm.

File name: Supplementary Movie 2
Description: The clusters of FRNA-1 with 40% PEG were photobleached and the fluorescence was monitored over 60 s. Scale bar is 2.5 μm.

File name: Supplementary Movie 3
Description: The clusters of FRNA-2 with 10% PEG were photobleached and the fluorescence was monitored over 60 s. Scale bar is 2.5 μm.

File name: Supplementary Movie 4
Description: The clusters of FRNA-2 with 40% PEG were photobleached and the fluorescence was monitored over 60 s. Scale bar is 2.5 μm.

File name: Supplementary Movie 5
Description: The clusters of FRNA-1 in 40% PEG were treated with 1 mM doxorubicin, subsequently photobleached and the fluorescence was monitored over 60 s. Scale bar is 2.5 μm.

File name: Supplementary Movie 6
Description: The clusters of FRNA-2 in 40% PEG were treated with 1 mM doxorubicin, subsequently photobleached and the fluorescence was monitored over 60 s. Scale bar is 5 μm.

File name: Supplementary Movie 7
Description: The clusters of FRNA-1 in 40% PEG were treated with 10% 1,6- hexanediol, subsequently photobleached and the fluorescence was monitored over 60 s. Scale bar is 5 μm.

File name: Supplementary Movie 8
Description: The clusters of FRNA-2 in 40% PEG were treated with 10% 1,6- hexanediol, subsequently photobleached and the fluorescence was monitored over 60 s. Scale bar is 2.5 μm.

File name: Supplementary Movie 9
Description: The clusters of FRNA-1 in 10% PEG were treated with 1 mM doxorubicin, subsequently photobleached and the fluorescence was monitored over 60 s. Scale bar is 2.5 μm.

File name: Supplementary Movie 10
Description: The clusters of FRNA-2 in 10% PEG were treated with 1 mM doxorubicin, subsequently photobleached and the fluorescence was monitored over 60 s. Scale bar is 5 μm.

File name: Supplementary Movie 11
Description: The clusters of FRNA-1 in 10% PEG were treated with 10% 1,6- hexanediol, subsequently photobleached and the fluorescence was monitored over 60 s. Scale bar is 5 μm.

File name: Supplementary Movie 12
Description: The clusters of FRNA-2 in 10% PEG were treated with 10% 1,6- hexanediol, subsequently photobleached and the fluorescence was monitored over 60 s. Scale bar is 5 μm.

File name: Supplementary Movie 13
Description: The FRNA-1 foci in living HeLa cell were photobleached and the fluorescence was monitored over 60 s. Scale bar is 2.5 μm.

File name: Supplementary Movie 14
Description: The FRNA-2 foci in living HeLa cell were photobleached and the fluorescence was monitored over 60 s. Scale bar is 10 μm.

File name: Supplementary Movie 15
Description: The FRNA-1 foci in living HeLa cell were treated with 10% 1,6-hexanediol and photobleached. The fluorescence was monitored over 60 s. Scale bar is 5 μm.

File name: Supplementary Movie 16
Description: The FRNA-2 foci in living HeLa cell were treated with 10% 1,6-hexanediol and photobleached. The fluorescence was monitored over 60 s. Scale bar is 5 μm.

File name: Supplementary Movie 17
Description: The FRNA-1 foci in living HeLa cell were treated with 2 μM doxorubicin and photobleached. The fluorescence was monitored over 60 s. Scale bar is 2.5 μm.

File name: Supplementary Movie 18
Description: The FRNA-2 foci in living HeLa cell were treated with 2 μM doxorubicin and photobleached. The fluorescence was monitored over 60 s. Scale bar is 2.5 μm.
