# Supplementary material for: RNA structure promotes liquid-to-solid phase transition of short RNAs in neuronal dysfunction
Source: Commun Biol. 2024 Jan 29;7:137. doi: 10.1038/s42003-024-05828-z (PMC10824717; doi:10.1038/s42003-024-05828-z)
Supplement: Supplementary file 6 — Reporting Summary [file 42003_2024_5828_MOESM6_ESM.pdf]

nature portfolio

Corresponding author(s): Yan Xu

Last updated by author(s): Jan. 10, 2024

Reporting Summary

Nature Portfolio wishes to improve the reproducibility of the work that we publish. This form provides structure for consistency and transparency in reporting. For further information on Nature Portfolio policies, see our Editorial Policies and the Editorial Policy Checklist.

Statistics

For all statistical analyses, confirm that the following items are present in the figure legend, table legend, main text, or Methods section.

n/a	Confirmed	
☒	☐	The exact sample size (n) for each experimental group/condition, given as a discrete number and unit of measurement
☒	☐	A statement on whether measurements were taken from distinct samples or whether the same sample was measured repeatedly
☐	☒	The statistical test(s) used AND whether they are one- or two-sided<br>*Only common tests should be described solely by name; describe more complex techniques in the Methods section.*
☐	☒	A description of all covariates tested
☐	☒	A description of any assumptions or corrections, such as tests of normality and adjustment for multiple comparisons
☐	☒	A full description of the statistical parameters including central tendency (e.g. means) or other basic estimates (e.g. regression coefficient) AND variation (e.g. standard deviation) or associated estimates of uncertainty (e.g. confidence intervals)
☐	☒	For null hypothesis testing, the test statistic (e.g. F, t, r) with confidence intervals, effect sizes, degrees of freedom and P value noted<br>*Give P values as exact values whenever suitable.*
☒	☐	For Bayesian analysis, information on the choice of priors and Markov chain Monte Carlo settings
☒	☐	For hierarchical and complex designs, identification of the appropriate level for tests and full reporting of outcomes
☒	☐	Estimates of effect sizes (e.g. Cohen's d, Pearson's r), indicating how they were calculated

Our web collection on statistics for biologists contains articles on many of the points above.

Software and code

Policy information about availability of computer code

Data collection	BIOVIA Discovery Studio 2018, Leica Application Suite X (v5.1.0).
Data analysis	ImageJ-win64 (Java1.8.0_322), Chemdraw (v14), Topspin (v3.6.3), Microsoft Office excel 2016, OriginPro (v9.1), Lumazone SlideBook (v6.0, x64), MestReNova-11.0.4-18998.

For manuscripts utilizing custom algorithms or software that are central to the research but not yet described in published literature, software must be made available to editors and reviewers. We strongly encourage code deposition in a community repository (e.g. GitHub). See the Nature Portfolio guidelines for submitting code & software for further information.

Data

Policy information about availability of data

All manuscripts must include a data availability statement. This statement should provide the following information, where applicable:

- Accession codes, unique identifiers, or web links for publicly available datasets
- A description of any restrictions on data availability
- For clinical datasets or third party data, please ensure that the statement adheres to our policy

1. Number of biological and technical repeats.
For the study of RNA phase separation, gelation and solidification in vitro, in each group experiments, 3 independent experiments were completed and more than 20 images obtained in each result. For the study of RNA aggregation in living cells, in each group experiments, 3 independent experiments were completed and

more than 10 images obtained in each result .For the study of FRAP assay, in each group experiments, 3 independent experiments were done and more than 30 images obtained as time dependence in each result. For the study of RNA aggregation dysfunction in cell, in each group experiments, 3 independent experiments were completed and more than 10 images obtained in each result. For the study of RNA aggregates on clonogenic capacity, 3 independent experiments were completed and 3 images obtained in each result.
For these techniques used in this study including UV, CD, NMR and gel electrophoresis, single experiment was performed.

2. Total number of images (and cells where applicable) acquired and assessed for each graph/figure.
For the study of RNA phase separation, gelation and solidification in vitro, in each group experiments, at least 10 images that were randomly selected from the totally obtained confocal images more than 20 pieces, in which the most representative one was divided into at least 4 subunits with equal size and the most representative one finally defined as presentation. For the study of RNA aggregation in living cells, in each group experiments, at least 5 images that were randomly selected from the totally obtained confocal images more than 10 pieces containing intact cells, in which the representative images was divided into at least 16 subunits with equal size and the most representative one finally defined as presentation. For the study of FRAP assay, in each group experiments, 3 or 4 images at respective scheduled time point were selected from total more than 30 images during the entire analysis profile around 60 s, and presented. For the study of RNA aggregation dysfunction in cell, in each group experiments, at least 5 images that were randomly selected from the totally obtained confocal images more than 10 pieces containing intact cells, in which the representative images was divided into at least 4 subunits with equal size and the most representative one finally defined as presentation. For the study of RNA aggregates on clonogenic capacity, in each group experiments, 1 representative image that was randomly selected from the totally obtained 3 confocal images and defined as presentation.

3. Single optical slices and z projections.
In this study, max intensity z-projection and a single 0.5-µm optical slice was used for all images.

4. Image optimization/processing.
All images presented in figure as first quarter, quarter or one sixteenth subunit from uniformly trimmed originally image without further optimization.

Research involving human participants, their data, or biological material

Policy information about studies with human participants or human data. See also policy information about sex, gender (identity/presentation), and sexual orientation and race, ethnicity and racism.

Reporting on sex and gender	No data
Reporting on race, ethnicity, or other socially relevant groupings	No data
Population characteristics	No data
Recruitment	No data
Ethics oversight	No data

Note that full information on the approval of the study protocol must also be provided in the manuscript.

Field-specific reporting

Please select the one below that is the best fit for your research. If you are not sure, read the appropriate sections before making your selection.

☒ Life sciences ☐ Behavioural & social sciences ☐ Ecological, evolutionary & environmental sciences

For a reference copy of the document with all sections, see nature.com/documents/nr-reporting-summary-flat.pdf

Life sciences study design

All studies must disclose on these points even when the disclosure is negative.

Sample size	All sample size in this study were afforded directly from original images of TCS SP8 confocal microscopy (Leicamicrosystems) or amplified results according to these images with scale-up.
Data exclusions	No data were excluded.
Replication	In vitro experiments were performed with at least three independent biological replicates. All attempts at replication were successful.
Randomization	For in vivo experiments, mice were randomized before treatment
Blinding	Investigators were not blinded in any experiment.

Reporting for specific materials, systems and methods

We require information from authors about some types of materials, experimental systems and methods used in many studies. Here, indicate whether each material, system or method listed is relevant to your study. If you are not sure if a list item applies to your research, read the appropriate section before selecting a response.

Materials & experimental systems

n/a	Involved in the study
☐	☒ Antibodies
☐	☒ Eukaryotic cell lines
☒	☐ Palaeontology and archaeology
☐	☒ Animals and other organisms
☒	☐ Clinical data
☒	☐ Dual use research of concern
☒	☐ Plants

Methods

n/a	Involved in the study
☒	☐ ChIP-seq
☒	☐ Flow cytometry
☒	☐ MRI-based neuroimaging

Antibodies

Antibodies used	SAM68 (Santa Cruz, sc-514468, AF488)
Validation	The antibody has been validated by manufacturers (Santa Cruz).

Eukaryotic cell lines

Policy information about cell lines and Sex and Gender in Research

Cell line source(s)	HeLa (ATCC), Cos-7 (JCRB9127).
Authentication	All cell lines used in this study are authenticated by the manufacturers
Mycoplasma contamination	All cell lines used in this study are routinely tested for mycoplasma contamination by manufacturers, and were all negative for mycoplasma.
Commonly misidentified lines (See ICLAC register)	No commonly misidentified cell lines were used.

Animals and other research organisms

Policy information about studies involving animals; ARRIVE guidelines recommended for reporting animal research, and Sex and Gender in Research

Laboratory animals	BALB/c nude female mice (aged 6 weeks were purchased from Charles River Laboratories Japan (Yokohama, Japan).
Wild animals	This study did not use wild animals
Reporting on sex	No data
Field-collected samples	This study did not involve field-collected samples
Ethics oversight	All animal operations were carried out in compliance with the relevant laws and approved by the Institutional Animal Care and Use Committee of University of Miyazaki (Miyazaki, Japan).

Note that full information on the approval of the study protocol must also be provided in the manuscript.
